# Impact of mHealth interventions on maternal, newborn, and child health from conception to 24 months postpartum in low- and middle-income countries: a systematic review

**DOI:** 10.1186/s12916-024-03417-9

**Published:** 2024-05-15

**Authors:** Marianne Ravn Knop, Michiko Nagashima-Hayashi, Ruixi Lin, Chan Hang Saing, Mengieng Ung, Sreymom Oy, Esabelle Lo Yan Yam, Marina Zahari, Siyan Yi

**Affiliations:** 1 Saw Swee Hock School of Public Health, National University of Singapore and National University Health System, Singapore, Singapore; 2 KHANA Center for Population Health Research, Phnom Penh, Cambodia; 3 Public Health Program, College of Education and Health Sciences, Touro University California, Vallejo, CA USA

**Keywords:** Maternal and child health, Digital health, mHealth, Healthcare access, Primary care, Low- and middle-income countries

## Abstract

**Background:**

Mobile health (mHealth) technologies have been harnessed in low- and middle-income countries (LMICs) to address the intricate challenges confronting maternal, newborn, and child health (MNCH). This review aspires to scrutinize the effectiveness of mHealth interventions on MNCH outcomes during the pivotal first 1000 days of life, encompassing the period from conception through pregnancy, childbirth, and post-delivery, up to the age of 2 years.

**Methods:**

A comprehensive search was systematically conducted in May 2022 across databases, including PubMed, Cochrane Library, Embase, Cumulative Index to Nursing & Allied Health (CINAHL), Web of Science, Scopus, PsycINFO, and Trip Pro, to unearth peer-reviewed articles published between 2000 and 2022. The inclusion criteria consisted of (i) mHealth interventions directed at MNCH; (ii) study designs, including randomized controlled trials (RCTs), RCT variations, quasi-experimental designs, controlled before-and-after studies, or interrupted time series studies); (iii) reports of outcomes pertinent to the first 1000 days concept; and (iv) inclusion of participants from LMICs. Each study was screened for quality in alignment with the Cochrane Handbook for Systematic Reviews of Interventions and the Joanne Briggs Institute Critical Appraisal tools. The included articles were then analyzed and categorized into 12 mHealth functions and outcome domain categories (antenatal, delivery, and postnatal care), followed by forest plot comparisons of effect measures.

**Results:**

From the initial pool of 7119 articles, we included 131 in this review, comprising 56 RCTs, 38 cluster-RCTs, and 37 quasi-experimental studies. Notably, 62% of these articles exhibited a moderate or high risk of bias. Promisingly, mHealth strategies, such as dispatching text message reminders to women and equipping healthcare providers with digital planning and scheduling tools, exhibited the capacity to augment antenatal clinic attendance and enhance the punctuality of child immunization. However, findings regarding facility-based delivery, child immunization attendance, and infant feeding practices were inconclusive.

**Conclusions:**

This review suggests that mHealth interventions can improve antenatal care attendance and child immunization timeliness in LMICs. However, their impact on facility-based delivery and infant feeding practices varies. Nevertheless, the potential of mHealth to enhance MNCH services in resource-limited settings is promising. More context-specific implementation studies with rigorous evaluations are essential.

**Supplementary Information:**

The online version contains supplementary material available at 10.1186/s12916-024-03417-9.

## Background

Despite the significant progress in maternal and child mortality globally, large inequities persist between and within countries [1, 2]. Over 4.5 million women and babies die annually during pregnancy, childbirth, or the first weeks after birth. Most of these preventable deaths are concentrated in low- and middle-income countries (LMICs), especially among some geographical regions and populations, such as socio-economically vulnerable women in Sub-Saharan Africa and South Asia [1–3]. To address the challenge, strategies to integrate the programs across the maternal, newborn, and child health (MNCH) continuum have been adopted to lower costs while promoting greater efficiencies and reducing duplication of resources. The continuum of care strengthens healthcare quality, coverage, and affordability [4, 5], as represented in the “first 1000 days” concept [6, 7]. In LMICs, however, the degree of availability and quality of MNCH services varies considerably, and barriers, such as limited resources and poor information and communication infrastructures, compromise access to services [8].

With rapidly growing digital connectivity, the roles of mobile health technologies (mHealth) in addressing MNCH outcomes in LMICs have been recognized [9–11]. Expectations towards mHealth, in general, include its potential to improve the quality and coverage of healthcare, increase access to health information, services and skills, and promote positive changes in health behaviors to prevent the onset of acute and chronic diseases and improve treatment adherence and outcomes [10–14]. In LMICs, mHealth systems can potentially fill the critical gaps in human resources and information and communication infrastructures, reaching remote and marginalized populations and enhancing a range of low-cost life-saving interventions at the community level [11, 12, 15, 16].

Studies of the efficacy of mHealth interventions vary in their design and focus, such as types of health outcomes and domains and mHealth functions. In their systematic review of systematic reviews on mHealth interventions, Marcolino et al. revealed that the most popular and successful mHealth interventions were behavior change approaches using text messaging due to the low cost and low broadband requirements [15]. However, the authors suggested further studies be conducted with more robust designs to confirm the efficacy of mHealth interventions [15]. Studies in LMICs involving mHealth technologies have often needed more representativeness, as populations most likely to benefit from the interventions (i.e., lower-income groups, women, older people, and rural populations) were excluded, owing to the lack of access to digital technologies [11, 17, 18]. Other systematic reviews have assessed the effectiveness of diverse mHealth interventions in LMICs targeting maternal, neonatal, and infant care individually or a combination thereof [8, 19–23]. However, to the best of our knowledge, no systematic reviews have covered the MNCH spectrum, which covers a period of 1000 days from the time of conception to 2 years postpartum.

A qualitative content analysis of users’ perspectives of 75 applications for pregnant mothers and new parents revealed that women increasingly used mobile technology to improve their health literacy, monitoring, self-management, decision-making, and searching for credible information, such as how to establish breastfeeding and common infant health issues [24, 25]. Women reported using the applications for multiple pregnancies [24], implying that such interventions offer a high potential for improving MNCH outcomes. Given the crucial need for such an integrated approach in LMICs, this systematic review will provide a comprehensive overview of available evidence and understanding of research gaps in mHealth for improving the continuum of MNCH care in LMICs by synthesizing the mHealth evidence encompassing the 1000 days. This study’s findings will support the policy decision and resource allocation for future interventions and research planning in resource-constrained settings.

## Methods

This systematic review followed the Preferred Reporting Items for Systematic Reviews and Meta-Analyses (PRISMA) statement [26]. A detailed protocol has been registered with the International Prospective Register for Systematic Reviews (PROSPERO registration number: CRD42022354586).

### Eligibility criteria

Articles were included in this review if they (i) primarily evaluated an mHealth intervention targeting MNCH outcomes; (ii) were designed as a randomized controlled trial (RCT), variations of RCT, quasi-experimental study, controlled before-and-after study, and interrupted time series study; (iii) reported outcomes relevant to the first 1000 days concept; (iv) involved participants from LMICs, according to the World Bank index [27] as of May 2022; and (v) were published in a peer-reviewed journal between 01 January 2000 and 31 May 2022. We excluded studies published before the year 2000 as we focused on more contemporary forms of mHealth that employed mobile technologies to ensure the relevance of this review. Outcomes were not pre-specified, given our interest in all outcomes related to MNCH from conception to 2 years postpartum. Therefore, we reported outcomes related to pregnant women, mothers and newborns, and children under the age of 2 years. Considering the extensive literature we identified, we included only articles published in peer-reviewed journals. Peer-reviewed articles are generally regarded as providing more trusted and reliable scientific information due to their adherence to rigorous methodological standards, as opposed to non-peer-reviewed sources.

We excluded studies (i) that did not have a control group, (ii) without accessible full-texts, and (iii) that were observational, such as cohort, case–control, cross-sectional and qualitative studies, expert opinions, reviews, project/program reports, discussion papers, or case reports. Initially, we did not restrict the publication language; however, we eventually excluded one article where translation from Thai to English was unavailable. We excluded studies that evaluated the willingness of participants to receive a mHealth intervention or the mHealth tool itself, as those outcomes are not directly relevant to MNCH outcomes.

### Search strategy and information sources

We developed a systematic search strategy and quality assessment of the literature. We searched PubMed, Cochrane Library, Embase, Cumulative Index to Nursing & Allied Health (CINAHL), Web of Science, Scopus, PsycINFO, and Trip Pro in May 2022. Search terms included Medical Subject Headings (MeSH), title, abstract, and text words. The detailed search syntax can be found in Additional file 1: Table A1. We used an online Polyglot Search Translator for database platforms [28]. Trip Pro required a different search approach, as specified in Table A1. We further searched literature via the snowballing effect by (i) reviewing relevant study protocols to identify publications reporting relevant intervention outcomes, (ii) reviewing previously published systematic reviews, and (iii) screening the reference lists of all articles included in this review.

We removed duplicate articles using Endnote (version 20.3). Two primary reviewers (MRK and RL) independently screened titles, abstracts, and full-text articles of potentially eligible articles against the inclusion and exclusion criteria. MRK extracted the data, and RL reviewed them to identify the following information: study design, research methods, location and settings, target population and size, mHealth function and forms, and research findings. We resolved discrepancies in the data selection and extraction by consensus or consulting a third reviewer within the study team.

### Risk of bias assessment

MRK performed the quality assessment independently, while other team members (RL, MU, SC, SO) performed the second assessment. A third team member conducted an additional check to resolve discrepancies. We assessed intervention studies using the criteria of the Cochrane Handbook for Systematic Reviews of Interventions [29] and quasi-experimental studies using the Joanne Briggs Institute Critical Appraisal (JBI) tools [30, 31]. We assessed the quality of studies using baseline-online-comparison designs with a control group using the JBI tool for quasi-experimental studies regardless of whether a randomization process was described.

We graded the risk of bias for RCTs into three levels (low, moderate, or high). Quasi-experimental studies received a grade according to the scale they were evaluated against. We considered the risk of bias in determining the strength of the conclusion [29].

### Analysis and synthesis

We conducted systematic narrative and descriptive analyses of the 131 included articles [32–162] to capture the main characteristics of each study by mapping out the study designs, settings, population groups and sizes, intervention and control groups, outcome measures and results, outcome domains, and mHealth forms and functions (Additional file 2: Table A2). For each study, at least two other authors further reviewed the analyzed characteristics and assigned categories to ensure consistency and rigor.

#### mHealth functions

We categorized the mHealth strategies adopted in each study into 12 mHealth functions described by Labrique et al. [163]. The 12 functions are (1) client education and behavior change communication (BCC), (2) sensors and point-of-care diagnostics, (3) registries and vital events tracking, (4) data collection and reporting, (5) electronic health records, (6) electronic decision support, (7) provider-to-provider communication, (8) provider work planning and scheduling, (9) provider training and education, (10) human resource management, (11) supply chain management, and (12) financial transactions and incentives. We further categorized the studies according to the outcomes measured under each health domain (antenatal care [ANC], delivery care, and postnatal care [PNC]).

#### Outcome domain categories

We categorized the intervention outcomes into three categories according to the relevant care period within the 1000-day timeframe (i.e., ANC, delivery care, and PNC). ANC included the outcomes such as the number of ANC visits, maternal micronutrient supplementation, medical treatment encompassing tetanus toxoid injection, and compliance to any prescribed procedures and tests (e.g., ultrasound examination, oral glucose tolerance test, urine tests, blood pressure measurement, and anemia assessment). The category “other ANC” encompassed outcomes such as depression, anxiety and stress, physical activity, and general health knowledge.

The “delivery care” category covered outcomes such as child delivery at health facilities and emergency obstetric care. The category “other delivery care” covered pregnancy outcomes, such as miscarriage, stillbirth, neonatal mortality, birth weight, birth preparation, childbirth complications, maternal and neonatal malnutrition screening, and neonatal asphyxia.

PNC outcomes included the number of postnatal visits, childhood immunization, breastfeeding practices, and prevention of mother-to-child HIV transmission (PMTCT). The category “other postnatal care” encompassed service utilization during the postnatal period for infectious diseases, neonatal and infant death, postnatal depression, contraception use, diet, physical activity, nutritional status monitoring, and family planning. Types of outcomes assessed by each study are listed in Additional file 3: Table A3. In this article, we report the results of selected outcomes most frequently measured and reported in the reviewed studies, i.e., the number of ANC visits, the delivery rate at health facilities, child immunization rates, and child feeding practices.

#### Effect measures

The included studies varied on essential aspects, such as study design, quality, duration, and settings, as well as mHealth function and outcome specifications, such as the number and place of ANC/PNC visits and the number and type of vaccinations. We used forest plots to compare the effects across articles. After attempting multiple meta-analyses and sensitivity analyses, we found the heterogeneity too high (*I*^2^ > 90) for a meaningful meta-analysis. We, therefore, refrained from synthesizing any pooled effect measures from these studies.

Most articles reflected an odds ratio (OR) as the primary effect, and others reported risk ratios (RR). We calculated a crude risk ratio (cRR) when the primary effect size was not reported, while data on the outcomes in the intervention and control groups were available. We calculated those studies’ crude OR (cOR) for comparison and found less than a 7% difference between OR and RR. Only cRR was included in the review, which has an advantage, especially in the cases of small numbers, that our final estimate would tend to be more conservative. RCTs or cluster RCTs reporting pre- and post-effect measures for intervention and control groups were assumed to be balanced at baseline, given that all the reviewed publications were peer-reviewed. Hence, only post-intervention effect methods were taken into account. When a difference coefficient was reported, we converted it to an OR using an exponential function.

## Results

### Included studies

**Fig. 1:**
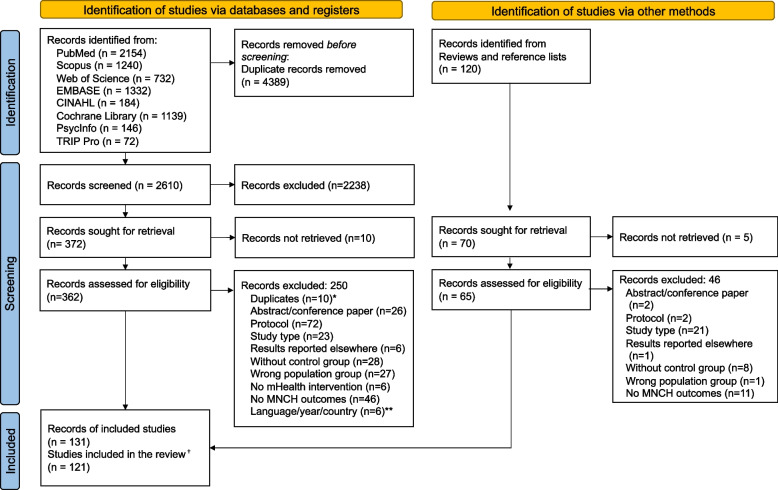
Flow diagram of the study selection process

We identified 7119 articles—6999 through database searches and 120 through published systematic reviews [8, 19–23, 164] and reference lists. Figure 1 illustrates the screening and complete study assessment processes, indicating the number of articles excluded for a given criterion. We included 131 articles based on 121 studies (55 RCTs, 39 cluster RCTs, and 37 quasi-experimental study articles). Geographically, 33 articles were from studies in East Africa (Ethiopia, Kenya, Malawi, Mozambique, Rwanda, Tanzania, Uganda, and Zimbabwe), 16 from North and West Africa (Côte d`Ivoire, Egypt, Ghana, Guinea, Mali, and Nigeria), seven from Central and Southern Africa (Botswana, Cameroon, and South Africa), 25 from South Asia (Bangladesh, India, Nepal, and Pakistan), 15 articles from East Asia (China and Hong Kong), 11 from Southeast Asia (Cambodia, Indonesia, Malaysia, the Philippines, Thailand, and Vietnam), 16 from the Middle East (Iran, Palestine, and Turkey), and seven from South America and South Pacific (Brazil, Ecuador, Guatemala, and Samoa). One multi-country study reported combined findings from India, Mozambique, and Pakistan [153]. The study population comprised pregnant women and children between 0 and 2 years of age and their mothers. For cases of potential data overlap when studies were carried out in the exact geographical location or when publications were derived from the same interventions, all available articles were included as long as the outcomes of interest were relevant to our study objectives.

### Synthesis of results

Additional file 4: Table A4 summarizes the study characteristics, outcomes, mHealth functions and forms, and quality assessment results. Further details of the study intervention designs and resulting outcome effects can be found in Additional file 1: Table A1.

#### Risk of bias

The detailed quality assessment results are available in Additional file 5: Table A5a for RCTs and cluster RCTs and Additional file 5: Table A5b for quasi-experimental studies. Of the 94 articles on RCTs and cluster RCTs, 43 were at low, 39 at moderate, and 12 at high risk of bias. The high risk of bias was primarily due to inappropriate randomization and incomplete data. As for the articles from quasi-experimental studies, out of the nine questions stipulated in the JBI checklist [31], nine scored 9/9, one scored 8/9, and the remaining 27 scored 7/9 or below. We used these scores to categorize the level of risk into three levels: high (9/9), moderate (8/9), and low (7/9 or below). RCT and cluster-RCT articles generally performed well, with 75 (80%) exhibiting a low risk of bias in randomization, 78 (83%) low risk in performance, 71 (76%) low risk of data completeness, 81 (86%) low risk in outcome measurements, and 90 (96%) low risk in reporting. Twenty-six RCT (47%) and 15 cluster-RCT (38%) articles displayed an overall low risk of bias, while eight (15%) RCT and four (10%) cluster-RCT articles displayed an overall high risk of bias. The quality of non-randomized experimental studies was generally compromised due to dissimilarities between comparison groups and the magnitude of missing data.

#### mHealth form and functions

**Fig. 2:**
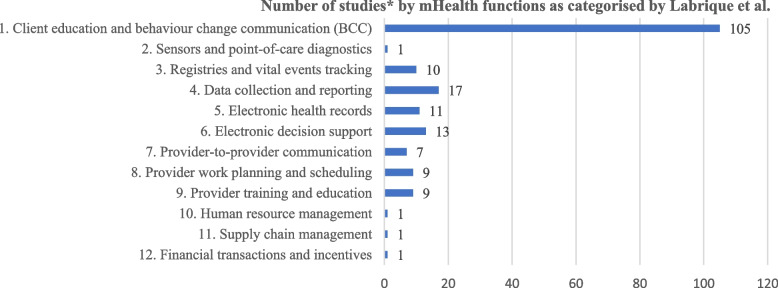
Number of included studies by 12 mHealth functions

Figure 2 shows the number of studies by mHealth functions. Out of 121 studies reviewed, 105 (86.8%) used mHealth Function 1 (client education and BCC), 17 (14.0%) used mHealth Function 4 (data collection and reporting), 13 (10.7%) used mHealth Function 6 (electronic decision support), 11 (9.1%) used mHealth Function 5 (electronic health records), and 10 (8.3%) used mHealth Function 3 (registries and vital events tracking). There was a high expectation of mHealth Function 1, typically used to deliver reminders or information (BCC) for pregnant women and mothers.

Studies used various delivery modes (voice calls, text messaging, transfer of still-moving images, multimedia message services, videos, or audio) of mHealth. Hence, we categorized mHealth forms as either unidirectional, bidirectional, or multi-directional communication between senders and receivers. Most mHealth innovations were designed as unidirectional communication using “push” technology to deliver information or reminders to subscribers’ phones. Messages were often tailored to personal needs, such as information according to gestational age or censored according to HIV status disclosure. Bidirectional communication occurred as short message chats or phone calls between senders and receivers (e.g., nurses and clients) and was commonly employed with unidirectional communication. Data collection and reporting through tablets, phones, and other devices were done using unidirectional or bidirectional communication systems. For example, the two-way communication approach using RapidSMS [130] provided community health workers (CHWs) with a dynamic tool for field data collection and clients’ access to supportive healthcare workers, leading to decentralized decision-making.

We identified three types of interventions with presumably different origins and objectives. The first and most frequent type includes interventions investigating the effectiveness of a single mHealth function, most commonly mHealth Function 1, used as unidirectional communication (e.g., appointment reminders and educational information delivered via text messages to clients). The second type of intervention applied multiple mHealth functions layered on existing parts of a healthcare system, attempting to fill a gap or expand its effectiveness via mHealth interventions. An example of this type is a study conducted in Ethiopia where health extension workers (HEWs) registered women in the intervention groups for their children’s immunization (mHealth Functions 3 and 4). Appointment reminders were sent to the HEWs (mHealth Function 8), who could call health centers for emergency referrals (mHealth Function 7) [40]. The third type of intervention used mHealth components simultaneously at several levels within the health system, combined with other inter-sectoral improvements, such as infrastructure and capacity of human resources. A study by Modi et al. is an example of the latter, where Accredited Social Health Activists (ASHAs) were trained to use Innovative Mobile-phone Technology for Community Health Operations (ImTeCHO), a mobile phone application, to improve the case management of pregnant women within their communities [104]. The latter intervention used nine of the 12 mHealth functions.

### Effects on antenatal care (ANC)

#### ANC attendance

The effect of mHealth interventions on ANC attendance was assessed in 26 studies, including nine RCTs, eight cluster RCTs, and nine quasi-experimental studies. Table 1 shows the individual effect estimates obtained in respective articles or calculated as cRR based on available data for binary outcomes (≥ 3 or < 3, ≥ 4 or < 4, and ≥ 6 or < 6 ANC visits). We did not include studies that did not allow us to calculate effect estimates. Of 26 articles, mHealth Function 1 (client education and BCC) was the most commonly used function among these studies, followed by mHealth 6 (electronic decision support) and Functions 8 (provider work planning and scheduling).

**Table 1:**
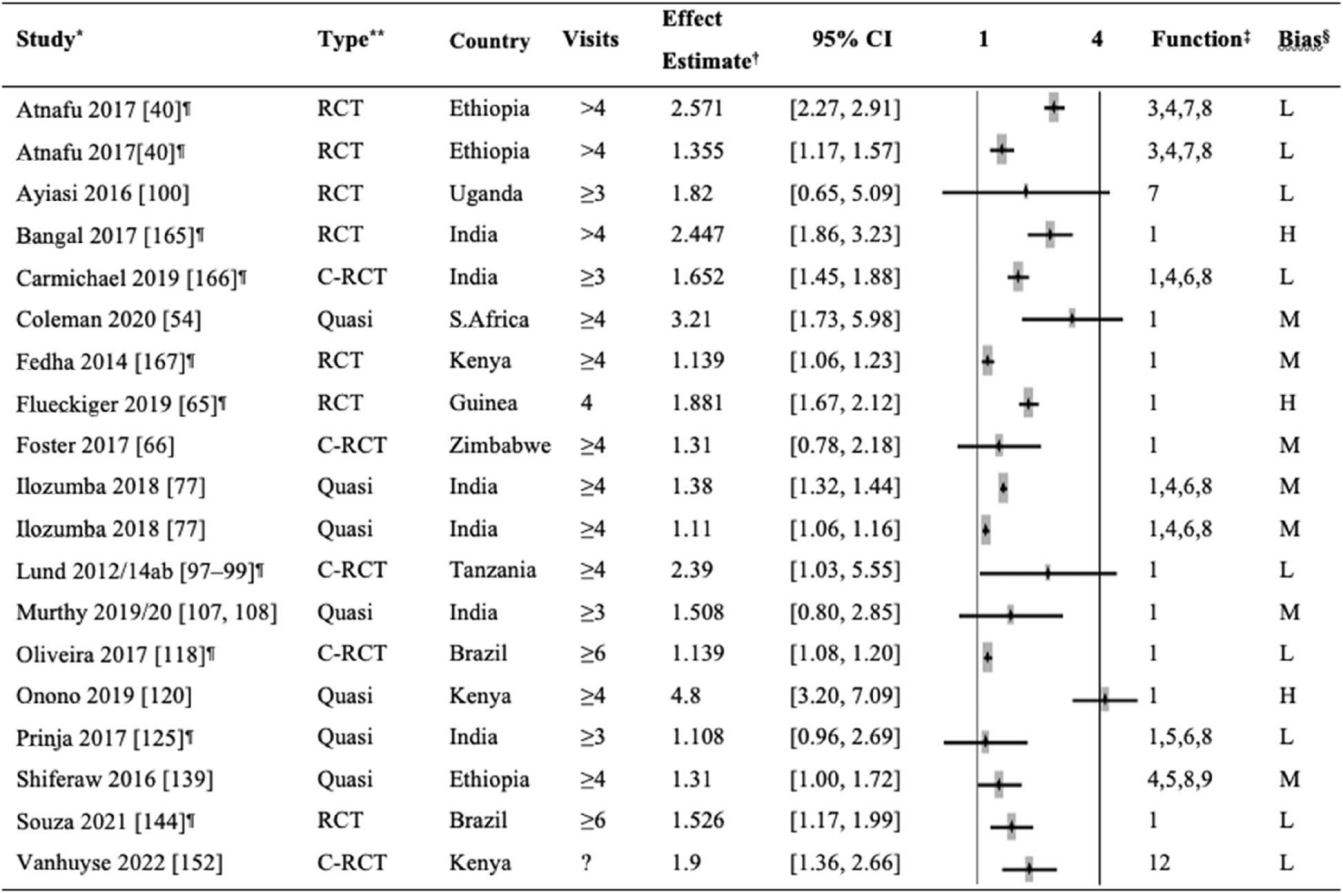
Comparison of antenatal care attendance in the intervention and control groups

^*^Study: The same study in multiple rows indicates multiple intervention groups [40, 42, 50, 54, 62, 65–77, 97–100, 107, 108, 118, 120, 125, 139, 144, 152]

^**^Type of study: *RCT*, randomized controlled trial; *C-RCT*, cluster randomized controlled trial; *Quasi*, quasi-experimental study

^†^Effect estimates: *OR*, odds ratio; *RR*, risk ratio; *cRR*, crude odds ratio

^‡^mHealth functions: 1. Client education and behavior change communication (BCC); 2. Sensors and point-of-care diagnostics; 3. Registries and vital events tracking; 4. Data collection and reporting; 5. Electronic health records; 6. Electronic decision support; 7. Provider-to-provider communication; 8. Provider work planning and scheduling; 9. Provider training and education; 10. Human resource management; 11. Supply chain management; 12. Financial transactions and incentives

^§^The risk of bias was categorized into three levels: high (H), moderate (M), and low (L)

^¶^The studies for which the crude risk ratios (cRR) were calculated by the authors of this systematic review

Regarding effectiveness, seven studies [40, 42, 50, 54, 65, 120, 152] showed robust effect estimates, providing evidence that mHealth interventions could increase the percentage of women receiving at least four ANC visits as recommended by the World Health Organization (WHO) for low-income countries [165]. In a study in South Africa, women in the intervention group who received text messages (mHealth Function 1) were more likely to attend at least four ANC visits than the routine care group [54]. In rural Ethiopia, healthcare workers serving the intervention groups had access to provider work planning and scheduling tools (mHealth Function 8) and received text message reminders to conduct ANC home visits. The results showed a 15%-point increase in ANC attendance from baseline to post-intervention, significantly higher than the control group [40].

Five studies showed higher rates of ANC visits in the mHealth intervention groups compared to the routine care groups [62, 77, 97–99, 118, 144]. However, many other studies found only a borderline significance. Studies in India [42], Guinea [65], and Kenya [120] suggested the effectiveness of their interventions using mHealth Function 1 in women attending at least four ANC visits. However, the risk of bias in these studies was high. Five studies found no significant effect of mHealth interventions on ANC attendance [66, 100, 107, 108, 125, 139]. Seven articles presented results on ANC attendance in varying outcome formats and were not included in Table 1. Of these seven articles, four studies did not assess the number of ANC attendance in isolation [95, 122, 131, 156]. Both Li et al. and Sabin et al. reported composite outcomes, including ANC attendance, while Xie et al. and Paratmanitya et al. focused on the timing of the first ANC visit. The three remaining studies did not find a significant effect of mHealth interventions on their ANC outcomes [84, 130, 154]. An additional article by Coleman et al. [53] underwent full review; nevertheless, it was not included in Table 1 due to potential data overlap with a more recent article published by the same authors [54].

### Effects on delivery care

#### Facility delivery

The effect of mHealth interventions on place of delivery was assessed in six RCTs, 11 cluster RCTs, and five quasi-experimental studies. Table 2 displays the individual effect estimates obtained in individual articles or calculated as a cRR based on available data on the number of events in each group. mHealth Function 1 (education and BCC) was most commonly used (*n* = 12, 60%) [66, 74, 77, 83, 98, 99, 108, 125, 131, 151, 42, 50, 62] either as a sole function or one of the multiple functions employed in the intervention. mHealth Function 4 (data collection and reporting) was also commonly used (*n* = 9, 45%) [40, 44, 50, 74, 77, 126, 135, 139, 153], followed by mHealth Function 6 (electronic decision support, *n* = 8, 40%) [44, 50, 74, 77, 125, 126, 135, 153], mHealth Function 8 (provider work planning and scheduling, *n* = 6, 30%) [40, 50, 74, 77, 125, 139], mHealth Function 5 (electronic health records, *n* = 6, 30%) [44, 125, 126, 135, 139, 153], and mHealth Function 3 (registries and vital events tracking, *n* = 5, 25%) [40, 44, 126, 135, 153]. Other functions used by other studies included mHealth Function 7 (provider-to-provider communication, *n* = 2, 10%) [40, 100], mHealth Function 9 (provider training and education, *n* = 2, 10%) [83, 139], and mHealth Function 12 (financial transactions and incentives, *n* = 1, 5%) [152].

**Table 2:**
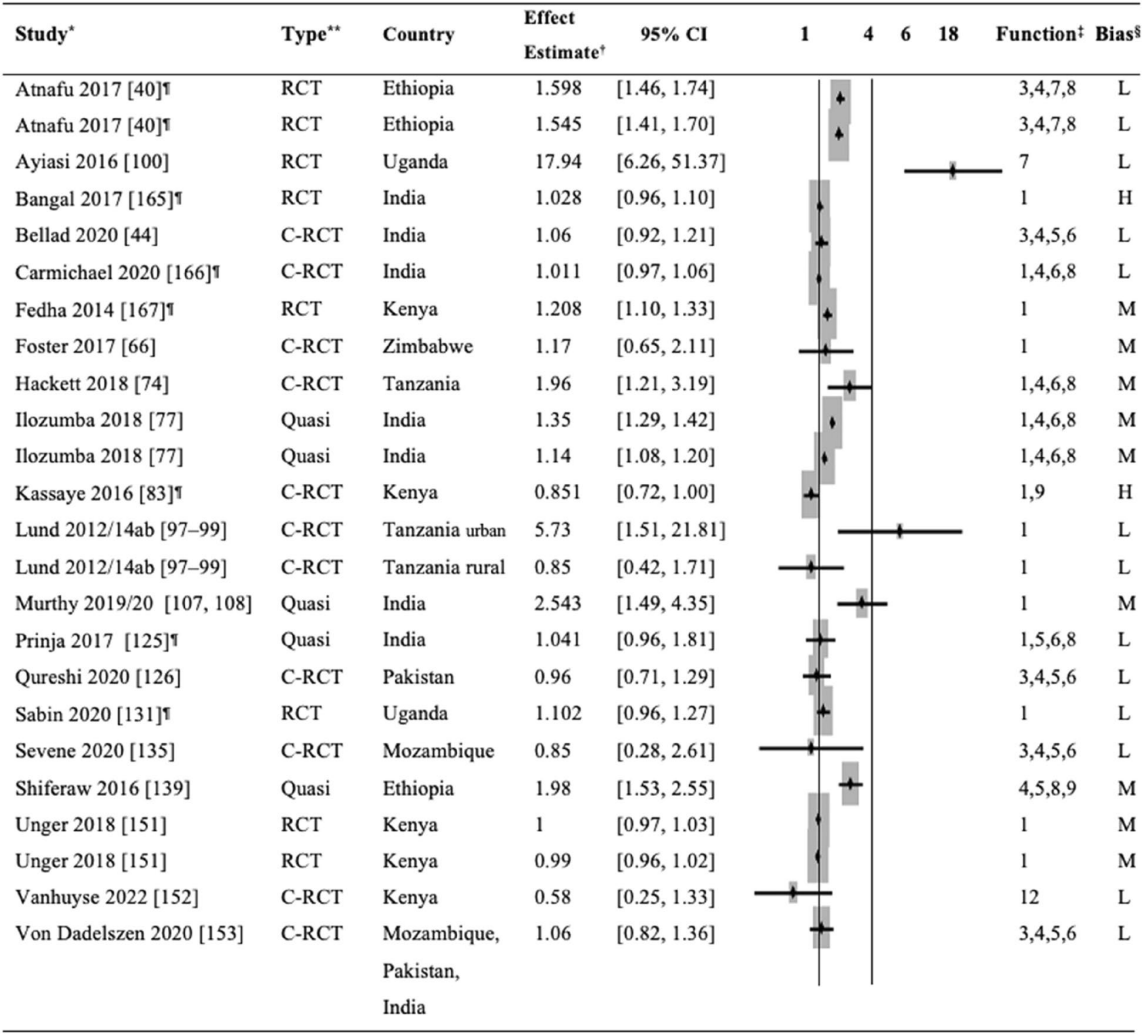
Comparison of facility delivery in the intervention and control groups

^*^Study: The same study shown in multiple rows indicates multiple intervention groups in the study [40, 42, 44, 50, 62, 66, 74, 77, 83, 97–100, 107, 108, 125, 126, 131, 135, 139, 151–153]

^**^Type of study: *RCT*, randomized controlled trial; *C-RCT*, cluster randomized controlled trial; *Quasi*, quasi-experimental study

^†^Effect estimates: *OR*, odds ratio; *RR*, risk ratio; *cRR*, crude odds ratio

^‡^mHealth functions: 1. Client education and behavior change communication (BCC); 2. Sensors and point-of-care diagnostics; 3. Registries and vital events tracking; 4. Data collection and reporting; 5. Electronic health records; 6. Electronic decision support; 7. Provider-to-provider communication; 8. Provider work planning and scheduling; 9. Provider training and education; 10. Human resource management; 11. Supply chain management; 12. Financial transactions and incentives

^§^The risk of bias was categorized into three levels: high (H), moderate (M), and low (L)

^¶^The studies for which the crude risk ratios (cRR) were calculated by the authors of this systematic review

Eight articles included in this review presented the effect of mHealth interventions in increasing deliveries in health facilities, though with varied effect sizes [40, 62, 74, 77, 97–100, 139, 62]. In Uganda, village health teams conducted educational sessions with families on relevant MNCH topics and could call professional health workers (mHealth Function 7) on challenging matters [100]. The study found a significant difference in the proportion of facility delivery between the intervention and routine care groups. Another study in Tanzania equipped CHWs with smartphone-based job aids for data collection, decision-making support, and home-visit scheduling functions (mHealth Functions 4, 6, and 8). The CHWs were prompted to counsel pregnant women on the importance of the delivery place (mHealth Function 1). The proportion of women giving birth in a facility was significantly higher in the intervention than in the control group [74]. In a study in India, female frontline workers received mobile phone tools for scheduling reminders for ANC home visits. The proportion of women delivering in a health facility increased significantly in the intervention group relative to the control and the quasi-control groups [77]. In Kenya, ANC appointment reminders were sent to pregnant women directly with relevant educational information (mHealth Function 1) via text messages and phone calls. The study found a significant increase in facility delivery rates in the intervention group [62].

Two other articles from Rwanda and Nigeria found improvement in facility delivery [119, 130]. However, we did not include them in Table 2 as the outcome format did not allow us to derive a comparable effect estimate. The remaining 12 studies did not find a significant increase in facility delivery rates attributable to the respective mHealth intervention [42, 44, 50, 66, 83, 125, 126, 131, 135, 151–153].

### Effect on postnatal care (PNC)

For PNC outcomes, we report findings on the most frequently measured outcomes among the reviewed articles—child immunization rates, exclusive breastfeeding, and early breastfeeding initiation.

#### Child immunization

Twelve articles assessed childhood immunization coverage per national guidelines until approximately 12 months of age [49, 54, 55, 58, 50, 66–68, 71, 84, 107, 108, 149, 152], a combination of vaccinations for a shorter or longer duration [40, 43, 56, 85, 109], including boosters [125, 134]. Nine RCTs, six cluster RCTs, and six quasi-experimental studies assessed the effect of mHealth interventions on childhood immunization. Table 3 displays the individual effect estimates obtained in individual articles or calculated as a cRR based on the available data on the number of events in each group.

**Table 3:**
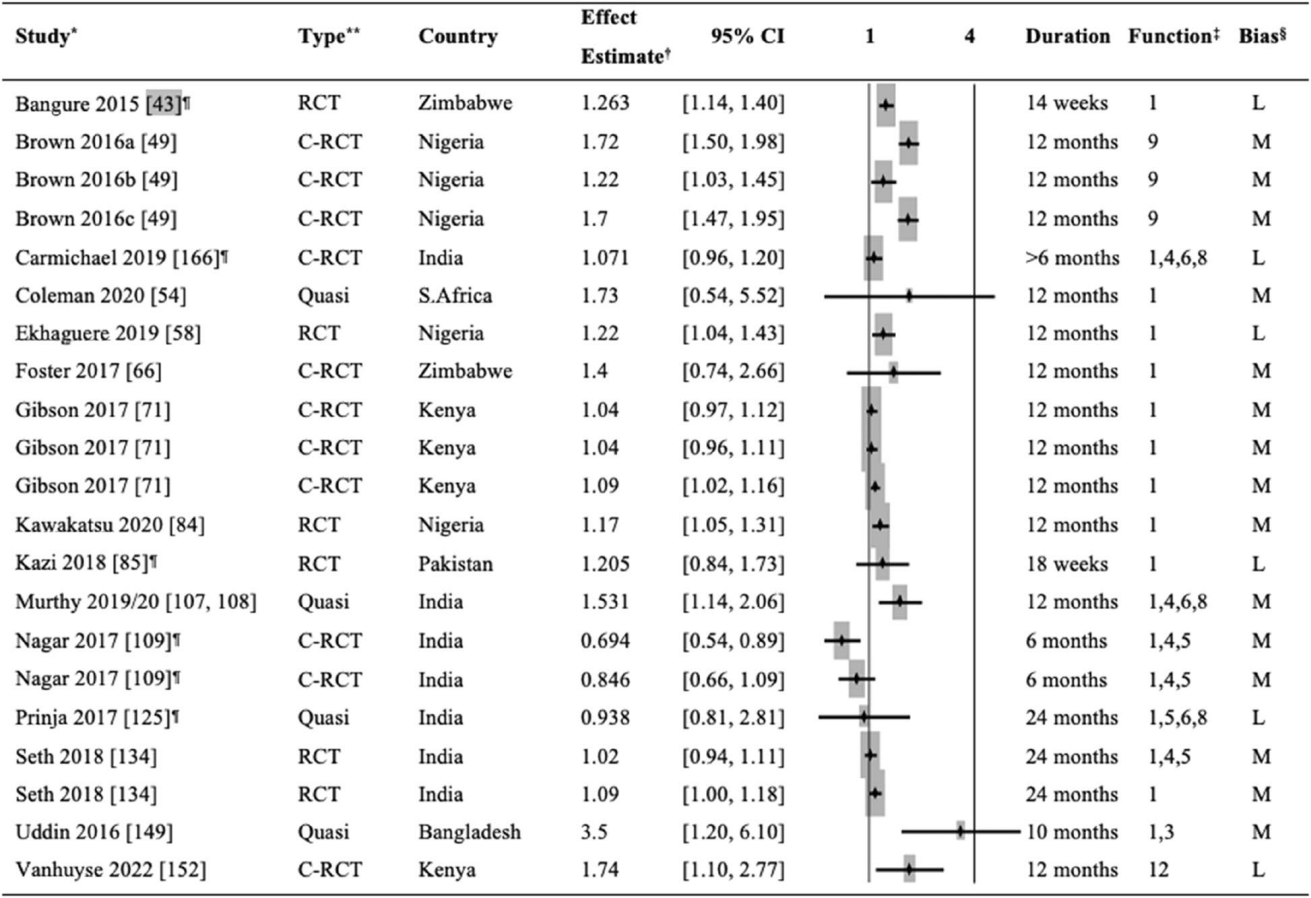
Comparison of childhood immunization in the intervention and control groups

^*^Study: The same study in multiple rows indicates multiple intervention groups [43, 49, 50, 54, 58, 66, 71, 84, 85, 107–109, 125, 134, 149, 152]

^**^Type of study: *RCT*, randomized controlled trial; *C-RCT*, cluster randomized controlled trial; *Quasi*, quasi-experimental study

^†^Effect estimates: *OR*, odds ratio; *RR*, risk ratio; *cRR*, crude odds ratio

^‡^mHealth functions: 1. Client education and behavior change communication (BCC); 2. Sensors and point-of-care diagnostics; 3. Registries and vital events tracking; 4. Data collection and reporting; 5. Electronic health records; 6. Electronic decision support; 7. Provider-to-provider communication; 8. Provider work planning and scheduling; 9. Provider training and education; 10. Human resource. management; 11. Supply chain management; 12. Financial transactions and incentives

^§^The risk of bias was categorized into three levels: high (H), moderate (M), and low (L)

^¶^The studies for which the crude risk ratios (cRR) were calculated by the authors of this systematic review

As with the studies assessing other outcomes, mHealth Function 1 (education and BCC) was the most commonly used (*n* = 13/15) as a sole function or one of the multiple functions employed in the interventions. Two studies used other functions, such as financial transactions and incentives (mHealth Function 12), and one study used electronic health records (mHealth Function 5), electronic decision support (mHealth Function 6), and provider work planning and scheduling (mHealth Function 8).

As for the outcome effects, seven articles found that mHealth intervention improved immunization rates [43, 49, 58, 84, 107, 108, 149, 152]. For example, a study in Zimbabwe sent text message reminders (mHealth Function 1) to women in the intervention group before the 6th, 10th, and 14th week vaccination appointments resulting in a significant increase in immunization coverage among the intervention group at 6 weeks (96.7% vs. 82.2%, *p* < 0.001), 10 weeks (96.1% vs. 80.3%, *p* < 0.001), and 14 weeks (94.7% vs. 75.0%, *p* < 0.001) compared to the control group. Furthermore, the controls had a more significant delay in vaccination [43]. Three studies in Nigeria sent reminders to women using text messages, emails, or voice recordings (mHealth Function1) and increased immunization rates in intervention groups [49, 58, 84]. Similar findings were observed in studies in India [107, 108] and Bangladesh [149]. In Kenya, women received conditional cash transfers (mHealth Function 12) equivalent to US$4.5 per visit to health facilities for ANC, delivery, PNC, and childhood immunization. A modest increase in childhood immunization appointments was reported [152].

However, eight studies did not find significant effects of mHealth interventions on immunization [50, 54, 66, 71, 85, 109, 125, 134]. We did not include six studies [40, 55, 56, 67, 68, 116] in Table 3 because the outcomes reported did not allow us to extract or calculate effect estimates. Among these studies, the results were contradictory, with two studies showing significant mHealth intervention effects on immunization rates Field [75, 82], while four had no significant impact.

#### Feeding practices

Table 4 shows the outcomes of exclusive breastfeeding reported in 17 papers [34, 39, 46, 47, 50, 63, 64, 67, 68, 78–80, 86, 91, 102, 112, 140, 146, 151, 155]. Six of these studies additionally assessed the effect of mHealth on early breastfeeding initiation within one-hour post-delivery [34, 46, 64, 140, 155, 50].

**Table 4:**
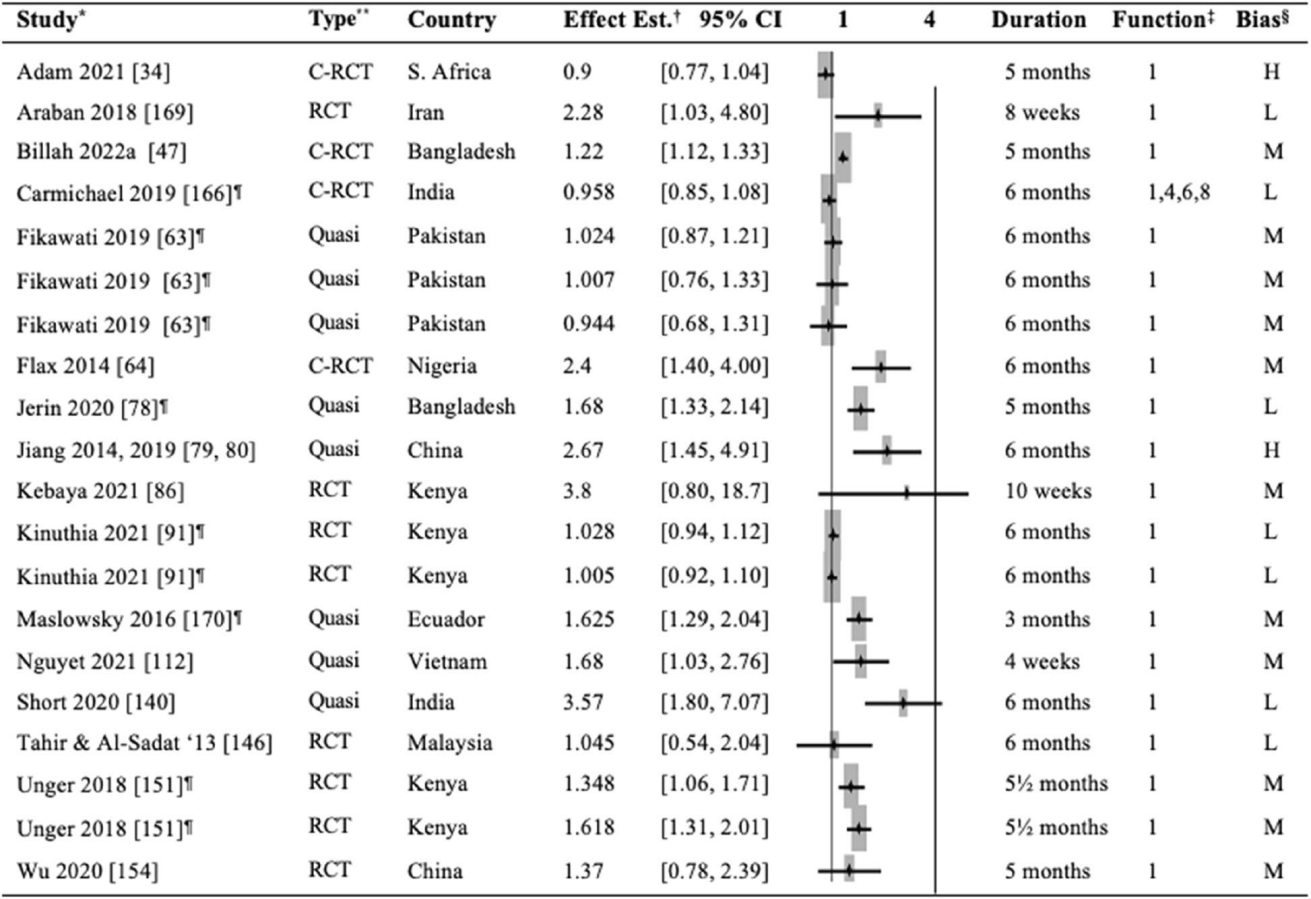
Comparison of exclusive breastfeeding in the intervention and control groups

^*^Study: The same study in multiple rows indicates multiple intervention groups [34, 39, 47, 50, 63, 64, 78–80, 86, 91, 102, 112, 140, 146, 151, 154]

^**^Type of study: *RCT*, randomized controlled trial; *C-RCT*, cluster randomized controlled trial; *Quasi*, quasi-experimental study

^†^Effect estimates: OR, odds ratio; RR, risk ratio; cRR, crude odds ratio

^‡^mHealth functions: 1. Client education and behavior change communication (BCC); 2. Sensors and point-of-care diagnostics; 3. Registries and vital events tracking; 4. Data collection and reporting; 5. Electronic health records; 6. Electronic decision support; 7. Provider-to-provider communication; 8. Provider work planning and scheduling; 9. Provider training and education; 10. Human resource management; 11. Supply chain management; 12. Financial transactions and incentives

^§^The risk of bias was categorized into three levels: high (H), moderate (M), and low (L)

^¶^The studies for which the crude risk ratios (cRR) were calculated by the authors of this systematic review

We reviewed seven articles on early breastfeeding initiation, as shown in Table 5, including a study from India [107, 108]. Some studies also assessed the effect on colostrum feeding [46, 47, 64, 107, 108, 140], pre-lacteal feeding [46, 47, 140, 155], complementary feeding, supplementary feeding, bottle feeding, formula feeding, and breastfeeding awareness [34, 39, 61, 100, 102, 124, 135, 140].

**Table 5:**
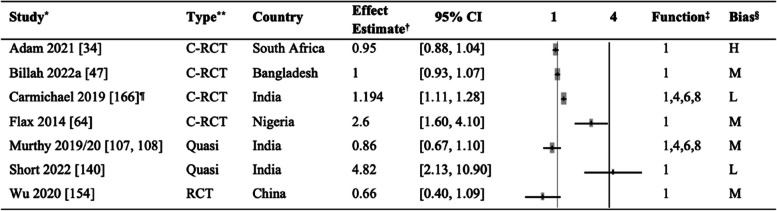
Comparison of early initiation in the intervention and control groups

^*^Study: The same study in multiple rows indicates multiple intervention groups [34, 47, 50, 64, 107, 108, 140, 154]

^**^Type of study: *RCT*, randomized controlled trial; *C-RCT*, cluster randomized controlled trial; *Quasi*, quasi-experimental study

^†^Effect estimates: *OR*, odds ratio; *RR*, risk ratio; *cRR*, crude odds ratio

^‡^mHealth functions: 1. Client education and behavior change communication (BCC); 2. Sensors and point-of-care diagnostics; 3. Registries and vital events tracking; 4. Data collection and reporting; 5. Electronic health records; 6. Electronic decision support; 7. Provider-to-provider communication; 8. Provider work planning and scheduling; 9. Provider training and education; 10. Human resource management; 11. Supply chain management; 12. Financial transactions and incentives

^§^The risk of bias was categorized into three levels: high (H), moderate (M), and low (L)

^¶^The studies for which the crude risk ratios (cRR) were calculated by the authors of this systematic review

In terms of mHealth functions, all 18 articles on exclusive breastfeeding and early breastfeeding initiation used mHealth Function 1 (education and BCC). A study in India additionally used mHealth Function 4 (data collection and reporting), mHealth Function 6 (electronic decision support), and mHealth Function 8 (provider work planning and scheduling) [50, 107, 108].

Results of the effectiveness of mHealth interventions on exclusive breastfeeding and early breastfeeding initiation were mixed. Nine studies [59, 79-86, 89-] found moderate to higher exclusive breastfeeding rates attributable to mHealth interventions, of which two [64, 140] further demonstrated their effectiveness on early breastfeeding initiation. Examples of effective exclusive breastfeeding interventions include an RCT study in Iran in which pregnant women in the intervention group received breastfeeding self-efficacy education sessions, information booklets, and biweekly text messages (mHealth Function 1). The exclusive breastfeeding rates differed significantly between the intervention and control groups at 8 weeks postpartum [39]. In a study in Bangladesh [78], nurses underwent training on infant and young child feeding. They subsequently provided women in the intervention group with tailor-made support on breastfeeding, contacted them biweekly, and had a lactation consultant available as needed. The exclusive breastfeeding rate was significantly higher among the intervention than the control group.

Studies reporting effectiveness in exclusive breastfeeding and early breastfeeding initiation include a cluster RCT in Nigeria, where pregnant women were provided with breastfeeding learning sessions and educational text messages (mHealth Function 1), together with songs and dramas conveying the information and messages. The study found significantly higher rates of exclusive breastfeeding at six months and early breastfeeding initiation in the intervention group than in the routine care group [64]. A similar study in India demonstrated strong effects of the mHealth intervention on prolonging exclusive breastfeeding and early breastfeeding initiation compared to a control group receiving routine care [140]. However, a study in India [50] in which ASHAs were equipped with a mobile application to provide health information, guidelines, and checklists (mHealth Function 6), patient tracking and data collection (mHealth Function 4), and automated scheduling tools (mHealth Function 8) found no evidence of improved exclusive breastfeeding six months postpartum. However, the effect on early breastfeeding initiation was statistically significant. Seven studies found no significant impact of mHealth interventions on exclusive breastfeeding rates [34, 50, 63, 86, 91, 146, 151, 155]. We did not include a study in Malawi [67, 68] in Table 4 because the reported outcome did not allow us to extract an effect estimate.

## Discussion

Overall, this systematic review suggests that mHealth interventions targeting MNCH may increase attendance in ANC. However, the high heterogeneity between studies and the limited reporting quality prohibited calculating a pooled estimate. mHealth interventions can be considered adequate for improving vaccination timeliness for those who attend their appointments. However, the effects of mHealth on facility-based deliveries or child immunization attendance were inconsistent. The synthesized evidence suggests the positive impact of mHealth reminders and information provision on ANC and PNC attendance, although the effects were moderate [22, 166–169]. A review by Colaci et al. found that text messages enhanced the acceptability of maternal care among pregnant women, including skilled birth attendance [168]. Another meta-analysis of studies from 11 LMICs by Eze et al. suggests that SMS reminders can contribute to achieving high and timely childhood immunization coverage [170]. Concerning the feeding practice, the effects of mHealth were inconsistent, which may reflect a complex interplay of barriers in promoting exclusive breastfeeding [171]. However, improving awareness among pregnant women and mothers and performing regular follow-ups are crucial to addressing low breastfeeding rates [172–174], and the significant role the mHealth may play is envisaged.

Besides the study quality, the inconsistent results in this review may be due to the complex interaction of a plethora of determinants that mHealth cannot fully address. The factors may include sociocultural beliefs, economic and physical accessibility, knowledge and perception of benefits and needs, and service quality [175]. The mHealth behavior change interventions must be designed based on theoretically validated mechanisms and guided by formative research of the specific target populations and their behavioral determinants [176, 177]. In the LMIC context, the gap mHealth can fill is often not the only missing link to improve the MNCH [178]. For example, nudging women with information and reminders may not necessarily result in women delivering at facilities or improving feeding practices, as these behaviors are highly affected by socio-economic, environmental, cultural, and health system factors [175, 179]. In this context, evaluating mHealth interventions implemented with high fidelity may provide an opportunity to identify further gaps in health programming.

In terms of mHealth functions, we observed that all 12 functions of mHealth described by Labrique et al. [163] was used in the reviewed articles. The most frequently used function among the reviewed studies by far was “client education and BCC” (mHealth Function 1), as seen in past reviews [22, 164, 167], providing relevant information and reminders for ANC/PNC appointments, childbirth, immunization, and breastfeeding, which had the advantage of simplicity, feasibility, and achievability.

mHealth functions as direct support for health workers (mHealth Functions 6–9) were employed in 7–13% of the studies. These mHealth interventions may have had an indirect impact on the health outcomes of the beneficiaries. However, these functions were often used alongside other functions that directly targeted the beneficiaries, and the effect attributable to each function was not measured independently. The potentially powerful sensors and point-of-care diagnostics, human resource management, supply chain management, and financial transaction (mHealth Functions 2, 10, 11, and 12) were not commonly used in the reviewed studies, reflecting a possible limitation of our search strategy or a genuine scarcity of such interventions in the area of MNCH in LMICs.

Concerning quality, our analysis found that several factors may account for the absence of definitive results in this review: (1) moderate or high risk of bias among the more significant proportion of studies (62%); (2) lack of power due to small sample size (a characteristic of pilot studies), high rates of loss to follow-up, and the multitude of outcomes reported by each study (especially for educational interventions); (3) data reliability of self-reported outcomes (such as with infant feeding practices); and (4) circumstantial challenges such as technological failures, staff turnover, and relocation of participants. Studies have pointed out that mHealth studies are typically under-theorized, poorly specified, and vaguely described, and as a result, lack the specific rigor required for experimental studies [8, 180, 181]. We found that the articles in this review commonly would have benefitted from more detailed descriptions of randomization processes, allocation concealment, and blinding, without which the validity of the methodology could not be established. Referencing the evaluation guidelines for reporting evidence of mHealth interventions, such as the Mobile Application Rating Scale (MARS) [182] and WHO’s mHealth Evidence Reporting and Assessment (mERA) checklist, in addition to standard guidance on trials such as Consolidated Standards of Reporting Trials (CONSORT) [183, 184] is strongly recommended for future studies.

Our review further exposed the critical need to consider the digital infrastructure and technical capacity in LMIC settings, which can often be the significant barriers to flourishing mHealth implementation [180, 185]. There persist apparent age and sex, not to mention urban–rural gaps in access to mobile communication technology, especially in LMICs [186]. In the reviewed studies, mobile phone ownership was often a prerequisite to participation in mHealth programs, and some of the participating women relied on shared devices with partners or families. When devices are shared, client confidentiality and autonomy can be compromised. Mobile phone ownership, literacy, rural and urban residency, and socio-economic status could risk further marginalizing vulnerable groups [21, 187, 188]. For the HCPs and CHWs, health systems and the workforce often lack the capacity to manage data and digital technology [189], and the introduction of mHealth tools could burden the users [190]. At the same time, mHealth is often considered to promote their empowerment, autonomy, and improved incentives. Implementation science research to explore usability, feasibility, and acceptability in the specific context is strongly recommended as part of RCTs to enhance the adoption and informativeness of the overall trial interventions [191, 192]. Coupled with high-quality evidence with large-scale and more rigorous RCT designs to establish the validity and cost-effectiveness of mHealth interventions, accumulating such evidence will guide the replication and scaling-up of effective intervention models while enabling optimal allocation of limited resources in the LMICs.

This systematic review focused exclusively on experimental and quasi-experimental studies at the risk of neglecting the complete picture of the currently available evidence. This selection was to ensure the quality of the review by excluding observational studies, which lack internal (i.e., methodological strength) and external (i.e., generalizability) validity. We limited our search to English-language papers published in peer-reviewed journals, which may have resulted in the omission of informative articles on trials, including those conducted by organizations outside conventional academia. By focusing on LMICs, we excluded the studies in high-income countries, including studies investigating mHealth use in disadvantaged or marginalized populations in those countries, who may have had much in common with residents of LMICs. Finally, we acknowledge the time lapse between the initial search and the completion of the analysis. The comprehensive analyses necessitated more than 12 months to complete, involving meticulous review of a significant number of included studies. This extensive process ensured accurate comparison of effect measures across heterogeneous studies, precise categorization, thorough quality assessment, and comprehensive descriptive reporting.

## Conclusions

Our review demonstrated that mHealth interventions could be a practical approach to increase ANC attendance and improve the timeliness of child immunization. However, their effects on facility-based deliveries, child immunization coverage, and breastfeeding practices were inconclusive. Nonetheless, mHealth’s potential to fill the longstanding gaps in BCC and data collection in resource-limited LMICs is unquestionable. However, while the number of mHealth studies in LMICs has been proliferating, weak and inconsistent evidence continues to plague the field, thus preventing us from drawing robust conclusions. Further quantitative research with high rigor to assess the effectiveness of mHealth and implementation research to explore the context-specific facilitators and intervention barriers are highly warranted.

### Supplementary Information

Additional file 1: Table A1 Search strategy

Additional file 2: Table A2 Descriptive review results

Additional file 3: Table A3 Outcomes by health domains

Additional file 4: Tables A4a and A4b Descriptive review and results

Additional file 5: Table A5a and A5b Quality assessment results

## Data Availability

No additional data are available.

## Abbreviations

ANC: Antenatal care
ASHA: Accredited Social Health Activist
BCC: Behavior change communication
CHW: Community health worker
CINAHL: Cumulative Index to Nursing & Allied Health
cOR: Crude odds ratio
cRR: Crude risk ratio
CONSORT: Consolidated Standards of Reporting Trials
HCP: Health care provider
HEW: Health extension worker
ImTeCHO: Innovative Mobile-phone Technology for Community Health Operations
JBI: Joanne Briggs Institute
LMIC: Low- and middle-income country
MARS: Mobile Application Rating Scale
MeSH: Medical subject heading
MNCH: Maternal, newborn, and child health
mERA: MHealth Evidence Reporting and Assessment
mHealth: Mobile health
OR: Odds ratio
PMTCT: Prevention of mother-to-child transmission
PNC: Postnatal care
PRISMA: Preferred Reporting Items for Systematic Reviews and Meta-Analyses
PROSPERO: International Prospective Register for Systematic Reviews
RCT: Randomized controlled trial
RR: Risk ratio
SMS: Short message service
WHO: World Health Organization
