# Supplementary material for: Impact of mHealth interventions on maternal, newborn, and child health from conception to 24 months postpartum in low- and middle-income countries: a systematic review
Source: BMC Med. 2024 May 15;22:196. doi: 10.1186/s12916-024-03417-9 (PMC11095039; doi:10.1186/s12916-024-03417-9)
Supplement: Supplementary file 1 — Additional file 1: Table A1 Search strategy [file 12916_2024_3417_MOESM1_ESM.docx]

**Table A1** Search strategy

PubMed search syntax was converted to comply with other search engines (Embase, The Cochrane Library, PsycInfo, Scopus, Web of Science, CINAHL) using a Polyglot Search Translator for database platforms available [Clark JM, et al. *Improving the translation of search strategies using the Polyglot Search Translator: a randomized controlled trial*. J Med Libr Assoc. 2020;108(2):195–207. doi:10.5195/jmla.2020.834] <https://sr-accelerator.com/#/polyglot> All searches were conducted on the 24.05.22

#1 AND (#2 OR #3 OR #4 OR #5 OR #6 OR #7 OR #8 OR #9 OR #10 OR #11) AND #12

|  |  | Topics | MeSH Terms | Title / Abstract |
| --- | --- | --- | --- | --- |
| 1 | **Mobile Health** | | Telemedicine,  Mobile applications,  Cell Phone | Telemedicine, Telecare, Mobile health, mHealth, m-Health, e-Health, eHealth, Telehealth, mCare, m-care, Mobile Application, Mobile Applications, Mobile Technology, App, Apps, Cell Phone, Smart-phone, Smartphone, Mobile Phone, Mobile Tablet, Text Messaging, Text Message, SMS, Short Message, Multimedia Message, Mobile Communication |
| 2 | **Community** | | | |
|  | **2.1** | **Community Workers** | Community health workers | Community Health Worker, Community Health Workers, CHW, CHWs, Community Health Aide, Community Health Aides, Family Planning Personnel, Village Health Worker, Village Health Workers, Barefoot Doctor, Barefoot Doctors, Village Health Worker, Village Health Workers, Village Health Support, Village Health Support Group, Village Health Support Groups, Out-reach Worker, Out-reach Workers, Front-line Health Worker, Front-line Health Workers, FHWs |
|  | **2.2** | **Rural Communities** | Rural Health Services,  Rural Health,  Rural Population | Rural Health Service, Rural Health Services, Rural Health Center, Rural Health Centers, Rural Service Coverage, Rural Service Access, Rural Service Accessibility, Long Distance Health Care, Rural Health, Rural Population, Rural Populations, Rural Community, Rural Communities, Rural Area, Rural Areas, Difficult-to-reach Area, Difficult-to-reach Areas, Remote Area, Remote Areas |
| 3 | **Midwifery** | | Midwifery | Midwifery, Midwives, Midwife, Traditional Midwife, Traditional Midwives, Birth Attendant, Traditional birth Attendant, Birth Attendance, Traditional Birth Attendance, Skilled Birth Attendant, SBA, Skilled Birth Care |
| 4 | **Pregnancy and Delivery** | | Pregnant women,  Pregnancy,  Delivery, Obstetric | Pregnant Women, Pregnancy, Pregnancies, Gestation, Gravidity, Obstetric Labor, Obstetric Labour, Parturition, Parturitions, labor, Labour, Birth, Births, Childbirth, Childbirths, Birth Settings, Home Birth, Home Childbirth, Traditional Birth, Facility Birth, Facility Births, Facility Childbirth, Facility Delivery, Facility Deliveries, Institutional Birth, Institutional Births, Institutional Delivery, Institutional Deliveries, Hospital Birth, Hospital Births, Hospital Delivery, Hospital Deliveries, Childbirth Facility, Childbirth Facilities, Birth Center, Health Facility Childbirth Service, Child Birth Services, Hygienic Birth, Natural Birth, Natural Childbirth, Pregnancy Outcome, Abortion, Spontaneous Abortion, Live birth, Stillbirth, Obstetric Delivery, Obstetric Deliveries, Caesarean Section, Cesarean Sections, C-section, C Section, C-Sections, Vaginal Birth, Umbilical Cord Clamping, Delayed Cord Clamping, Cord Clamping, Immediate Cord Clamping, Placental Transfusion |
| 5 | **Reproductive and Maternal Health** | | | |
|  | **5.1** | **Reproductive and Maternal Health** | Reproductive Health,  Maternal Health, Maternal Welfare, Reproductive Health Services,  Maternal-child Health Centers,  Postpartum Period, | Reproductive Health, Maternal Health, Maternal Welfare, Reproductive Health Services, Reproductive Health Service, Maternal-child Health Centers, Maternal-child Health Center, RMNCH, Reproductive Maternal Newborn and Child Health Care, Maternal Health Services, Maternal Health Service, Maternal Health Service Utilisation, Maternal Health Service Utilization, Maternal Health Service Uptake, Maternal health Service Access, Maternal Health Service Accessibility, Maternal Health Service coverage, Maternal Health Care, Maternal-Child health Service, Maternal-Child health Services, Maternal Child Health Service, Maternal Child Health Services, Maternal-Child Health Service Utilisation, Maternal-Child Health Service Utilization, Maternal-Child Health Service Uptake, Maternal-Child Health Service Access, Maternal-Child Health Service Accessibility, Maternal-Child Health Service Coverage, Maternal Child Health Service Utilisation, Maternal Child Health Service Utilization, Maternal Child Health Service Uptake, Maternal Child Health Service Access, Maternal Child Health Service Accessibility, Maternal Child Health Service Coverage, MCH Service, MCH Service Utilisation, MCH Service Utilization, MCH Service Uptake, MCH Service Access, MCH Service Accessibility, MCH Service Coverage, Perinatal Care, Postnatal Care, Postnatal Visits, PNC, PNC visit, Postnatal Care Services, PNC Services, Preconception Care, Prenatal Care, Intrapartum Care, Antenatal Care, ANC, Antenatal visits, ANC Visit, Antenatal Care Services, ANC Services, Postpartum, Puerperium, Postpartum Period, Postpartum Women, Postpartum Care, Post-partum Care, Puerperal Care |
|  | **5.2** | **Family Planning** | Family planning services, Contraception | Family Planning Services, Family Planning Service, Family Planning, Pregnancy Planning, Planned Pregnancy, Planned Pregnancies, Family Planning Program, Family Planning Programs, Family  Planning Programme, Family Planning Programmes, Contraception, Fertility Control, Birth Control, Contraceptive Method, Contraceptive Methods, Female Contraception, Unplanned Pregnancy, Unintended pregnancy, Unwanted Pregnancy, Abortion |
|  | **5.3** | **Maternal Treatment and Behaviour** | Reproductive Behavior  Insecticide-Treated Bednets  Infectious Disease Transmission, Vertical | Maternal Immunisation, Maternal Immunization, Maternal Vaccination, Anaemia Screening, Anaemia, Maternal Anaemia, Reproductive Behavior, Reproductive Behaviour, Safe Abortion, Post Abortion Care, Insecticide Treated Bed Nets, Insecticide-treated Bednets, Treated Mosquito Net, Treated Mosquito Nets, Malaria during pregnancy, Malaria in Pregnancy, Maternal Malaria, Placental Malaria, Maternal Exposure to Malaria, Antimalarial, Antimalarial drugs in Pregnancy, Maternal HIV, HIV Treatment in Pregnancy, Mothers with HIV Infection, HIV during Pregnancy, Antiretroviral Therapy in Pregnancy, Antiretroviral Use during Pregnancy, Vertical Transmission, Vertical Pathogen Transmission, Vertical Infection Transmission, Vertical Infectious Disease Transmission, Maternal-Fetal Infection Transmission, Maternal Fetal Infection Transmission, Maternal-Foetal Infection Transmission, Maternal Foetal Infection Transmission, Mother-to-Child Transmission, Mother to Child Transmission, Fetomaternal Infection Transmission |
| 6 | **Maternal Mortality** | | Maternal Mortality, Maternal Death | Maternal Mortality, Maternal Mortalities, Maternal Death |
| 7 | **Pregnancy Complications** | | | |
|  | **7.1** | **Maternal Complications** | Pregnancy Complications | Pregnancy Complications, Pregnancy Complication, Adverse Birth Outcomes, Adverse Birth Outcome, Obstetric Labour Complications, Obstetric Labor Complications, Obstructed Labour, Obstructed Labor, Emergency Obstetric Care, Ectopic Pregnancy, Premature Infant, Preterm Infant, Neonatal Prematurity, Premature Birth, Preterm Birth, Postpartum Hemorrhage, Postpartum Haemorrhage, Immediate Postpartum Hemorrhage, Immediate Postpartum Haemorrhage, Delayed Postpartum Hemorrhage, Delayed Postpartum Haemorrhage, Postpartum Bleeding, Infectious Pregnancy Complication, Maternal Infection, Maternal Infections, Prenatal Maternal Infection, Maternal Sepsis, Sepsis in Pregnancy, Sepsis in Pregnancies, Sepsis during Pregnancy, Septic Abortion, Parasitic Pregnancy Complications, Postpartum Sepsis, Puerperal Infection, Sexually Transmitted Disease, Sexually Transmitted Diseases, STD, STI |
|  | **7.2** | **Foetal Complications** | Fetal growth retardation,  Fetal Hypoxia,  Fetal Macrosomia,  Prenatal injuries | Fetal Diseases, Foetal Diseases, Fetal Growth Retardation, Foetal Growth Retardation, Fetal Growth Restriction, Foetal Growth Restriction, Intrauterine Growth Retardation, Intrauterine Growth Restriction, Fetal Hypoxia, Foetal Hypoxia, Fetal Macrosomia, Foetal Macrosomia, Prenatal Injury, Prenatal Injuries, Prenatal Exposed Delayed Effects, Neonatal Infection, Neonatal Infections, Newborn Infection, Newborn Infections, Neural tube Defects |
|  | **7.3** | **Chronic Disease Related** | Diabetes, Gestational,  Hypertension, Pregnancy-Induced,  Obesity, Maternal | Gestational Diabetes, Pregnancy-induced Diabetes, Gestational Diabetes Mellitus, Pregnancy in Diabetics, Pregnancy Induced Hypertension, Gestational Hypertension, Pregnancy Transient Hypertension, Eclampsia, Pre-Eclampsia, Maternal Obesity, Obesity in Pregnancy |
|  | **7.4** | **Maternal Depression** | Depression, Postpartum | Depression, Postpartum, Post-partum Depression, Postpartum Depression, Post Partum Depression, Postnatal Depression, Post-natal Depression, Post Natal Depression, Maternal Depression, Perinatal depression, Antenatal Depression |
| 8 | **Infant and Child Health** | | Infant Health,  Child Health | Infant Health, Baby Health, Newborn Health, Neonatal Health, Child Health, Childrens Health, Children’s Health, Child Well Being, Child Wellbeing, Child Well-being, Infant Survival, Child Survival |
| 9 | **Infant and Child Mortality** | | Infant Mortality,  Infant Death,  Perinatal Death,  Fetal Mortality,  Fetal Death,  Child Mortality | Infant Mortality, Infant Death, Neonatal Mortality, Neonatal Death, Post Neonatal Mortality, Perinatal Mortality, Perinatal Death, Fetal Mortality, Fetal Death, Fetal Resorption, Stillbirth, Fetal Demise, Fetal Viability, Child Mortality, Child Mortalities, Child Death, U5 Mortality, U5 Mortalities, U5 Death, Under 5 Mortality, Under 5 Mortalities, Under 5 Death |
| 10 | **Child Health Services** | | Maternal-Child Health Centers, Child Health Services | Maternal-Child Health Centers, Maternal-Child Health Center, Child Health Service, Child Health Service, Infant Health Service, Infant Health Services, Child Vaccination, Child Immunisation, Child Immunization, Childhood Immunisation Coverage, Immunisation Coverage, Childhood Immunization Coverage, Immunization Coverage,  Neonatal Malaria, Malaria in Newborns, Birth Registration, Vitamin A Supplementation, Neonatal Resuscitation |
| 11 | **Others** | | | |
|  | **11.1** | **Birth Weight** | Birth Weight, Infant,  Low Birth Weight | Birth Weight, Infant Low Birth Weight, Birthweight, Low Birth Weight |
|  | **11.2** | **Feeding** | Breastfeeding, Milk, Human, Lactation,  Colostrum | Breastfeeding, Exclusive Breastfeeding, Early Breastfeeding, Early Breastfeeding Initiation, Human Milk, Breast Milk, Lactation, Colostrum, Continued Breastfeeding, Complementary Feeding |
|  | **11.3** | **Kangaroo-Mother Care** | Kangaroo-Mother care method | Kangaroo-Mother Care Method, Kangaroo Mother Care Method, Kangaroo-Mother Care Methods, Kangaroo Mother Care Methods, Kangaroo-Mother Care, Kangaroo Mother Care, KMC |
|  | **11.4** | **Maternal Nutrition Physiological Phenomena** | Maternal Nutrition Physiological Phenomena | Maternal Nutrition Physiological Phenomena, Maternal Nutrition Physiology, Maternal Nutrition, Nutritional Physiology, Maternal Supplementation, Nutrition during Pregnancy, Prenatal Nutritional Physiological Phenomenon, Prenatal Nutritional Physiology, Pregnancy Nutrition, Iron Supplementation, Folic Acid Supplementation, Folic Acid Fortification, Iron-Folic Supplementation, Supplementation during Pregnancy, Pregnancy Supplementation, Micronutrient Supplementation, Calcium Supplementation, Magnesium Sulphate Supplementation |
|  | **11.5** | **Standard Measures** |  | Composite Coverage Index, Health Care Indicators |
|  | **11.6** | **Prenatal Education** | Prenatal Education | Prenatal Education |
| 12 | **Study Type** | | Cohort studies  Case-Control studies  Controlled Before-After Studies  Interrupted Time Series Analysis  Randomized Controlled Trials as Topic | Cohort study, Cohort studies, concurrent studies, Cohort analysis, Cohort analyses, Incidence Study, Incidence Studies, follow up study, follow up studies, follow-up study, follow-up studies, followup study, followup studies, Longitudinal study, Longitudinal studies, Longitudinal survey, Longitudinal surveys, prospective study, Prospective studies, Retrospective study, Retrospective studies, Case-Control Study, Case-Control Studies, Case-Comparison Study, Case-Comparison Studies, Case Control study, Case Control studies, Nested Case-Control Study, Nested Case-Control Studies, Nested Case Control Study, Nested Case Control Studies, Matched Case-Control Study, Matched Care-Control Studies, Matched Case Control Study, Matched Case Control Studies, Controlled Before After Study, Controlled Before After studies, Controlled Before-After Study, Controlled Before-After studies, CBA Study, CBA Studies, Controlled Before and After Study, Controlled Before and After Studies, Interrupted Time Series Analysis, ITS Studies, Interrupted Time Series, Randomized Controlled Trial, Randomised Controlled Trial, Randomized Controlled Trials, Randomised Controlled Trials, Randomized clinical trial, Randomised clinical trial, Controlled clinical Trial, Controlled clinical Trials, RCT, RCTs, Intention to Treat Analysis, double-blind, single-blind, double-blind method, single-blind method, double-blind design, single-blind design, quasi-experimental study |

**TRIP PRO search strategy:**

**ANY of these words in Document:** Telemedicine, Telecare, Mobile health, mHealth, m-Health, e-Health, eHealth, Telehealth, mCare, m-care, Mobile Application, Mobile Applications, Mobile Technology, App, Apps, Cell Phone, Smart-phone, Smartphone, Mobile Phone, Mobile Tablet, Text Messaging, Text Message, SMS, Short Message, Multimedia Message, Mobile Communication

**Study population (filter):** Women, Woman, child, infant, newborn, neonate
