# Supplementary material for: Impact of mHealth interventions on maternal, newborn, and child health from conception to 24 months postpartum in low- and middle-income countries: a systematic review
Source: BMC Med. 2024 May 15;22:196. doi: 10.1186/s12916-024-03417-9 (PMC11095039; doi:10.1186/s12916-024-03417-9)
Supplement: Supplementary file 2 — Additional file 2: Table A2 Descriptive review results [file 12916_2024_3417_MOESM2_ESM.docx]

**Additional File 2: Table A2** Descriptive review and relevant results of the interventions in the studies (n=131)

| Study | Study characteristics | Intervention | mHealth Form* | mHealth Function^**^ | | Outcomes  ^denotes primary outcomes |
| --- | --- | --- | --- | --- | --- | --- |
| 1. Abbaspoor et al. (2020) Iran [32] | Design: RCT  **Methods**: Socio-demographic data and reproductive data forms collected at baseline. Blood samples, FFQ, and IPAQ-SP completed at baseline and endline. OGTT performed at endline.  **Setting**: 2 HCs in Ahvaz, southwest Iran  **Population**: Pre-diabetic pregnant women, 12-14 weeks of GA  **Size**: Total(pre/post)=100/89  Intervention=50/44, Control=50/45 | Intervention: as control + 2 SMS every other day  Control: Face-to-face education in 4 sessions over 12 weeks according to prenatal visits regarding pregnancy and GDM/diabetes + SMS reminder of upcoming appointments | Unidirectional communication (SMS) | 1 | Educational information and appointment reminders | ^ Physical activity level(MET mean±SD):  Intervention group pre 473.00±498, post 852.42±350, P=0.001.  Control group pre 531.84±260, post 637.12±210, P=0.034.  Intervention group pre 473.00±498, Control group pre 531.84±260, P=0.486.  Intervention group post 852.42±350, Control group post 637.12±210, P=0.001. |
| 2.  Abuogi et al. (2022) Kenya [33] | **Design:** Cluster RCT (the MOTIVATE study)  **Methods:** Data collected from clinic visit forms/registers and Open data electronic database  **Setting:** 24 Ministry of Health facilities in Migori, Kisumu, and Homabay Counties, Southwestern Kenyan  **Population:** Pregnant women living with HIV (PWLWH) recruited during ANC visits  **Size:** Total(pre/post)=1331/1042  Arm2=334/263, Arm3=330/262, Arm4=334/264, Control=333/253 | **Arm2:** Automated text messages sent weekly from enrolment until 12 months postpartum + ability to engage with study nurse via telephone  **Arm3:** Community mentor mothers (cMM) carried out home visits (<4 ANC, <9 PNC) designed to maximise retention in PMTCT.  **Arm4:** Arm2 and Arm3  **Control:** Standard care | Unidirectional communication (SMS)  Bidirectional communication (phone calls) | 1 | Educational information (medication and clinic adherence, promotion of maternal and child healthcare services according to stage of pregnancy and age of infant post-delivery) | **^ Adherence to ART and retention in HIV care 12 months postpartum (ITT):** Arm2 78.7%, Arm3 79.4%, Arm4 79.0%, Control 76%  Arm2 vs control: Adj. RR 1.08 (95% CI 0.97 to 1.21) P=0.141  Arm3 vs control: Adj. RR 1.06 (95% CI 0.96 to 1.17) P=0.248  Arm4 vs control: Adj. RR 1.04 (95% CI 0.94 to 1.15) P=0.460  *Per protocol analysis produced significant results for all arms compared to control.* |
| 3.  Adam et al. (2021) South Africa [34] | **Design:** Cluster RCT  **Methods:** Surveys in person or by phone at baseline, 1(T1) and 5(T2) months postpartum  **Setting:** Under-resourced region of the Western Cape province  **Population:** Mentor mothers and pregnant women between 20th and 35th week of pregnancy  **Size:** Mentor mothers: 84  Pregnant women (T0/T1/T2): 1502/1181/924  Intervention=747/565/423, Control=755/616/501 | **Intervention:** 13 short teaching videos delivered by the mentor mothers as part of their counsel to pregnant women and new mothers.  **Control:** - | Unidirectional communication  (Videos delivered via tablet) | 1 | Educational short videos | **^ Short term EBF(1 month):** Intervention group 65%, Control group 70.6%, Adj. RR 0.99 (95% CI 0.91 to 1.07) P=0.764.  **^ Long term EBF(5 months):** Intervention group 47.3%, Control group 51.7%, Adj. RR 0.90 (95% CI 0.77 to 1.04) P=0.087.  **Early initiation of BF:** Intervention group 70.8%, Control group 74.2%, Adj. RR 0.95 (95% CI 0.88 to 1.04, P=0.273.  **Any BF at 1 month:** Intervention group 86.2%, Control group 89.1%, Adj. RR 0.97 (95% CI 0.92 to 1.02) P=0.201.  **Any BF at 5 months:** Intervention group 79.2%, Control group 79.6%, Adj. RR 0.99 (95% CI 0.92 to 1.07) P=0.886.  **No Bottle feeding at 1 month:** Intervention group 75.6%, Control group 80.2%, Adj. RR 0.94 (95% CI 0.87 to 1.02) P=0.117. **No Bottle feeding at 5 months:** Intervention group 60.8%, Control group 61.9%, Adj. RR 0.98 (95% CI 0.85 to 1.14) P=0.807  **No early introduction of complementary food at 1 month:** Intervention group 82.8%, Control group 86.9%, Adj. RR 0.95 (95% CI 0.91 to 1.00) P=0.069. **No early introduction of complementary food at 5 months:** Intervention group 76.9%, Control group 81.4%, Adj. RR 0.94 (95% CI 0.87 to 1.03) P=0.173.  **Maternal knowledge score at 1 month:** Intervention group 12.41, Control group 11.92, RR 1.04 (95% CI 1.01 to 1.07) P=0.012. **Maternal knowledge score at 5 months:** Intervention group 12.38, Control group 12.05, RR 1.03 (95% CI 1.00 to 1.06) P=0.084. |
|  |  |  |  | 4 | Data collection at site by mentor mothers |  |
| 4.  Akbarian et al. (2017) Iran [35] | **Design:** RCT  **Methods:** Demographic questionnaire and hospitalisation records. Outcome data collected during phone calls conversations.  **Setting:**  **Population:** Mother and premature infant hospitalised in NICU or childcare unit  **Size:** Total=100  Intervention=50, Control=50 | **Intervention:** 3 phone calls a week (week 1-4), 2 phone calls a week (week 5-6) to mother regarding maternal and child follow up care  **Control: -** | Bidirectional communication (phone calls) | 1 | Educational information (massage therapy, bathing, diaper change, BF, supplementary feeding, adequate nutrition, supplementation, body temperature, vaccination, development, infections, etc.) | **Hospital readmission 4 weeks after intervention:**  Intervention group 24%, Control group 52%, P=0.004.  **Hospital readmission 6 weeks after intervention:**  Intervention group 8%, Control group 30%, P=0.005.  **Hospital readmission 12 weeks after intervention:**  Intervention group 0%, Control group 14%, P=0.006. |
| 5.  Aksoy Derya et al. (2020) Turkey [36] | **Design:** Quasi-experimental Study  **Methods:** Personal Information Form, Revised Prenatal Distress Questionnaire (NuPDQ), Pregnancy Related Anxiety Questionnaire-Revised 2 (PRAQ-R2)  **Setting:** Public Hospital, East Turkey  **Population:** Pregnant women applying for ANC education class during 3^rd^ trimester  **Size:** Total(pre)=96,  Intervention=48, Control=48 | **Intervention:** One-week interactive education and consultancy provided by weekly phone calls, daily text messages and a digital education process (information related to pregnancy and birth planning during COVID-19).  **Control:** | Unidirectional communication (text messages)  Bidirectional communication (phone calls) | 1 | Educational information | **Pregnant women’s scores on the prenatal distress and pregnancy-related anxiety scale:**  NuPDQ total Pretest (mean±SD): Intervention group 12.18±6.54, Control group 11.93±5.14, P=0.836  NuPDQ total Posttest (mean±SD): Intervention group 8.75±5.10, Control group 11.50±4.91, P=0.008  PRAQ-R2 total Pretest (mean±SD): Intervention group 29.29±9.96, Control group 29.08±8.46, P=0.912  PRAQ-R2 total Posttest (mean±SD): Intervention group 24.25±4.90, Control group 30.04±8.48, P=0.000 |
| 6.  Amoakoh et al. (2019) Ghana [37] | **Design:** Cluster RCT  **Methods:** Data extracted from district health information management system-2 database – data from public health facilities. Baseline data regarding number of doctors/midwifes was collected from each health facility  **Setting:** 16 districts in the Eastern Region of Ghana,  **Population:** District >=1000/year deliveries, 6 intervention districts and 6 control districts  **Size:** Total(health facilities)=176, Intervention=74, Control=102  Total(deliveries)=65,831  Intervention=31,155, Control=34,676 | **Intervention**: Healthcare workers given access to maternal health emergency protocols via phone calls, text messages, access to the internet, and access to data (USSD)  **Control:** - | Unidirectional communication (SMS)  Bidirectional communication (between healthcare workers) | 1 | Monthly reminders on the availability of protocols | **^ Institutional neonatal mortality:** Intervention group 198/31.155, Control group 150/34,676, Adj. OR 2.09 (1.00 to 4.38) P=0.051. |
|  |  |  |  | 6 | Access to electronic decision-making protocols in various formats |  |
| 7.  Anitasari and Andrajiti (2017) Indonesia [38] | **Design:** Quasi-experimental study with two intervention groups  **Methods:** MMAS-8 questionnaire for compliance assessment and blood sample analysis  **Setting:** Two public health centres  **Population:** Pregnant women between 14 and 32 weeks of gestation  **Size:** Total = 74,  Intervention= 36, Control=38 | **Intervention:** routine care + weekly reminders (iron supplementation) for 1 month after administration of supplements    **Control:** routine care + health education leaflet with iron supplements | Unidirectional communication (text messages) | 1 | Iron supplementation reminders | **^ Adherence to treatment:** Intervention group pre 76.3%, post 71.1, Control group pre 66.7%, post 58.3%.  **Haemoglobin level ≥ 11g/dL:** Intervention group pre 47.22%, post 52.78%, Control group pre 55.26%, post 57.69%.  **Haemoglobin level 10.9-10.0g/dL:** Intervention group pre 36.11%, post 22.22%, Control group pre 23.68%, post 28.95%  No difference found in compliance levels and haemoglobin levels between groups. No difference found in compliance levels and haemoglobin levels before and after interventions within groups, except a slight decrease in compliance observed among women in the leaflet group. |
| 8.  Araban et al. (2018) Iran [39] | **Design:** RCT  **Methods:** Socio-demographic data collected at baseline. Outcome data collected at baseline and 8 weeks postpartum using BSES-SF to assess breastfeeding efficacy.  **Setting:** 4 prenatal clinics in West Ahvaz  **Population:** Pregnant women, 35 to 37 weeks of GA  **Size:** Total(pre/post)=120/110  Intervention=60/56, Control=60/54 | **Intervention:** Routine ANC and PNC + breastfeeding self-efficacy intervention consisting of two 1-h group based breastfeeding education sessions, and information booklet, and biweekly text messages until 8 weeks postpartum  **Control:** Routine ANC and PNC | Unidirectional communication (text messages) | 1 | Educational information (encouragement and reinforcement of EBF) | **^ Breastfeeding efficacy(mean±SD):** Intervention group 62.46±4.22, Control group 50.74±4.88, OR 2.89 (95% CI 1.97 to 4.32)  **EBF 8 weeks postpartum:** Intervention group 57.1%, Control group 38.9%, OR 2.28 (95% CI 1.03 to 4.8) |
| 9.  Atnafu et al. (2017) Ethiopia [40] | **Design:** RCT  **Methods:** Structured questionnaires at baseline and 13 months post intervention + data collected as part of the intervention  **Setting:** Three districts in rural Ethiopia  **Population**: For baseline survey: Women 15-49 years of age with at least one child less than 5 year.  For postpartum survey: Women 15-49 years of age with at least one child less than 1 year  **Size:** Total(pre/post)=3218/3089  Intervention1=1065/1066 Intervention2=1073/946, Control=1080/1077  Total HEW(Health extension workers)=189, Intervention1=48, Intervention2=49, Control=92 | **Intervention1 (partial):** HEW register all mothers for ANC on electronic MRF (Maternal registration form) and receive antenatal care appointment schedule and reminders at week 14, 24, 30, and 36. HEW register all children under 11months of age and receive vaccination appointment reminders for 6, 10, 14 weeks, and 9 months. + emergency referral call to HC.  **Intervention2 (full):** As Intervention1 + actively identifying pregnant women who did not register + arrange for mothers who need vaccination. + Call HEW to assist ongoing deliveries or other emergencies needing HEW attention  **Control:** - | Unidirectional communication (HEW receive reminders on clients upcoming appointment, HEW send SMS to supervisor on number of vaccines needed + submission of vaccine report. HEW receive SMS reminder on stock report form + HEW submission of report to HC supervisor)  Bidirectional communication (emergency call) | 3 | Registering pregnancies | **^ Proportion of ANC service utilisation:** high for all group at baseline and post intervention (86.9% to 98.93%).  **^ Proportion of ANC visits >4:**  Intervention1 pre 45.32%, post 59.84%, P=0.001.  Intervention2 pre 15.80%, post 31.50% P=0.001.  **^ Proportion of ANC delivered by HEWs:** Intervention1 pre 19.01%, post 28.27%. Intervention2 pre 5.21%, post 29.75%.  **^ Proportions of deliveries attended by health professional:** Intervention1 pre 26.79%, post 55.23%. Intervention2 pre 41.96%, post 63.54%, Control pre 21.79%, post 52.05%.  **Proportion of home delivery:** Intervention1 pre 61.57%, post 33.73%. Intervention2 pre 50.70%, post 35.82%. Control pre 72.8%, post 58.46%.  **Child immunization coverage**: Intervention1 pre 88.63%, post 58.31%. Intervention2 pre 76.19%, post 58.72%, Control pre 81.82%, post 62.98%. |
|  |  |  |  | 4 | Collecting data on pregnant women |  |
|  |  |  |  | 7 | Communication between HEW and HC supervisor |  |
|  |  |  |  | 8 | Schedule for all ANC visits and upcoming vaccinations |  |
| 10.  Atukunda et al. (2021) Uganda [41] | **Design:** RCT  **Methods:** Interviews conducted at baseline, six and 12 months postpartum alongside blood samples  **Setting:** The maternity ward of a regional referral hospital in Southwestern Uganda  **Population:** Women living with HIV (WLWH)  **Size:** Total(pre/post)=320/317  Intervention=160/158, Control=160/159 | **Intervention:** Family planning counselling and vouchers (incentive) + daily SMS reminders for women choosing oral contraceptive pills or condoms. Women choosing injectable contraception received reminders monthly, and all others were sent weekly reminders  **Control:** Routine FP counselling | Unidirectional communication (text messages) | 1 | Reminders to take contraceptive pill | **^ Use of effective contraceptive method at 12 months:** Intervention group 79.8%, Control group 69.2%, OR 1.75 (95% CI 1.24 to 2.95) P= 0.003.  **^ Pregnancy in the 1^st^ year postpartum:** Intervention group 1.9%, Control group 8.8%, OR 0.20 (95% CI 0.05 to 0.62) P= 0.006. |
| 11.  Mangwi Ayiasi et al. (2016) Uganda [100] | **Design:** RCT  **Methods:** Socio-demographic data collected at enrolment. Outcome data collected using structured questionnaires  **Setting:** 16 health centers in Masindi (10 HCs) and Kiryandongo (6 HCs) districts  **Population:** Pregnant women <=28 weeks of GA recruited during first ANC visit  **Size:** Total(pre/post)=1644/1386  Intervention=751/627, Control=893/758  Total VHTs (village health teams)=48 | **Intervention:** VHTs made two prenatal home visits (soon after enrolment and 4 weeks later) and 1 postnatal home visit (within three days after delivery) to provide educational messages for maternal and newborn care. VHTs had unlimited access (phone call) to professional health workers for consultations  **Control:** Routine education in clinic | Bidirectional communication (phone call) | 7 | Communication between village health teams and professional health workers | **^ Health facility delivery:** Intervention group 87.7%, Control group 27.6%, adj. OR 17.94 (95% CI 6.26 to 51.37) P<0.001. **Antenatal care attendance (3 or more):** Intervention group 85.3%, Control group 71.2%, adj. OR 1.82 (95% CI 0.65 to 5.09) P=0.26.  **Birth preparation (adequate):** Intervention group 51.8%, Control group 20.8%, adj. OR 2.59 (95% CI 0.81 to 8.30) P=0.11.  Neonatal outcomes showed significant associations with the intervention, except for breastfeeding practices.  **Cord care (clean):** 59.1% vs 28.5%, adj. OR 3.05(95% CI 1.81-5.12).  **Thermal care (adequate):** 69.2% vs 17.9%, adj. OR 7.58 (95% CI 2.52 to 22.82) P<0.001.  **Breastfeeding practices (appropriate):** 67.4% vs 65.6%, adj. OR 1.26 (95% CI 0.70 to 22.29) P=0.44.  **Newborn care seeking (timely):** 86.5% vs 62.5%, adj. OR 4.93 (95% CI 1.59 to 15.31) P=0.006. |
| 12.  Bangal et al. (2017) India  [42] | **Design:** RCT  **Methods:**  **Setting:** Rural medical collage  **Population:** Pregnant women  **Size:** Total(pre/post)=400/327  Intervention=200/157, Control=200/170 | **Intervention:** Routine ANC + phone calls as reminder of next visit and text messages on important aspects of antenatal care at regular intervals.  **Control:** Routine ANC | Unidirectional communication (text messages)  Bidirectional communication (Phone call) | 1 | Reminders and educational information | **^ Antenatal visits (>4):** Intervention group 57.5%, Control group 23.5%.  **^ Place of delivery (any hospital):** Intervention group 89%, Control group 91.5%.  **^ Postnatal visits (>=1):** Intervention group 94.11%, Control group 70.1%, P<0.0001.  **Iron and calcium supplementation ≥ 3 months:** Intervention group 81%, Control group 69%, p<0.0001.  **Tetanus injection:** Intervention group 97%, Control group 87%, P<0.0002. **Weight gain > 10kg:** Intervention group 35%, Control group 25%. **Ultrasound examination:** Intervention group 93%, Control group 79%, P<0.0001.  **Anaemia:** Intervention group 36%, Control group 45%, p=0.0119. **Need for blood transfusion to correct for anaemia:** Intervention group 0%, Control group 3%. **Need for parenteral iron therapy to correct for anaemia:** Intervention group 2%, Control group 5%. **Proportion of LBW babies:** Intervention group 30%, Control group 35%. **Perinatal mortality:** Intervention group 2.94%, Control group 3.82%. **Medical obstetric care:** Intervention group 24%, Control group 31%. |
| 13.  Bangure et al. (2015) Zimbabwe  [43] | **Design:** RCT  **Methods:** Interview administered questionnaire  **Setting:** Rimuka, Waverley Chemukute and Kadoma General hospital city clinics  **Population:** Women or caregivers recruited soon after delivery or during 3rd to 7th day visit.  **Size:** Total=304  Intervention=152, Control 152 | **Intervention:** Routine care and health education + text message reminder indicating the next appointment date 7 days, 3 days, and 1 day before the appointment was scheduled. Messages were sent to remind of the 6th, 10th and 14th weeks appointments.  **Control:** Routine care | Unidirectional communication (text messages) | 1 | Vaccination appointment reminders | **^ Immunisation coverage (3 doses of polio, pentavalent, and pneumococcal vaccines) at week 6:** Intervention group 96.7%, Control group 82.2%. **^At week 10** Intervention group 96.1%, Control group 80.3%. **^At week 14** Intervention group 94.7%, Control group 75.0%. A significant difference at all time points (p<0.001).  **Delay in immunisation appointment:** Controls had a greater delay in vaccination. 93%, 87%, and 82% of the intervention group received vaccinations on the scheduled day for week 6, 10, and 14 respectively. 24%, 17%, and 8% of the control group received vaccinations on the scheduled day for week 6, 10, and 14 respectively. The median delay receiving vaccination in the control group was 2 days for week 6 vaccinations, 5 days for week 10 vaccinations, and 10 days for week 14 vaccinations. |
| 14.  Bellad et al. (2020) India [44]  *Data overlap with Von Dadelszen et al. (2020)* | **Design:** Cluster RCT (CLIP intervention)  **Methods:** Review of facility medical records. Data collected at enrolment, soon after delivery, and 42 days postpartum  **Setting:** 12 PHC in Belagavi and Bagalkote districts, rural Karnataka  **Population:** Married pregnant women  **Size:**  Total Health Worker=148  Total pregnant women(pre/post)=14783/13017  Intervention=7839/6908, Control=6944/6109 | **Intervention:** Community engagement with leaders, Pregnant women, mothers, and other stakeholders to promote content regarding hypertension/(pre-)eclampsia, and more + CLIP visits using the PIERS On the Move tool (POM) +  CHW-led CLIP antenatal contacts every 4 weeks (<28wks) and every 2 weeks (28-35wks) and once weekly (<35wks) + 4 times postpartum. Visits are guided by CLIP POM mhealth app  **Control:** Routine ANC | Unidirectional communication (data collection) | 3 | Maternal and Newborn Health Registry | **^ All-cause maternal mortality:** Intervention group 0.1%, Control group 0.1%, Adj. OR 0.59 (95% CI 0.14 to 2.77) P=0.47.  **^ All-cause maternal morbidity:** Intervention group 4.7%, Control group 4.7%, Adj. OR 1.04 (95% CI 0.76 to 1.43) P=0.80.  **^ All-cause perinatal mortality and late neonatal mortality:**  Intervention group 4.7%, Control group 4.2%, Adj. OR 1.05 (95% CI 0.89 to 1.24) P=0.56.  **^ All-cause neonatal morbidity:**  Intervention group 10.4%, Control group 11.4%, Adj. OR 0.89 (95% CI 0.67 to 1.17) P=0.39.  **Birth preparedness and complication readiness:** Intervention group 71.3%, Control group 84.5%, Adj. OR 0.65 (95% CI 0.03 to 14.4) P=0.717.  **Delivery in facility with emergency care:** Intervention group 65.5%, Control group 68.3%, Adj. OR 0.9 (95% CI 0.60 to 1.34) P=0.482.  **Proportion of facility births:** Intervention group 76.4%, Control group 77.5%, Adj. OR 1.06 (95% CI 0.92 to 1.21) P=0.302. |
|  |  |  |  | 4 | Blood pressure measurements and test for proteinuria |  |
|  |  |  |  | 5 | Data transfer from POM mobiles to central REDCap server |  |
|  |  |  |  | 6 | Electronic decision support with pictograms and visual prompts directing CHWs to recommend appropriate care |  |
| 15.  Bigna et al. (2014) Cameroon [45] | **Design:** RCT  **Methods:** Socio-demographic data collected during recruitment. Outcome data was collected from records.  **Setting:** Three regions representing rural (Goulfey), semi-urban (Kousseri) and urban (Essos) settings  **Population:** Adult-child (carer-patient) pairs where child was infected with HIV or had been exposed to HIV  **Size:** Total=242,  Intervention1=60, Intervention2=60, Intervention3=61, Control=61 | **Intervention1:** Text message reminder were sent 2 days before a scheduled appointment.  **Intervention2:** Call were made 2 days before scheduled appointment.  **Intervention3:** Text message were sent 3 days before scheduled appointment and called 2 days before the appointment.  **Control: -** | Unidirectional communication (text messages) for Intervention1 and 3.  Bidirectional communication (Phone-call) for intervention2 and 3. | 1 | Appointment reminders | **^ Attendance for HIV care:**  Intervention1 75%, control 51%, OR 2.9 (95% CI 1.3 to 6.3) P=0.012.  Intervention2 85%, control 51%, OR 5.5 (95% CI 2.3 to 13.1) P=0.0002.  Intervention3 89%, control 51%, OR 7.5 (95% CI 2.9 to 19.0) P<0.0001.  All interventions improved attendance compared to control. |
| 16-17  Billah et al. (2022a, 2022b) Bangladesh [46,47] | **Design:** Cluster RCT  **Methods:** Socio-demographic data and IYCF knowledge collected at enrolment. Outcome data collected during follow-up visits  **Setting:** Bahubal and Nabiganj sub-districts in Sylhet Division in the north-eastern side of Bangladesh  **Population:** Community health workers and pregnant women and their children 0-23 months of age  **Size:** Total(pre): 1500 (250 per intervention arm) and 500 in control arm.  Total(post): 1250 live births (847 in intervention arms and 403 in control arms) for breastfeeding practice data | **BBC(all arms):** extensive counselling and practical EBF demonstrations. 2 ANC session during 3. trimester and monthly during the first 6 months of the child´s life using electronic job aid containing BF modules.  **ARM1:** BBC + lipid-based prenatal nutrient supplement[PNS] for pregnant women + lipid-based complementary nutrient supplement[CFS] for children.  **ARM2:** BBC + PNS  **ARM3:** BBC + CFS  **ARM4:** BBC  **Control: -** | Unidirectional communication (BF modules delivered via tablet) | 1 | Educational BF modules (continuation of EBF, common challenges and solutions | **^ EBF 1 month:** Intervention group 554, Control group 277, RR 1.01 (95% CI 0.97 to 1.05) P=0.569.  **^ EBF 2 months:** Intervention group 605, Control group 297, RR 1.01 (95% CI 0.98 to 1.05) P=0.478.  **^ EBF 3 months:** Intervention group 647, Control group 302, RR 1.08 (95% CI 1.02 to 1.13) P=0.004.  **^ EBF 4 months:** Intervention group 707, Control group 333, RR 1.16 (95% CI 1.08 to 1.23) P<0.001.  **^ EBF 5 months:** Intervention group 727, Control group 335, RR 1.22 (95% CI 1.12 to 1.33) P<0.001.  **Early initiation of BF:** Intervention group 74.4%, Control group 75.2%, RR 1.00 (95% CI 0.93 to 1.07) P=0.929.  **Colostrum feeding:** Intervention group 97.8%, Control group 96.9%, RR 1.01 (95% CI 1.00 to 1.00) P=0.196.  **Pre-lacteal feeding:** Intervention group 6.2%, Control group 11.0%, RR 0.54 (95% CI 0.39 to 0.76) P=0.001.    **Dietary diversity score for children 6-23 months of age (mean±SD):** Intervention group 2.39±1.39, Control group 2.30±1.38, OR 0.09 (95% CI 0.02 to 0.16), P<0.05  **Dietary diversity score – egg (mean±SD):** Intervention group 19.2, Control group 16.6, OR 0.22 (95% CI 1.03 to 1.45) P<0.05  **Dietary diversity score – flesh food (mean±SD):** Intervention group 53.7, Control group 49.6, OR 1.32 (95% CI 1.11 to 1.57) P<0.05.  *No difference observed for other food groups* |
|  |  |  |  | 3 | Live birth registrations |  |
|  |  |  |  | 4 | Data collection on site uploaded to trial server |  |
|  |  |  |  | 8 | Notifications for scheduled home visits and new live births |  |
| 18.  Bogale et al. (2021) Palestine [48] | **Design:** Cluster RCT (eRegCom trial)  **Methods:** 13-item Cambridge Worry Scale (CWS), phone interviews to collect sociodemographic data and data on satisfaction with ANC services  **Setting:** 138 public primary HCs  **Population:** pregnant women  **Size:** Total=454 women  Intervention=239 women  Control=215 women | **Intervention (TCC):** Training of healthcare providers on how to enrol women in TCC program (a series of individualised and automated text messages according to GA) + reminders before an appointment (1 week, 3 days, and 24h) and after (24h)  **Control:** | Unidirectional communication (text messages) | 1 | Educational information (pregnancy related high risk conditions) + appointment reminders | **^ Difference in mean±SD total CWS:**  Intervention group 1.8±1.9, Control group 2.0±1.9, Unadj. difference -0.2 (95% CI -0.3 to -0.1), adjusting for cluster effect -0.16 (95% CI -0.31 to -0.01)  **Satisfactions with ANC services:** no difference between groups |
| 19.  Brown et al. (2016) Nigeria [49] | **Design:** C-RCT  **Methods:** Group A: questionnaire to collect Socio-demographic data (A, B, C, D), immunisation data (A, B, C,D), recorded reminders (A, C), appraisal of reminder system (A, C). Questionnaire and observation checklist were used to assess knowledge of health professionals undergone training (B, C)  **Setting:** Four local government urban and sub-urban areas (three intervention groups and one control group)  **Population:** Children aged 0-12 months old paired with their mother  **Size:** Total(pre/post)=605/595  Group A=153/148, Group B=151/150  Group C=149/147, Group D=152/150. | **Group A:** Routine care + phone call as reminders to parents 2 days and 1 day before scheduled vaccination appointment.  **Group B:** Routine care + 2 days refresher training for primary health care providers (four WHO modules).  **Group C:** Routine care + A and B combined.  **Group D:** Routine care | Bidirectional communication (phone calls) | 1 | Vaccination appointment reminders | **^ Immunisation completion rate (1 Bacillus Calmette-Guerin [BCG] dose, >=4 doses of oral polio vaccines, 3 Diphtheria doses, 3 Hepatitis B doses, 1 dose each of Measles and Yellow fever):** Group A 98.6%, Group B 70%, Group C 97.3%, Group D 57.3%.  Children in group A (RR 1.72, 95% CI 1.50 to 1.98) and group C (RR 1.70 95% CI 1.47 to 1.95) were 72% and 70% more like to complete immunisation respectively, compared to control. Group B marginally differed from control group (RR 1.22 95% CI 1.03 to 1.45). |
|  |  |  |  | 9 | Refresher course on vaccination of preventable diseases, injection, building community support, and communication with mothers about immunisation (WHO modules) |  |
| 20.  Carmichael et al. (2019) India [50] | **Design:** Cluster RCT (Ananya program)  **Methods:** data collected using surveys pre and post intervention  **Setting:** Saharsa and 7 other districts in Bihar. 70 sub-health centers (35 control, 35 intervention)  **Population:** Frontline workers (FLW) including ASHAs and AWW and beneficiaries (maternal)  **Size:**  Total FLW (pre/post)= 573/572  Intervention=319/316, Control=254/256  Women(pre/post)=1559/1553  Intervention=790/809  Control=769/744 | **Intervention:** Ananya program + FLWs equipped with ICT_CSS (The information Communication Technology Continuum of Care Services) mobile health tool with access to relevant content, beneficiary tracking, delivery of appropriate services, and supervision  **Control:** Ananya program (improving behaviours related to FP, ANC, delivery preparation, PNC, complementary feeding, child immunisation) | Unidirectional communication (tools, checklists, data collection) | 1 | Health information | **^ >= 3 ANC home visits:** Intervention pre 37%, Control pre 24%, P<0.01. Intervention post 48%, Control post 29%, P=0.05  Percent difference attributable to intervention 6.6, P=0.31.  **^ Home visit after delivery (<24h):** Intervention post 34%, Control post 35%, P=0.9  **^ Home visit within 1 week:** Intervention post 72%, Control post 60%, P<0.01  **Facility delivery:** Intervention pre 76%, Control pre 77%, P=0.80  Intervention post 85%, Control post 84%, P=0.93  **Early initiation of BF:** Intervention pre 44%, Control pre 47%, P<0.58. Intervention post 74%, Control post 62%, P<0.01  **EBF 6 months postpartum:** Intervention pre 34%, Control pre 38%, P=0.25. Intervention post 62%, Control post 61%, P=0.63  **Full immunisation (except measles)**: Intervention pre 41%, Control pre 40%, P=0.75. Intervention post 59%, Control post 55%, P=0.51.  *Many more outcomes reported* |
|  |  |  |  | 4 | Data collection and tracking beneficiaries |  |
|  |  |  |  | 6 | Supervisory tools, checklists, and guided protocols |  |
|  |  |  |  | 8 | Automated scheduling home visits and tracking child immunisation |  |
| 21.  Chan et al. (2019) Hong Kong [51] | **Design:** RCT  **Methods:** Survey conducted at baseline (socio-demographic data) and 4 weeks after delivery for outcome data collection (EPDS, SF-12, DASS,  **Setting:** ANC clinic in Kwong Wah Hospital (KWH)  **Population:** First time pregnant women < 24weeks of GA  **Size:** Total(pre/post)=660/443  Intervention=330/218, Control=330/225 | **Intervention:** Routine ANC + Smartphone-based app for antenatal education (same as delivered during classes offered at clinic) + platform to ask question to obstetrician (private messaging + sharing of Frequently asked questions accessible to all)  **Control:** Routine ANC (also access to classes providing same information as app) | Unidirectional communication  (Material on app)  Bidirectional communication (with obstetrician) | 1 | Educational information (nutrition, infant caring, vaccination, video demonstration of what to expect when delivering a baby) | **^ EPDS score (mean±SE):** Intervention group pre 7.3±4.6, post 5.3±4.4, Control group pre 7.2±4.6, post 5.9±4.7, mean difference 0.65 (-1.29 to 0.00) P=0.049.  **DASS score – Anxiety (mean±SE):** Intervention group pre 3.1±2.7, post 1.9±2.1, Control group pre 2.8±2.6, post 1.8±2.3, mean difference 0.01 (-0.30 to 0.32) P=0.94.  **DASS score – Stress (mean±SE):** Intervention group pre 3.9±3.5, post 3.0±3.1, Control group pre 3.8±3.4, post 2.9±3.1, mean difference 0.07 (-0.35 to 0.50) P=0.74.  **SF-12 – Physical component (mean±SE):** Intervention group pre 45.5±7.0, post 48.8±6.9, Control group pre 46.4±7.0, post 48.8±7.2, mean difference 0.32 (-0.71 to 1.34) P=0.54.  **SF-12 – Mental component (mean±SE):** Intervention group pre 48.6±8.9, post 51.2±8.4, Control group pre 48.2±8.9, post 51.2±9.0, mean difference -0.15 (-1.39 to 1.09) P=0.81. |
| 22.  Chowdhury et al. (2019) India [52] | **Design:** Quasi-experimental study (Aponjon)  **Methods:** Structured questionnaire for outcome data collection  **Setting:** 1) Rural service area (Matlab) of icddr,b, 2) Slum in Dhaka (Bhashantek), 3) Urban area of Dhaka (Brahmanbaria), 4) rural area in Brahmanbaria district.  **Population:** Mothers with infants <=6 months of age and mothers with infants between 6 months and 1 year  **Size:** Total mothers with infants =<6months (post)=612  Intervention=243, Control=369  Total mothers with infants >6months (post)=786  Intervention=332, Control=454 | **Intervention:** Aponjon users (app message service providing educational audio messages)  **Control:** Aponjon non-users | Unidirectional communication (audio messages) | 1 | Educational information (knowledge and recommended practices related to maternal healthcare during the last pregnancy, around the time of delivery and 6 months postpartum) | **^ Exposure to Aponjon services (3-5months)** was not associated with increased maternal healthcare knowledge (Adj. OR 1.20 (95% CI 0.80 to 1.81)) or practice (Adj. OR 1.18 (95% CI 0.81 to 1.72)).  **^ Exposure to Aponjon services (6-9months)** was associated with increased maternal healthcare knowledge (Adj. OR 2.83 (95% CI 1.57 to 5.09)) and practice (Adj. OR 3.38 (95% CI 1.77 to 6.48)).  **^ Exposure to Aponjon services (3-5months)** was not associated with increased newborn healthcare knowledge (Adj. OR 1.19 (95% CI 0.67 to 2.12)) or practice (Adj. OR 0.77 (95% CI 0.42 to 1.64)).  **^ Exposure to Aponjon services (6-9months)** was associated with increased newborn healthcare knowledge (Adj. OR 1.46 (95% CI 1.05 to 2.03)) but not practice (Adj. OR 1.22 (95% CI 0.91 to 1.64)). |
| 23.  Coleman et al. (2017) South Africa [53]  *Potential data overlap with Coleman et al. (2020)* | **Design:** Quasi-experimental study  **Methods:** Demographic data collected from SMS recruitment database, A maternal register database from the delivery site (ANC/delivery information) and SMS research database, ANC visit record cards, and PCR registration book  **Setting:** One public health facility in Hillbrow, District of Johannesburg, rural impoverished population  **Population:** HIV women attending their first ANC visit  **Size:** Total(valid data for main outcome)= 639  Intervention=192, Control=447 | **Intervention:** Routine care + Maternal health SMS sent twice weekly throughout pregnancy and for one year postnatally timed with stage of pregnancy and age of child including appointment reminders + HIV/PMTCT-related messages (PCR testing reminders, importance of ART adherence)  **Control:** Routine care | Unidirectional communication (text messages) | 1 | ANC/PNC and appointment reminders + PCR testing reminders + Supportive and informative information on maternal and infant health topics such as healthy eating, psychosocial support, PMTCT support messages, delivery planning etc. + HIV/PMTCT related | **^ Uptake of HIV PCR testing within six weeks postpartum:** Intervention group 81.3%, Control group 75.4%, RR 0.91 (95% CI 0.81 to 1.01) P=0.064.  **Proportion of vaginal deliveries:** Intervention group 84.2%, Control group 76.5%, RR 1.1 (95% CI 1.02 to 1.19) P=0.030.  **Proportion of low-birth weight babies:** Intervention group 1.4%, Control group 9.9%, RR 0.14 (95% CI 0.02 to 1.07) P=0.054.  **ANC visits attended(>=4):** Intervention group 82.2%, Control group 58.5%, RR 1.4 (95% CI 1.15 to 1.72) P=0.001.  *HIV infected women were not excluded from Coleman et al. (2020). This group are likely to be included in ANC visits results below.* |
| 24.  Coleman et al. (2020) South Africa [54] | **Design:** Quasi-experimental study  **Methods:** Socio-demographic data collected during enrolment. EPI coverage collected from RTH booklets.  **Setting:** 6 PH facilities offering ANC and PNC/EPI services in the inner city of Johannesburg and Hillbrow. Urban population  **Population:** All pregnant women attending intervention site clinics between 11^th^ and 39^th^ week of pregnancy. Controls recruited from PNC services in other clinics.  **Size:** Total(pre/post)=365/177  Intervention=181/87, Control=175/90 | **Intervention:** Routine care + Maternal health SMS sent twice weekly throughout pregnancy and for one year postnatally timed with stage of pregnancy and age of child including appointment reminders  **Control:** Routine care | Unidirectional communication (text messages) | 1 | ANC/PNC appointment reminders + Supportive and informative information on maternal and infant health topics such as healthy eating, psychosocial support, PMTCT support messages, delivery planning etc. | **ANC visits attended (>=2):** Intervention group 97.7%, Control group 84.4%, Adj. OR 7.77 (95% CI 2.06 to 29.29) P=0.01.  **(>=3):** Intervention group 92.0%, Control group 65.6%, Adj. OR 5.66 (95% CI 3.16 to 10.15) P=0.00.  **^ (>=4):** Intervention group 72.4%, Control group 45.6%, Adj. OR 3.21 (95% CI 1.73 to 5.98) P=0.01.  **Mean number and ANC visits attended:** Intervention group 4.4 (4.0 to 4.7), Control group 3.2 (2.9 to 3.5), Adj. OR 3.16 (95% CI 1.56 to 6.42) P=0.009.  **^ Fully immunized:** Intervention group 95%, Control group 89%, Adj. OR 1.73 (95% CI 0.54 to 5.52) P=0.282.  No difference found for any individual vaccination in the immunization scheme.  **Completion of continuum of care (>=4 ANC visits + Full immunization up to 1 years of age.):** Intervention group 70%, Control group 41%, Adj. OR 3.2 (1.63 to 6.31) P=0.007. |
| 25.  Dissieka et al. (2019) Côte d’Ivoire [55] | **Design:** RCT  **Methods:** Socio-demographic information collected at enrolment during first BCG vaccination visit. Attendance data collected from records  **Setting:** 29 Health facilities (7 urban, 12 semi-urban, and 10 rural) in Korhogo district in the north-central region  **Population:** Mother/caretaker-child pairs  **Size:** Total= 1596  Intervention=798, Control=798 | **Intervention:** SMS or voice reminders prior to scheduled facility visits for vaccinations (Pentavalent 1, 2, and 3 at 6, 10, and 14 weeks respectively, VAS at 6 months, MMR/yellow fever at 9 months). Two additional reminders were sent in the event of non-attendance 3 days after missed appointment and 2 days before the next vaccination  **Control:** instructed to refer to child’s vaccination card | Unidirectional communication (text messages) | 1 | Vaccination appointment reminders | **^ Pentavalent 1 attendance:** Intervention group 86.6%, Control group 76.1%, Adj. OR 2.85 (95% CI 1.85 to 4.37) P<0.001.  **^ Pentavalent 2 attendance:** Intervention group 81.0%, Control group 67.3%, Adj. OR 2.80 (95% CI 1.88 to 4.17) P<0.001.  **^ Pentavalent 3 attendance:** Intervention group 74.2%, Control group 58.3%, Adj. OR 2.68 (95% CI 1.84 to 3.91) P<0.001.  **^ Vitamin A Supplementation attendance:** Intervention group 64.7%, Control group 40.7%, Adj. OR 5.67 (95% CI 3.48 to 9.23) P<0.001.  **^ MMR/yellow fever attendance:** Intervention group 60.7%, Control group 37.8%, Adj. OR 4.52 (95% CI 2.84 to 7.20) P<0.001.  58.3% of all infants in the intervention group and 35.7% in the control completed all vaccinations and supplementations, P<0.001. |
| 26.  Domek et al. (2019) Guatemala [56] | **Design:** RCT  **Methods:** Socio-demographic data collected at enrolment using baseline survey. Outcome data collected during follow-ups.  **Setting:** Four government PH clinics of the MPHSA serving a low-income population, 2 rural (Southwest region) and 2 urban metropolitan (Guatemala City) clinics  **Population:** Infants between the ages of six weeks to six months  **Size:** Total(pre/post)=720/662  Intervention=358/329, Control=362/333 | **Intervention:** Routine care + vaccination cards + vaccination appointment reminders 3, 2, and 1 day prior to scheduled appointment according to primary national immunisation series up until 6 months of age.  **Control:** Routine care + vaccination cards | Unidirectional communication (text messages) | 1 | Vaccination appointment reminders | **^ Immunisation completion rates:** Control group 87.8%, Control group 89.2, P=0.59.  **Immunisation timeliness for visits 2 (on scheduled date):**  Intervention group 42.2%, Control group 30.7% P=0.001.  **Immunisation timeliness for visits 2 (7 days late):** Intervention group 71.0%, Control group 63.5%, P=0.03.  **Immunisation timeliness for visits 3 (on scheduled date):**  Intervention group 37%, Control group 34%, P=0.05.  **Immunisation timeliness for visits 3 (7 days late):** Intervention group 61.1%, Control group 57.4%, P=0.33. |
|  |  |  |  | 5 | Electronic immunisation records |  |
| 27.  Dryden-Peterson et al. (2015) Botswana [57] | **Design:** Cluster RCT (Stepped Wedge”)  **Methods:** Obstetric, maternity, and laboratory records  **Setting:** 20 highest volume antenatal clinics in greater Gaborone  **Population:** ART naïve, HIV infected pregnant women >26 weeks of GA  **Size:** Total(pre/post)=366/307  Intervention=189/154, Control=177/153 | **Intervention:** Pregnant women attending ANC in clinics where clinic staff had undergone training in timely CD4 count and ART-initiation + development of solutions to identified challenges regarding clinic performance + SMS based platform providing CD4 results directly to clinic + longitudinal support from study team  **Control:** Pregnant receiving routine care | Unidirectional communication (text messages with test result) | 7 | Feedback loop between clinics and laboratories reducing the turnaround time between HIV testing and result reporting | **^ Complete phlebotomy for CD4 enumeration by 25 weeks of GA:** Intervention group 59%, Control group 51%, Adj. OR 1.20 (95% CI 0.52 to 2.78) P=0.66  **^ Initiating ART by 30 weeks of GA:** Intervention group 36%, Control group 24%, Adj. OR 1.01 (95 % CI 0.46 to 2.22) P=0.98 |
| 28.  Ekhaguere et al. (2019) Nigeria [58] | **Design:** RCT  **Methods:** Socio-demographic data collected at enrolment and outcome data collected from immunisation clinic records and mid-study phone call to participants.  **Setting:** Mother and Child Hospital Ondo Town (MCH-Ondo) and Akure (MCH-Akure) in Ondo state  **Population:** Mother and Newborn delivered in MCH-Ondo and Akure  **Size:** Total=600  Intervention=300, Control=300 | **Intervention:** Routine care + Immunisation reminder send 2 and 1 day prior to scheduled appointment  **Control:** Routine care | Unidirectional communication (voice recording, email or text) | 1 | Vaccination appointment reminders | **^ Pentavalent 1 attendance:** Intervention group 95%, Control group 97%, RR 0.98 (95% CI 0.95 to 1.02) P=0.31.  **Timeliness**: 1.03 (95% CI 0.96 to 1.10) P=0.43.  **^ Pentavalent 2 attendance:** Intervention group 92%, Control group 93%, RR 0.99 (95% CI 0.95 to 1.04) P=0.76.  **Timeliness**: 1.02 (95% CI 0.95 to 1.09) P=0.65.  **^ Pentavalent 3 attendance:** Intervention group 86%, Control group 81%, RR 1.05 (95% CI 0.98 to 1.13) P=0.15.  **Timeliness**: 1.09 (95% CI 1.01 to 1.17) P=0.04.  **Measles vaccination attendance:** Intervention group 74%, Control group 66%, RR 1.12 (95% CI 1.01 to 1.25) P=0.03. **Timeliness**: 1.13 (95% CI 1.02 to 1.26) P=0.02.  **All immunisations:** Intervention group 57%, Control group 47%, RR 1.22 (95% CI 1.04 to 1.43) P=0.03.  **Timeliness**: 1.22 (95% CI 1.04 to 1.43) P=0.01. |
| 29.  Eslami et al. (2018)  Iran [59] | **Design:** RCT  **Methods:** Socio-demographic and obstetrics questionnaire completed at baseline. FBS and OGTT assessed between 24 and 28 week of GA  **Setting:** 12 Health centers in Tehran  **Population:** pregnant women, BMI>25, 16-20 weeks of GA  **Size:** Total=140  Intervention=70, Control=70 | **Intervention:** Routine ANC + 60-90min group session, 30min QA session + ability to ask questions over the phone at any time + educational booklet + educational text messages sent every 2 days for 4 weeks post session  **Control:** Routine ANC | Unidirectional communication (text messages) | 1 | Educational information | **FBS(mean±SD):** Intervention group 83.0±8.5, Control group 81.9±10.8, mean difference 1.1 (95% CI -2.1 to 4.3) P=0.505.  **OGTT 1 hour post(mean±SD):** Intervention group 162.7±17.1, Control group 161.2±16.8, mean difference 1.5 (95% CI -4.2 to 7.1) P=0.605.  **OGTT 2 hour post(mean±SD):** Intervention group 124.8±15.8, Control group 124.5±18.6, mean difference 0.3 (95% CI -5.4 to 6.1) P=0.903.  **GDM:** Intervention group 21.4%, Control group 24.3, mean difference 0.8 (95% CI 0.4 to 1.9) P=0.687. |
| 30.  Eze and Adeleye (2015) Nigeria [60] | **Design:** RCT  **Methods:** Baseline coverage at collected from immunisation records. Follow-up survey to collected outcome and socio-demographic data.  **Setting:** 8 Health facilities in Egor local government area of Edo State.  **Population:** Mothers bringing their infant for first immunisation (or second for those who received first shot at bedside)  **Size:** Total(pre/post)= 1001/905  Intervention=500/452, Control=501/452 | **Intervention:** Routine immunisation care + SMS reminder 1 day before upcoming appointment and recall message sent when appointments were missed.  **Control:** Routine immunisation care | Unidirectional communication (text messages) | 1 | Immunisation appointment reminders | **^ Early receipt of DPT3 (18w from first visit or 12 weeks from visit depending when they received DPT1):** OR 1.47 (95% CI 1.1 to 2.0) |
| 31.  Fahami et al. (2014) Iran [61] | **Design:** Quasi-experimental study  **Methods:** Questionnaire of personal – fertility information and awareness towards breastfeeding completed pre and post education (2 weeks later via phone)  **Setting:** Shahid Beheshti Hospital in Isfahan  **Population:** New mothers still hospitalised after delivery  **Size:** Total=73,  Intervention=35, Control=35 | **Intervention:** Multi-media electronic education (text, graphic, animation sound, images, digital videos) twice weekly for 30 min.  **Control:** illustrated educational booklet | Unidirectional communication (text messages) | 1 | Educational information (BF benefits, composition of colostrum and breast milk, health and skills of breastfeeding and handling the BF) | **Breastfeeding awareness (mean±SD):** Intervention group pre 4.02±0.01, post 13.12±1.2, P=0.01. Control group pre 3.42±0.12, post 9.22±0.43, P=0.002  *No comparison between groups.* |
| 32.  Fedha et al. (2014) Kenya [62] | **Design:** RCT  **Methods:** Structured questionnaires administered at baseline and 2 weeks postpartum  **Setting:** Two health facilities, at Njoro devision, Nakuru  **Population**: Pregnant women 12-36 weeks of gestation  **Size:** Total(pre/post)=397/395  Intervention=191/191, Control=206/204 | **Intervention:** Routine care + bi-weekly reminders on antenatal clinic visits. Text messages with advice about maternal and child health, iron + other micronutrient supplementation and diet. Counselling was available to participants via phone (SMS or call) to address queries.  **Control:** Routine care | Unidirectional communication (SMS)  Bidirectional communication (Phone calls) | 1 | Antenatal care appointment reminders and educational information | **^ Attended >=4 ANC visits:** Intervention group 96.4%, Control group 92.3%, P=0.002.  **^ Discussed place of delivery:** Intervention group 75.9%, Control group 62.6%, P=0.004.  **^ Received diet counselling:** Intervention group 95.3%, Control group 89.3%, P=0.027.  **^ First tetanus toxoid injection:** Intervention group 100%, Control group 99.5%, P=0.335.  **^ HIV counselling:** Intervention group 96.3%, Control group 95.1%, P=0.559.  **^ Vitamin supplementation:** Intervention group 39.8%, Control group 23.8%, P=0.001. **^ Deworming drugs:** Intervention group 74.3%, Control group 59.2%, P=0.001.  **^ Iron supplementation:** Intervention group 91.6%, Control group 87.4%, P=0.170. **^ Malaria prophylaxis:** Intervention group 80.6%, Control group 48.5%, P<0.0001.  **^ Intrauterine fetal death**: Intervention group 1%, Control group 1.5%, P=0.715. **^ Neonatal deaths:** Intervention group 1%, Control group 3.4%, P= 0.269. **^ Proportion of infant crying immediately at birth:** Intervention group 97.4%, Control group 95.6%, P= 0.765.  **^ Place of delivery:** Intervention group 88%, Control group 72.8%, P= 0.000. |
| 33.  Fikawati et al. (2019) Pakistan  [63] | **Design:** Quasi-experimental study  **Methods:** Socio-demographic data collected at enrolment. Delivery information collected from medical records. Outcome data collected via structured questionnaires.  **Setting:** Cipayung Sub-district, Depok City, West Java  **Population:** Mother-infant pair  **Size:** Total(pre/post)=197/169, Group1=50/40, Group2=59/53, Group3=39/33, Group4=49/43 | **Group1:** Milk supplementation for lactating mothers 5 days/week from birth until 3 months  **Group2:** contacted by cadres 1/week and were reminder to EBF until 6 months  **Group3:** Received an SMS 1/week containing information about the benefits of BF and motivation to continue **Group4:** control group | Unidirectional communication (text messages to group 3)  Bidirectional communication (phone call to group2) | 1 | Education of and support to breastfeeding women | **^ EBF at 6 months:** Group1 90%, Group2 67.9%, Group3 63.6%, Group4 67.4%  Mothers who received milk supplementation (group1) had a 5.5 times greater chance of succeeding in EBF their infants for 6 months compared to those who did not receive it. RR 5.5 (95% CI 1.5 to 20.2) P=0.011. |
| 34.  Flax et al. (2014) Nigeria  [64] | **Design:** Cluster RCT  **Methods:** Survey interviews at baseline and post-intervention  **Setting:** Female community members enrolled in microcredit groups (a programme run by an American NGO)  **Population:** Pregnant women aged between 15-45years of age  **Size:** Total(pre/post)=461/390  Intervention=229/196, Control=232/194 | **Intervention:** 7 breastfeeding learning sessions, educational text messages twice weekly for 4 months, and twice every other week for additional 3 months and song/dramas.  **Control: -** | Unidirectional communication (text messages) | 1 | Educational information | **Exclusive breastfeeding at 1 month:** Intervention group 73%, Control group 61%, OR 1.6 (95% CI 0.6-1.8).  **Exclusive breastfeeding at 3 months:** Intervention group 71%, Control group 58%, OR 1.8 (95% CI 1.1-3.0)**.**  **^ Exclusive breastfeeding at 6 months:** Intervention group 64%; Control group 43%, OR 2.4 (95% CI 1.4-4.0).  Intervention group were significantly more likely to exclusively breastfeed 6 months postpartum.  **Initiation of breastfeeding within 1 hour of delivery:** Intervention group 70%, Control group 48%, OR 2.6 (95% CI 1.6-4.1)**.**  **Gave only colostrum or breast milk within the first 3 days of life:** Intervention group 86%, Control group 71%, OR 2.6 (95% CI 1.4-5.0). |
| 35.  Flueckiger et al. (2019) Guinea  [65] | **Design:** RCT  **Methods:** Health facility records  **Setting:** Healthcare facilities in the Conakry and Kindia regions of Guinea  **Population:** All pregnant women who attended an initial ANC visit  **Size:** Total=785,  Intervention=541, Control=244 | **Intervention:** Appointment reminder one day before next scheduled prenatal care visit.  **Control:** routine care | Unidirectional communication (text messages) | 1 | Appointment reminders | **^ Proportion of ANC visits: ANC2:** Intervention group 99.1%, Control group 94.3%, P<0.001. **ANC3:** Intervention group 99.6%, Control group 81.6%, P<0.001. **ANC4**: Intervention group 99.4%, Control group 52.9%, P<0.001. **All ANC visits**: Intervention group 98.2%, Control group 52.5%, P<0.001.  **^ Malaria prevention (IPTp)**: **ANC2:** Intervention group 94.5%, Control group 94.3%, P=0.914. **ANC3:** Intervention group 97.6%, Control group 77.5%, P<0.001. **ANC4:** Intervention group 98.9%, Control group 52.2%, P<0.001. **All ANC visits:** Intervention group 92.6%, Control group 50.8%, P<0.001. |
| 36.  Foster et al. (2017) Zimbabwe [66] | **Design:** Cluster RCT (EPAZ)  **Methods:** Data collected from Health facility registers, patient cards, and data collected during the study  **Setting:** Clinics located within rural communal and commercial farming areas in Mutare and Makoni districts of Manicaland province.  **Population:** HIV positive pregnant women under 35 weeks GA, enrolled during first ANC visit  **Size:** Total(pre/post)=350/348  Intervention=189/188, Control=161/161 | **Intervention:** Mother support groups (MSGs) established at intervention clinics and planned twice/month. HIV positive mothers with recent PMTCT experienced enrolled as coordinators of an MSG. They received a phone and 10$ card. HCWs and coordinators facilitated structured learning activities (8 PMTCT topics ever 4 months cycle) for members and psychosocial support was provided during meetings.  **Control:** Routine care | Unidirectional communication (text messages) | 1 | MSG meeting reminders from coordinator to group members | **^ Retention in care at 12 months postpartum of HIV exposed infants (PMTCT, illness, immunisation etc.):**  Intervention group 69%, Control group 61%, Adj. OR 1.25 (95% CI 0.73 to 2.15) P=0.42.  **Retention in care of HIV-positive mothers at 12 months postpartum (PMTCT, ART, family planning, illness, etc.):**  Intervention group 71%, Control group 61%, Adj. OR 1.31 (95% CI 0.80 to 2.12) P=0.28.  **Full dose of ANC visits:** Intervention group 52%, Control group 46%, Adj. OR 1.31 (95% CI 0.78 to 2.18) P=0.31.  **Delivery place:** Intervention group 77%, Control group 75%, Adj. OR 1.17 (95% CI 0.65 to 2.11) P=0.59.  **Use of Family planning at 1yr postpartum:** Intervention group 71%, Control group 65%, Adj. OR 1.00 (95% CI 0.58 to 1.71) P=0.99.  **Rapid HIV testing of infant:** Intervention group 67%, Control group 62%, Adj. OR 1.38 (95% CI 0.59 to 3.24) P=0.46.  **Infants DTP/measles vaccinations:** Intervention group 74%, Control group 66%, Adj. OR 1.40 (95% CI 0.74 to 2.66) P=0.30. |
| 37-38.  Fotso et al. (2015a and 2015b) Malawi [67, 68] | **Design:** Quasi-experimental study (CCPF)  **Methods:** Household surveys conducted at baseline (socio-demographic data) and endline.  **Setting:** 4 rural HC catchment areas in the district of Balaka (intervention site) and 2 HC from Ntcheu District (control site)  **Population:** Women of childbearing age 15-49 and/or a child under 5 years of age  **Size:** Total children(pre/post) =6846/3585  Total(first two outcomes)= 3585, Intervention=2220, Control=1365  Total(included in last 2 outcomes)=2813 | **Intervention:** Weekly educational information on appropriate care seeking and health practices for pregnant women and children under 5 + reminders + Case management hotline providing health information, advice, and referrals.  **Control:** | Unidirectional communication (text messages and voice)  Bidirectional communication for case management and emergency hotline. | 1 | Educational information and reminders | **^ Exclusive breastfeeding at 6 months of age:** Intervention group pre 93.1%, post 94.1%, Control group pre 94.5%, post 94.0%, Adj. Diff-in-Diff(ITT) 0.011, P>0.1  **Full vaccination by first birthday:** Intervention group pre 78.8%, post 78.1%, Control group pre 77.7%, post 75.8%, Adj. Diff-in-Diff(ITT) 0.009, P>0.1  **Home based care for maternal health (bednet use during pregnancy and early initiation of BF):** Adj. Diff-in-Diff(ITT) -0.012, P>0.1  **Facility based care for maternal health (tetanus toxoid vaccine, Vitamin A, ANC4+, ANC initiation, Skilled birth attendance, and PNC):** Adj. Diff-in-Diff(ITT) 0.085, P>0.1 |
| 39.  Garcia-Dia et al. (2016) The Philippines  [69] | **Design:** RCT  **Methods:** Socio-demographic data collected during recruitment using a questionnaire. Satisfaction questionnaire after child vaccination completed. Outcome data collected from medical records  **Setting:** Bago City health center  **Population:** Children between 12 and 15 months of age without MMR immunisation and parents  **Size:** Total=75, Intervention1=25, Intervention2=25, Control=25 | **Intervention 1:** Text message reminders for scheduled MMR vaccination sent 7-10days prior to appointment  **Intervention2:** Text message reminders with pictures for scheduled MMR vaccination sent 7-10days prior to appointment  **Control:** Routine care and verbal reminder of MMR vaccination | Unidirectional communication (text messages) | 1 | Vaccination appointment reminders | **^ Immunisation rates:** No significant difference found between the groups (P=0.07).  **^ Timeliness:** The two intervention groups performed better than control group in bringing their child for MMR immunisation on the date of the appointment or soon thereafter.  Scheduled date vs actual date of vaccination indicating delay was  0.96 days for intervention group1, 2.72 days for Intervention group2, and 20.64 days for control group. |
| 40.  Gerdts et al. (2020) Indonesia  [70] | **Design:** RCT  **Methods:** Baseline survey for demographic data and reproductive history. Online follow-up survey  **Setting:** Safe-abortion hotline in Indonesia  **Population:** Callers to the Samsara hotline seeking information about medication abortion.  **Size:** Total(pre/post)=300/242  Intervention=150/119, Control=150/123 | **Intervention:** As control + app with interactive step-by-step information on the process of medical abortion  **Control:** Empathic counselling on pregnancy options, support about unwanted pregnancies, protocols for medical abortion, warning signs of complications, care seeking, sexual and reproductive health. | Unidirectional communication | 1 | Educational information | **Preparedness, confidence, and support during the process of medical abortion:** no difference was detected in primary study outcomes between study arms. |
| 41.  Gibson et al. (2017) Kenya  [71] | **Design:** Cluster RCT (M-SIMU)  **Methods:** Baseline survey to collect Socio-demographic data at enrolment. Immunisation booklet screened at follow-up data collection at 12 months  **Setting:** Western rural Kenya, Siaya County, Nyanza Province, Gem and Asembo districts  **Population:** Caregivers of newborns (0-34days of age  **Size:** Total(villages)=152, 38 villages in each of the 4 arms.  Total(pre/post)=2018/1600  Arm1=489/360  Arm2=476/388  Arm3=562/446  Arm4=491/406 | **Arm2:** SMS reminder sent three days before scheduled vaccination (week 6, 10, and 14 for pentavalent, and 9 months for measles)  **Arm3:** Arm2 + 75 KES (85KES = USD$1)  **Arm4:** Arm2 + 200 KES  **Arm1: Control:** - | Unidirectional communication (text messages) | 1 | Immunisation appointment reminders | **^ Proportion of fully immunised children at 12 months(BCG, 3x polio vaccine, 3x pentavalent, and measles vaccine)**:  Control 82% vs Arm2 86%, RR 1.04 (95% CI 0.97 to 1.12) P=0.29  Control 82% vs Arm3 86%, RR 1.04 (95% CI 0.96 to 1.11) P=0.33  Control 82% vs Arm4 90%, RR 1.09 (95% CI 1.02 to 1.16) P=0.014  **Receiving all timely vaccinations:** Control 41%, arm2 48% (p=0.075), arm3 60% (p<0.0001), arm4 62% (p<0.0001)  **^ BCG, Pentavalent 1, 2, and 3, Polio 1, 2, and 3:** Proportion of children vaccinated with individual vaccinations ranged from 96% to 100% for all groups. No difference was observed.  **^ Measles:**  Control 84% vs. Arm2 87%, RR 1.04 (95% CI 0.97 to 1.11) P=0.28.  Control 84% vs. Arm3 87%, RR 1.03 (95% CI 0.97 to 1.10) P=0.36.  Control 84% vs. Arm4 90%, RR 1.07 (95% CI 1.01 to 1.14) P=0.034. |
| 42.  Gong et al. (2020) China  [72] | **Design:** Quasi-experimental study  **Methods:** Self-reported questionnaire at enrolment and follow-up 30-32 weeks of GA, incl. EDPS.  **Setting:** 3 public hospital in Jiangmen City, Guangdong Province (1 intervention, 2 control)  **Population:** Pregnant women <14 weeks of GA  **Size:** Total(pre/post)=4501/3336  Intervention=1739/1481, Control=2762/1855 | **Intervention:** Routine care + SMS based intervention with weekly text messages + reminders 1 day prior to ANC appointment + maternal school courses (psychology, exercise, diet and weight control)  **Control:** Routine care | Unidirectional communication (text messages) | 1 | Appointment reminders and educational information | **^ EPDS score early pregnancy:** Intervention group 6.2±4.3, Control group 6.0±4.4, P=0.3  **^ EPDS score late pregnancy:** Intervention group 3.9±3.9, Control group 5.2±4.3, P<0.001  **^ EPDS score early vs late pregnancy:** Intervention group pre 6.2±4.3, Intervention group post 3.9±3.9, P<0.001  **^ EPDS score early vs late pregnancy:** Control group pre 6.0±4.4, Control group post 5.2±4.3, P<0.001  **Association of depression with the intervention**: Intervention group Adj. OR 1.00, Control group Adj. OR 2.04 (95% CI 1.62 to 2.58) P<0.001 |
| 43.  Guo et al. (2019) China  [73] | **Design:** RCT  **Methods:** Data collected at each visit (blood glucose, weight)  **Setting:** Metabolic Disease Hospital of Tianjin Medical University  **Population:** Pregnant women diagnosed with GDM between week 24 and 28 of GA  **Size:** Total(pre)=124  Intervention=64, Control=60 | **Intervention:** Routine outpatient care + mHealth app for registration, self-measurements + online correspondence with study nurse  **Control:** Routine outpatient care (personalised dietary guidance, training plan, and performance) throughout pregnancy and follow-up 3 months postpartum | Bidirectional communication (chat) | 1 | Educational information (diet, exercise, blood glucose monitoring, insulin injection, etc.) | **^ Glycaemic control compliance:** Intervention group 83.3±12.5, Control group 70.4%±10.1, P<0.001  **Frequency of outpatient service:** Intervention group 8.1±1.3, Control group 11.2%±1.1, P<0.001  **Haemoglobin A1C before delivery:** Intervention group 4.7%±0.2, Control group 5.3%±0.3, P<0.001  *Other glycaemic measures reported*  **Vaginal delivery:** Intervention group 66.7%, Control group 70%, P=0.142  **Foetal macrosomia:** Intervention group 6.3%, Control group 10.0%, P=0.295  *Other delivery characteristics reported* |
| 44.  Hackett et al. (2018) Tanzania  [74] | **Design:** Cluster RCT (SUSTAIN application by World Vision)  **Methods:** 3weeks training period of CHWs + baseline survey. Postnatal household survey of new mothers  **Setting:** 32 Villages in Singida Region and Iramba district  **Population:** Community health workers (CHW) and pregnant women  **Size:** Total(CHW)=62  Intervention=31, Control=31  Total(mothers pre/post)=700/572  Intervention=371/304, Control=329/268 | **Intervention:** As control + implementation of Smartphone-based job aid application  **Control:** Supporting system to Improve Nutrition, Maternal, Newborn and Child Health implemented by World Vision | Unidirectional communication (data collection) | 1 | Prompt CHWs to counsel women, but the app is technically not part of it | **^ Facility delivery:** Intervention group 74%, Control group 62%, Adj. OR 1.96 (95% CI 1.21 to 3.19) P=0.01 |
|  |  |  |  | 4 | Data collection and record keeping during ANC, and delivery |  |
|  |  |  |  | 6 | Electronic “decision tree” protocols guiding counselling, referral, etc. |  |
|  |  |  |  | 8 | Home visit schedule |  |
| 45.  Harrington et al. (2019) Kenya [75] | **Design:** RCT (WACh XY)  **Methods:** Baseline questionnaire + questionnaire at 6 and 14 weeks postpartum  **Setting:** 2 public hospitals in the Kisumu and Siaya counties of western Kenya serving rural, low-income populations  **Population:** Pregnant women, >=28 weeks of GA, visiting antenatal clinics, Male partners were also invited to partake  **Size:** Total(pre/post)= 260/254  Intervention=130/125, Control=130/129 | **Intervention:** Routine care + automated text messages with actionable advice or question to promote dialogue once weekly from enrolment to 6 months postpartum  **Control:** Routine care | Bidirectional communication (chat) | 1 | Educational information (FP, contraception methods and their effectiveness, postpartum risk of pregnancy, contraceptive safety during lactation, side effects, misconceptions, al dual protection + BF and healthy pregnancy) | **^ Use of highly effective contraceptive 6 months postpartum:** Intervention group 69.9%, Control group 57.4%, Adj. RR 1.26 (95% CI 1.04 to 1.52) P=0.02.  **Use of highly effective contraceptive 6 weeks postpartum:** Intervention group 14.4%, Control group 18.8%, Adj. RR 0.86 (95% CI 0.45 to 1.65)  **Use of highly effective contraceptive 14 weeks postpartum:** Intervention group 51.3%, Control group 47.0%, Adj. RR 1.07 (95% CI 0.81 to 1.41)  **Overall contraceptive use at 6 weeks:** Unadjusted RR=1.00 (95% CI 0.60 to 1.66), Adjusted RR=1.19 (95% CI 1.01 to 1.41) P=0.04  **Overall contraceptive use at 14 weeks:** Unadjusted RR=1.12 (95% CI 0.89 to 1.41).  **Overall contraceptive use at 6 months:** Unadjusted RR=1.18 (95% CI 1.00 to 1.38). |
| 46.  Ilozumba et al. (2018) India [77] | **Design:** Quasi-experimental study  **Methods:** Questionnaire for collection of socio-demographic and outcome data.  **Setting:** Deoghar block, subdistrict in Jharkhand state, rural setting  **Population:** Women who had delivered in the last 12 months  **Size:** Total(women)=2200  Intervention= 728, Quasi-control=733, Control=739 | **Intervention:** As quasi-control + Mobile for mothers (MfM) program (ASHAs equipped with mobile phone (data collection tool / educational material)  **Quasi-Control**: As control + NGO existing intervention (maternal health education messages through dramas and dance and programs to increase male involvement)  **Control:** standard care by government programs | Unidirectional communication (materials and reminders, data collection) | 1 | Educational information through text, pictures, and voice prompts | **Maternal health knowledge (ANC visit, tetanus injections, folic acid supplements, delivery danger signs, “5 cleans” during delivery):** Intervention group vs control: Adj. OR 1.19 (95% 1.13 to 1.25) P=0.001. Intervention group vs quasi-control: Adj. OR 1.14 (95% 1.08 to 1.19) P=0.001.  **ANC attendance (>=4):** Intervention group vs control: Adj. OR 1.38 (95% 1.32 to 1.44) P=0.001. Intervention group vs quasi-control: Adj. OR 1.22 (95% 1.17 to 1.28) P=0.001.  **Delivery at a health facility:** Intervention group vs control: Adj. OR 1.35 (95% 1.29 to 1.42) P=0.001. Intervention group vs quasi-control: Adj. OR 1.18 (95% 1.12 to 1.24) P>0.05. |
|  |  |  |  | 4 | Data collection on site (registrations forms) |  |
|  |  |  |  | 6 | Decision support in forms of checklists, danger signs monitoring |  |
|  |  |  |  | 8 | Schedule reminders for ASHA to visit client at home |  |
| 47.  Jerin et al. (2020) Bangladesh  [78] | **Design:** Quasi-experimental study  **Methods:** Socio-demographic data collected at enrolment. Delivery information collected from medical records. Outcome data collected via structured questionnaires.  **Setting:** Secondary level health facility northwest of Dhaka  **Population:** Young infants ages 0-180 days of age  **Size:** Total(pre/post)=293/265  Intervention=164/151, Control=129/114 | One day training for senior HC staff and 3 days training for nurses and research assistants.  **Intervention:** Early initiation of BF supported by nurse + assessment of position and attachment + tailor made support. After discharge: biweekly telephone follow-up until 6 months of age. Lactation consultant available per need.  **Control:** Early initiation of BF supported by nurse | Bidirectional communication (phone call) | 1 | Education of and support to breastfeeding women | **^EBF rate:** Intervention group 78%, Control group 58%, P=0.000.  **EBF at month 0:** Intervention group 89%, Control group 85%  **EBF at month 1:** Intervention group 89%, Control group 77%  **EBF at month 2:** Intervention group 76%, Control group 62%  **EBF at month 3:** Intervention group 78%, Control group 67%  **EBF at month 4:** Intervention group 68%, Control group 29%  **EBF at month 5:** Intervention group 71%, Control group 42% |
| 48-49.  Jiang et al. (2014, 2019) China  [79, 80] | **Design:** Quasi-experimental study  **Methods:** Outcome data collected by the child’s health physicians during routine visits and subsequently extracted by researcher from health records at 4, 6, 12, and 24 months.  **Setting:** Community health centres in Urban settings  **Population:** Pregnant women at < 13 weeks of gestation  **Size:** Total(pre/post for primary BF outcome)=582/549  Intervention=281/265, Control=301/284  Total(primary anthropometric outcome)=467, Intervention=243, Control=224 | **Intervention:** Routine antenatal and prenatal care + one weekly content-specific messages from 28 weeks of gestation to 12 months postpartum + continuous communication between participant and research team on pregnancy/infant status (delivery date, feeding practices, work situation, introduction of solid food)  **Control:** Routine antenatal and postnatal care | Unidirectional communication (text messages)  Bidirectional communication (Phone calls) | 1 | Educational information (content: breastfeeding recommendations and infant feeding advise) and communication between research team and participants | **^ Median duration of EBF:** Intervention group 11.41 weeks (95% CI 10.25 to 12.57), Control group 8.87 weeks (95% CI 7.84 to 9.89), P<0.001.  Compared to the control group, the hazard ratio for stopping EBF in the intervention group was 0.80 (95% CI 0.66 to 0.97).  **EBF at 4 months:** Intervention group 46.4%, Control group 39.9%, OR 1.4 (95% CI 0.98 to 2.0).  **EBF at 6 months:** Intervention group 15.1%, Control group 6.3%, OR 2.67 (95% CI 1.45 to 4.91).  **Other feeding practices was reported.**  **^ BMI at 12 months (mean±SE):** Intervention group 17.04kg/m2±0.08, Control group 14.04kg/m2±0.08  No difference observed at 24 months.  **^ WLZ at 12 months (mean±SE):** Intervention group 0.44kg/m2±0.05, Control group 0.44kg/m2±0.05  No difference observed at 24 months. |
| 50.  Johri et al. (2020) India  [81] | **Design:** Cluster RCT  **Methods:** Face-to-face surveys, project administrative records, and interactive voice response system used to collected baseline and follow-up data.  **Setting:** Villages in Bawan block, Hardoi, UP.  **Population:** Primary caregivers of children between 0 and 12 months.  **Size:** Total caregivers/children(pre)=391/398  Intervention=185/188, Control=206/210  Total caregivers(post)=322  Intervention=154, Control=168 | **Intervention:** Face-to-face intervention in communities + educational audio and voice reminders for immunisation sessions + community mobilisation activities  **Control: -** | Unidirectional communication (audio, text messages)  Bidirectional communication (phone call with live expert)  Multi-directional communication | 1 | Informational education (healthcare entitlements, prevention, recognition, and management of IDs (diarrhoea, pneumonia, dengue, chikungunya) nutrition, WASH) and immunisation appointment reminders | **^ Immunisation schedule knowledge:** Intervention group pre 26.6%, Control group pre 34.6%, P=0.095. Intervention group post 66.7%, Control group post 44.6%, P<0.001, Impact of intervention Adj. OR 8.40 (95% CI 2.05 to 34.35).  *More immunisation knowledge outcomes reported alongside knowledge on infectious disease.* |
| 51.  Karamolahi et al. (2021) Iran [82] | **Design:** RCT  **Methods:** Questionnaire for demographic information and the Maternal Health literacy and pregnancy (MHLAP) at baseline and 8 weeks later  **Setting:**  **Population:** Pregnant women 6-12 weeks of GA  **Size:** Total(pre/post)=140/113  Intervention=70/60, Control=70/53 | **Intervention:** Training app + QA phone calls regarding the app + weekly app reminders + ability to contact researcher  **Control:** - | Unidirectional communication | 1 | Educational information (changes and adaptations of the body during pregnancy, foetus development, pregnancy care, tests, vaccinations, nutrition, taking supplements, personal hygiene, pregnancy and neonatal risk factors, neonatal care, bathing and BF, vitamin taking, baby position, and coverage) | **Health literacy score (mean±SD):**  Intervention group pre 45.35±6.75, post 58.03±6.57, P<0.001.  Control group pre 42.02±7.41, post 42.23±8.47, P=0.609.  Intervention group pre 45.35±6.75, Control group pre 42.02±7.41, P=0.014.  Intervention group post 58.03±6.57, Control group post 42.23±8.47, P<0.001. |
| 52.  Kassaye et al. (2016) Kenya  [83] | **Design:** Cluster RCT  **Methods:** Structured interviews at four visits (enrolment, 36 weeks of gestation, 7 days postpartum, and 6-8 weeks postpartum)  **Setting:** Hospitals, health centers and dispensaries within Homa Bay and Rachuonyo districts in Nyanza province Ntotal=26 (13 health facilities for each trial arm).  **Population:** Women identified with HIV during ANC visits less than 32 weeks of gestation and not receiving antiretroviral therapy. Male partners were also invited to participate  **Size:** Total(pre/post)=550/503  Intervention=280/261, Control=270/242 | **Intervention:** Participants were able to communicate with CHW by phone/text and received text messages (reminders, motivation, adherence support). Male participants were encouraged to partake in delivery planning and received health massages for mother and infant, warning signs, and nutrition  **Intervention and Control:** Community health workers retrained in PMTCT service and delivery, including appropriate messaging to women during labour | Unidirectional communication (text messages)  Bidirectional communication (phone call/text between participants and CHW) | 1 | Health information and reminders via SMS | **^ Antiretroviral uptake at 34-36 weeks of gestation**: Intervention group 97.3%, Control group 99.6%, RR 1.01 (95% CI 0.91 to 1.18). Uptake of antiretrovirals was high in both group and no difference were detected.  **Antiretroviral uptake at delivery:** Intervention group 94.7%, Control group 100%, RR 1.01 (95% CI 0.88 to 1.16).  **Facility delivery:** Intervention group 50.6%, Control group 59.5%.  **Infant uptake of antiretrovirals at delivery:** Intervention group 80.9%, Control group 90.1%.  **Infant uptake of antiretrovirals at 6 weeks postpartum:** Intervention group 99.6%, Control group 97.8%.  **Infant HIV testing:** Intervention group 88%, Control group 88.6%.  **HIV transmission:** Intervention group 0.4%, Control group 1.4%. |
|  |  |  |  | 9 | PMTCT training of CHW |  |
| 53.  Kawakatsu et al. (2020) Nigeria [84] | **Design:** RCT  **Methods:** Data collection at health clinic visits and via mobile application.  **Setting:** 33 Primary HC in Lagos  **Population:** Infants (vaccination scheme), pregnant women (ANC), couples/women (family planning)  **Size:** Total(infants)=8337  Intervention=4893, Control=3444  Total(pregnant women)=3441  Intervention=915, Control=2526  Total(Family planning)=397  Intervention=114, Control=283 | **Intervention:** As control + Reminder sent two days prior to scheduled appointment (vaccination, ANC, or Family planning). Another reminder was sent 7 days after scheduled appointment if missed  **Control:** Informed verbally and in written form about subsequent appointments by health workers. Follow-up phone call were made if appointments were missed | Unidirectional communication (text messages)  Bidirectional communication (follow-up calls) | 1 | Appointment reminders | **^ Vaccination attendance:** Intervention group 67%, Control group 62%, Adj. OR 1.17 (95% CI 1.05 to 1.31) P=0.005.  **Cumulative timeliness of vaccine (day0):** Intervention group 41.7%, Control group 35.7%, P<0.001. **(day7):** Intervention group 52.0%, Control group 46.2%, P<0.001. **(day14):** Intervention group 53.4%, Control group 48.3%, P<0.001. **(day30):** Intervention group 59.4%, Control group 54.5%, P<0.001.  **(3 months):** Intervention group 67.0%, Control group 62.4%, P<0.001.  **^ Antenatal care attendance:** Intervention group 55%, Control group 56%, Adj. OR 1.00 (95% CI 0.83 to 1.20) P=0.998.  **Cumulative timeliness of ANC:** No difference between intervention and control group at any time points.  **^ Family planning attendance:** Intervention group 29%, Control group 27%, Adj. OR 1.44 (95% CI 0.74 to 2.79) P=0.276.  **Cumulative timeliness of family planning:** No difference between intervention and control group at any time points. |
| 54.  Kazi et al. (2018) Pakistan [85] | **Design:** RCT  **Methods:** Socio-demographic data collected using baseline questionnaire. Second interview conducted at 18 weeks of age during household visit to collect outcome data. EPI immunisation cards were also inspected  **Setting:** Urban squatter settlement area, Ibrahim Haidry union council in Karachi  **Population:** Children less than 2 years of age and their parent/caretaker  **Size:** Total(pre/post)=300/168  Intervention=150/88, Control=150/79 | **Intervention:** As control + four standard EPI reminder SMS for Routine immunisation scheduled at week 6, 10, and 14 weeks of life.  **Control:** One-time counselling at the baseline survey regarding time of EPI vaccinations. | Unidirectional communication (text messages) | 1 | Vaccination appointment reminder | **^ Proportion of children immunized up to date at 18 weeks of age**  **BCG and polio before 18week**  **Pentavalent1 at 6w (ITT):** Intervention group 76%, Control group 71.3%, P=0.36. **Pentavalent1 at 6w (PP):** Intervention group 96%, Control group 86.4%, P=0.03. **Pentavalent2 at 10w (ITT):** Intervention group 58.7%, Control group 52.7%, P=0.31. **Pentavalent2 at 10w (PP):** Intervention group 78%, Control group 75.5%, P=0.69. **Pentavalent3 at w14 (ITT):** Intervention group 31.3%, Control group 26.0%, P=0.31. **Pentavalent3 at w14 (PP):** Intervention group 58%, Control group 51%, P=0.36  *PP: exclusion of children who died or migrated before 18 weeks of age, vaccination not confirmed through EPI cards, SMS not received.* |
| 55.  Kebaya et al. (2021) Kenya [86] | **Design:** RCT  **Methods:** Demographic and biomedical data collected at enrolment. Outcome data collected at follow-up visits at 6 and 6 weeks postpartum  **Setting:** 3 health facilities in Kisumu, Western Kenya (Jaramogi Oginga Odinga Teaching and referral Hospital, Kisumu East District Hospital and Lumumba Health Center)  **Population:** HIV positive women who had a live birth in the previous 24h  **Size:** Total=150  Intervention=75, Control=75 | **Intervention:** Routine care + PMTCT reminders call every second Monday until infant was 10 weeks old reinforcing key messages (medication, EBF, EID, scheduled immunisations, overall health)  **Control:** Routine care and free to call researcher on their own initiative | Bidirectional communication (phone calls) | 1 | Reminders on PMTCT messages | **^ Retention in care at 6 weeks (attending clinic as scheduled):** Intervention group 78.7%, Control group 58.7%, OR 2.6 (95% CI 1.3 to 5.3) P=0.009.  **Retention in care at 10 weeks:** Intervention group 69.3%, Control group 37.3%, OR 3.8 (95% CI 1.9 to 7.5) P<0.001.  **Adherence to NVP prophylaxis at 6 weeks:** Intervention group 90.7%, Control group 72%, OR 3.8 (95% CI 1.5 to 9.5) P=0.005.  **Early infant diagnosis (PCR taken at 6 weeks):** Intervention group 70.7%, Control group 58.7%, OR 1.7 (95% CI 0.9 to 3.3)  **Early infant diagnosis (PCR taken at 10 weeks):** Intervention group 72%, Control group 61.3%, OR 1.6 (95% CI 0.8 to 3.2)  **EBF at 10 weeks:** Intervention group 97%, Control group 90.7%, OR 3.8 (95% CI 0.8 to 18.7) P=0.1. |
| 56.  Kebede et al. (2019) Ethiopia [87] | **Design:** Cluster RCT  **Methods:** Recorded patient data  **Setting:** 16 health centres in urban settings  **Population:** Mothers discharged from delivery wards postpartum  **Size:** Total=342,  Intervention=173, Control=169 | **Intervention group:** received the SMS (short message service) or a voice call reminder at 48 and 24 hours before the due postnatal appointment.  **Control group:** Usual appointment notification provided at the time of birth | Unidirectional communication (text messages or voice call) | 1 | Postnatal care appointment reminders | **^ Postnatal care attendance:** Intervention fully complied 5%, Intervention partially complied 27%, Control fully complied 3%, Control partially complied 19%. Adj. OR 2.98 (95% CI 1.51 to 5.81) P=0.005. |
| 57.  Khodabandeh et al. (2017) Iran  [88] | **Design:** RCT  **Methods:** Socio-demographic and obstetric questionnaire at baseline. Health-related questionnaire completed 2 and 6 weeks postpartum. IPAQ and FFQ conducted post-intervention.  **Setting:** Two teaching hospitals affiliated with Tabriz University of Medical Sciences (Al-Zahra and Taleghani)  **Population:** Primiparous mothers admitted to postpartum units after delivery  **Size:** Total(pre/post)=220/206  Intervention=112/105, Control=108/101 | **Intervention:** Face-to-face healthy lifestyle education delivered before discharge + educational booklets (WHO) + phone number to address any issues/questions for 6 weeks postpartum + education messages sent every 5 days  **Control:** Routine care and trainings of the postpartum ward | Unidirectional communication (text messages)  Bidirectional communication (phone calls if made) | 1 | Healthy lifestyle education (physical activity, proper diet, mental-personal health, prevention of problems | **^ Physical activity:**  Walking: Intervention group 83.8%, Control group 91.1%, Moderate physical activity: Intervention group 15.2%, Control group 7.9%, Vigorous physical activity: Intervention group 1%, Control group 1%. P=0.121.  **^ Diet:** no difference found between the two groups regarding consumption of different food groups (bread/cereal, meat/beans, Vegetables, Fruits).  Intake of milk and dairy were observed to be slightly higher in the intervention group compared to control group, P=0.041.  Consumption of iron tables were greater in intervention group compared to control group at week 2 (P=0.013) and week 6 (P=0.002). |
| 58.  Khorshid et al. (2014) Iran  [89] | **Design:** RCT  **Methods:** Survey interviews (Socio-demographic collection) and blood sample analysis  **Setting:** 10 urban public health centers  **Population:** Pregnant women from 14-16 weeks of gestation  **Size:** Total(pre/post)=116/93  Intervention=58/49, Control=58/44 | **Intervention:** Routine care + daily iron supplementation reminders and educational information (week 16 + 12 weeks) regarding absorption, side effects, function  **Control:** Routine care | Unidirectional communication (text messages) | 1 | Antenatal care appointment reminder and educational information | **^ Compliance with intake of iron supplements**:  High compliance: intervention group 94%, Control group 66%. Moderate compliance: Intervention group 4%, Control group 18%. Low compliance: Intervention group 2%, Control group 16%, P=0.003.  After 12 weeks the mean number of iron tablets taken in the intervention and control group was 80.5 and 67.2 respectively, P<0.001.  **Measures of blood indices of anaemia after intervention:** Haemoglobin (mean g/dL): Intervention group 11.2 ± 0.5, Control group 11.2 ± 0.9, P=0.960.  Haematocrit (mean): Intervention group 33.9 ±1.7, Control group 34.0 ±2.6, P=0.670**.**  Ferritin in ng/dL: Intervention group 24.4 ± 35.0, Control group 22.5 ± 19.7, P=0.630. |
| 59.  Kiani et al. (2021) Iran  [90] | **Design:** Quasi-experimental study  **Methods:** Questionnaire to collect baseline and outcome data  **Setting:** Isfahan, Falavarjan County, Center of Iran country  **Population:** Pregnant women 16-20 weeks of GA participating in the childbirth preparation classes  **Size:** Total(pre/post)=110/93  Intervention=58/49, Control=52/44 | **Intervention:** Routine care + access to educational app for 3 months  **Control:** Routine care | Unidirectional communication (videos, written material) | 1 | Educational information (physical activity description, mental health benefits of exercise in pregnancy, proper types of exercise during pregnancy, way of doing daily activities, planning exercise, time to stop, massage, stretching, relaxation, exercise demonstrations, education videos, etc.) | **^ Physical activity level (MET mean±SD):**  Intervention group pre 1570.00±754.11, post 1872.44±653.86, P=0.014.  Control group pre 1637.27±812.65, post 1413.29±758.74, P=0.084.  Intervention group pre 1570.00±754.11, control group pre 1637.27±812.65, P=0.680.  Intervention group post 1872.44±653.86, control group post 1413.29±758.74, P=0.002.  **^ Perceived benefits(1), perceived barriers(2), perceived enjoyment(3), and perceived social support(4)** was also assessed across time and between groups. No difference was observed between groups at baseline. A significant change was observed in the Intervention group for 1, 2, 3, and 4 (P=<0.001) and in the control group for 1 (P=<0.001) and 4 (P=0.005). |
| 60.  Kinuthia et al. (2021) Kenya  [91] | **Design:** 3-arm nonblinded RCT  **Methods:** Socio-demographic data collected using questionnaire at enrolment (ANC clinic).  Outcome data was collected at 6 w postpartum, and 6, 12, 18, 24 months postpartum including patient blood samples, infant dried blood spots. Viral load results, appointments, deliveries, clinic visits, medication refill was abstracted from clinical records.  **Setting:** 6 public MCH clinics in Kenya: 2 in peri-urban Nairobi County and 4 in rural Siaya, Kisumu, and Homa Bay Counties  **Population:** Peripartum women living with HIV (WLWH)  **Size:** Total(pre/completed 24 months)=824/737  Arm1=271/239, Arm2=276/249, Control=277/249 | **Arm1(one-way):** Received weekly automated SMS from enrolment (ANC clinic) until 2y postpartum.  Visit reminder sent 3 days ahead of appointment and 3 and 6 days after if missed.  **Arm2(Bidirectional):** Arm1 + received questions related to message topic to promote engagement + ability to send SMS to nurses as needed.  **Control:** Routine care | Unidirectional communication (text messages)  Bidirectional communication (SMS interaction between participant and nurse) | 1 | Appointment reminders and educational information regarding ART adherence, infant HIV prophylaxis, pregnancy education, birth preparedness, pregnancy and delivery complications, infant health family planning. | **^ Maternal virologic non-suppression:** Arm1 vs Control group overall follow-up time, Adj. RR 1.02 (95% CI 0.67 to 1.54) P=0.94.  Arm2 vs Control overall follow-up time, Adj. RR 0.80 (95% CI 0.52 to 1.23) P=0.31.  Cumulative incidence of virologic non-suppression by 24 months did not differ between groups:  Arm1 23.3%, Arm2 15.6%, Control group 19.9%.  Arm1 vs. Control Adj. HR 1.17 (95% CI 0.83 to 1.65) P=0.38.  Arm2 vs. Control Adj. HR 1.78 (95% CI 0.53 to 1.15) P=0.21.  **^ On-time visit attendance up to 24 months postpartum:**  Arm1 vs Control Adj. RR 1.00 (95% CI 0.98 to 1.03) P=0.81.  Arm2 vs Control Adj. RR 1.01 (95% CI 0.99 to 1.04) P= 0.36.  No difference found in number of scheduled visits and attendance was the same for all groups  **^ Loss to follow-up:** No difference between groups  **^ Infant HIV infection and death:** 7 Infants acquired HIV and 38 Infants diet by 24 months postpartum.  Arm1 vs Control Adj. HR 0.83 (95% CI 0.37 to 1.87) P=0.66.  Arm2 vs Control Adj. HR 1.44 (95% CI 0.71 to 2.92) P=0.31.  **Maternal HIV adherence and ART resistance:** No difference  **Post-partum contraception:** Arm1 58.0% vs Control group 53.3%, Adj. HR 1.09 (95% Ci 0.84 to 1.42) P= 0.51.  Arm2 58.9% vs Control group 53.3%, Adj. HR 1.31 (95% CI 1.01 to 1.70) P=0.04.  **EBF at 6 months:** Arm1 81.6%, Arm2 79.6%, Control group 79.3% |
| 61.  Klokkenga et al. (2019) Ghana  [92] | **Design:** Cluster RCT  **Methods:** Socio-economic, obstetric characteristics, delivery outcomes and treatment was collected during admission  **Setting:** 15 hospitals or large polyclinics in Greater Accra Region  **Population:** Midwifes  **Size:** Total(pre/post)=147/146  Intervention=71/70, Control=76/76  (3311 deliveries) | **Intervention:** Safe delivery app – a EmONC training tool with visual guidance and clinical instructions  **Control:** | Unidirectional communication | 6 | Decision making tool for midwives | **Postpartum haemorrhage (PPH):** Intervention group 15%, Control group 16%, OR 0.86 (95% CI 0.59 to 1.25)  **Mean relative difference in blood loss:** 5.3% (95% CI -3.2% to 13.8) |
|  |  |  |  | 9 | Educational training videos (active management of 3^rd^ stage of labour, manual removal of the placenta, treatment of post-partum haemorrhage) |  |
| 62.  Lau et al. (2014) South Africa  [93] | **Design:** RCT  **Methods:** Quantitative questionnaire at baseline and endline; qualitative FGDs for intervention group  **Setting:** Urban primary healthcare facility  **Population:** Pregnant women >= 18 years of age  **Size:** Total(pre/post)=206/118  Intervention=102/57, Control=104/61 | **Intervention:** Routine care + Antenatal health information according to the week of pregnancy ranging from three messages per week to daily messages  **Control:** Routine care | Unidirectional communication (text messages) | 1 | Educational information | **^ No statistically significant difference detected between the control and intervention group in any of the nine knowledge questions at exit (all p > 0.05).** |
| 63.  Levine et al. (2021) Ghana  [94] | **Design:** Cluster RCT  **Methods:** CHV enrolled women/caregiver recently given birth. CHV took photos of vaccination cards at follow-ups. Endline survey to assess before and after vaccination coverage  **Setting:** 15 Communities in Northern Ghana, rural Karaga district  **Population:** C Caregivers and infants  **Size:** Total(caregivers)=666  Arm A=217, Arm B=203, Control=246  Total(infants)=690  Arm A=223, Arm B=211, Control=256  Total(Community Health Volunteer (CHV))=2-4 per intervention community | **Intervention A:** Voice call reminder from study staff. Up to three attempts were made to contact caregivers in order to keep up with vaccination schedule.  **Intervention B:** CHV encourage caregivers to get vaccinations (dates, times and locations). CHV free to follow-up to their discretion. Caregivers and CHV incentivized with rewards (1Ghana cedis (0.5USD) for on-time vaccination  **Control:** Routine health services | Bidirectional communication (Study staff call to caregiver) | 1 | Vaccination reminder + Educational information highlighting the importance of early vaccination | **^ On-time completion of early vaccinations (1x polio vaccination + BCG with 28days of life):** Arm A: Pre 25%, post 38%. Arm B: Pre 13%, post 55%. Control: Pre 44%, post 49%.  Arm A vs Control: 10.5 percentage point difference (95% CI 4.0 to 17.1).  Arm B vs Control: 49.5 percentage point difference (95% CI 26.4 to 72.5).  **1x polio vaccination (on-time):**  Arm A: Pre 28%, post 42% **(any time):** Pre 36%, post 45%  Arm B: Pre 13%, post 56% **(any time):** Pre 32%, post 70%  Control: Pre 51%, post 53% **(any time):** Pre 63%, post 61%  **BCG vaccination (on-time):**  Arm A: Pre 63%, post 73% **(any time):** Pre 97%, post 95%  Arm B: Pre 53%, post 82% **(any time):** Pre 94%, post 98%  Control: Pre 71%, post 78% **(any time):** Pre 90%, post 91% |
|  |  |  |  | 3 | Birth registration |  |
|  |  |  |  | 7 | Communication between study staff and local health workers to keep caregivers informed on timely vaccinations, dates, times, locations etc. |  |
| 64.  Li et al. (2020) China  [95] | **Design:** RCT  **Methods:** Socio-demographic and health data collected at baseline. Outcome data collected at baseline and 49 days postpartum via structured questionnaire  **Setting:** 3 MCHs in Chengdu city, Sichuan Province  **Population:** Pregnant women <13 weeks of GA  **Size:** Total(pre/post)=1400  WeChat(WC)=350/347, Specialist Team(ST)=350/348, WeChat plus Specialist Team(WC-ST)=350/348  Control=350/344 | **WC:** Routine care + WeChat with health educational information about maternal health care every 2 weeks  **ST:** Routine care + follow-up with specialists via telephone >=5 times during ANC and 1 time postpartum + reminding pregnant women to come for ANC, provide delivery information and handling of high-risk pregnancies  **WC-ST:** WC + ST  **Control:** Routine care | Unidirectional communication  (WC)  Bidirectional communication (ST) | 1 | Educational information | **Satisfaction rate of participants for the service received at 49 days postpartum(pre):** WC 80.35%, ST 79.99%, WC-ST 81.49%, Control 81.99%, P=0.981  **^ Satisfaction rate of participants for the service received at 49 days postpartum(post):** WC 91.64%, ST 95.16%, WC-ST 98.58%, Control 85.18%, P<0.001  **Rates of early pregnancy care:** WC 90.02%, ST 94.68%, WC-ST 97.77%, Control 87.23%, P<0.001  **Maternal system management (number of women who attended the service package):** WC 82.73%, ST 92.07%, WC-ST 96.33%, Control 79.91%, P<0.001  **Postpartum visit:** WC 94.97%, ST 98.86%, WC-ST 100%, Control 91.33%, P<0.001 |
| 65-67.  Lund et al. (2012, 2014a, 2014b) Tanzania  [96-98] | **Design:** Cluster RCT  **Methods:** Socio-demographic data collected using structured questionnaires at enrolment and 6 weeks postpartum. Outcome data retrieved from patient health records  **Setting:** General population, Primary health care facilities in rural and urban settings  **Population:** Pregnant women at first prenatal care attendance  **Size:** Total(pre/post)=2637/2550  Intervention=1351/1311, Control=1286/1239 | **Intervention:** Routine care + Mobile phone vouchers (access to emergency obstetric care and referral mechanism) + SMS reminders and educational information. Two messages per month before week 36 and two messages per week after week 36  **Control:** Routine care | Unidirectional communication (text messages)  Bidirectional communication (Free call voucher system) | 1 | Antenatal appointment reminders and educational information on a wide range of pregnancy related topics | **^ Skilled delivery attendance:** Intervention group 60%, Control group 47%. Urban setting: Intervention group 82%, Control group 50%, OR 5.73 (95% CI 1.51 to 21.81). Rural Settings: Intervention group 43%, Control group 44%, OR 0.85 (95% CI 0.42 to 1.71). **^ Number of women receiving four or more antenatal care visits:** Intervention group 44%, Control group 31%, OR 2.39 (95% CI 1.03 to 5.55).  **Tetanus toxoid vaccination at first antenatal care visit:** Intervention group 96%, Control group 94%, OR 1.58 (95% CI 0.41 to 6.01). **Tetanus toxoid vaccination at least 4 weeks after first antenatal care visit:** Intervention group 72%, Control group 56%, OR 1.62 (95% CI 0.81 to 3.26).  **Intermittent preventive treatment in pregnancy at first prenatal visit:** Intervention group 91%, Control group 86%, OR 1.10 (95% CI 0.35 to 3.43).  **Intermittent preventive treatment in pregnancy at least 4 weeks after first prenatal visit:** Intervention group 65%, Control group 52%, OR 1.97 (95% CI 0.98 to 3.94).  **Gestational age 36 or more at last antenatal care visit:** Intervention group 28%, Control group 20%, OR 1.48 (95% CI 0.89 to 2.45). **Antepartum referral:** Intervention group 10%, Control group 5%, OR 1.66 (95% CI 0.68 to 4.06).  **Stillbirth:** Intervention group 17 per 1000 births, Control group 26 per 1000 births, OR 0.65 (95% CI 0.34 to 1.24).  **Perinatal mortality:** Intervention group 19 per 1000 births, Control group 36 per 1000 births, OR 0.50 (95% CI 0.27 to 0.93). **Death of child <42 days after birth:** Intervention group 14 per 1000 births, Control group 15 per 1000 births, OR 0.79 (95% CI 0.36 to 1.74). |
| 68.  Lund et al. (2016) Ethiopia  [99] | **Design:** Cluster RCT (SDA)  **Methods:** demographic structured questionnaires. Outcome data collected at baseline and 6 + 12 months post intervention  **Setting:** 70 HF from 5 districts in the West Wollega Zone, Oromiya Region (35 HF in each arm).  **Population:** Healthcare workers and Pregnant women  **Size:** Total(pre)=176 HCW,  3601 pregnant women, Intervention=1627, Control=1974  Total(post)=130 HCW,  3139 pregnant women, Intervention=1474, Control=1665 | **Intervention:** Training of HCWs on how to manage obstetric and neonatal emergencies via the SDA intervention using visual guidance in animated videos and voiceover. The SDA also contains a catalogue with obstetric drugs and equipment  **Control:** HCW provided standard care | Unidirectional communication (videos) | 6 | Electronic decision support | **^ Perinatal death (rate per 1000 total births):**  Intervention group 14, Control group 23, Adj. OR 0.76 (95% CI 0.32 to 1.81).  **Neonatal resuscitation - knowledge score at 6 months(mean±SD):** Intervention group 5.92±2.02, Control group 4.30±1.45, Adj. OR 1.67 (95% CI 1.02 to 2.32).  **Neonatal resuscitation - skills score at 6 months(mean±SD):** Intervention group 12.92±5.92, Control group 7.50±4.91, Adj. OR 6.04 (95% CI 4.26 to 7.82).  **Neonatal resuscitation - knowledge score at 12 months mean±SD):** Intervention group 5.53±1.61, Control group 4.09±1.50, Adj. OR 1.54 (95% CI 0.98 to 2.09)**.**  **Neonatal resuscitation - skills score at 12 months (mean±SD):** Intervention group 16.22±6.06, Control group 8.20±4.72, Adj. OR 8.79 (95% CI 7.14 to 10.45). |
|  |  |  |  | 9 | Training of healthcare workers |  |
| 69.  Martinez-Fernandez et al. (2015) Guatemala  [101] | **Design:** Quasi-experimental study  **Methods:** Data collection from official information published by the local authority  **Setting:** Rural communities within Alta Verapaz (Department serving 1.2 million people)  **Population:** 125 Volunteer community facilitators (CF) serving 466 rural communities  **Size:** Intervention=20(2008), 40(2009), 60(2010), 125(2011), Control=? | **Intervention:** community facilitators provided with tele-equipment and trained in voice communication and data + use of an audio-conference system. Basic training in vital sign monitoring and identification of signs of distress in children and pregnant women.  **Control: -** | Bidirectional communication (community facilitators and medical staff) + emergency transfer of patients by ambulance | 3 | Tracking number of deaths | **^ Maternal Mortality Ratio:** Intervention group 309 (2008), 515 (2009), 388 (2010), 178 (2011), 254 (2012) Control group 338 (2008), 418 (2009), 378 (2010), 366 (2011), 558 (2012).  **^ Infant Mortality Rate:** Intervention group 25 (2008), 19 (2009), 12 (2010), 14 (2011), 13 (2012) Control group 27 (2008), 22 (2009), 33 (2010), 25 (2011), 20 (2012). |
|  |  |  |  | 4 | Collecting data on site on pregnant women and infants |  |
|  |  |  |  | 7 | CF could request specialist consultation on case management and logistic support in emergency situations |  |
|  |  |  |  | 9 | Face to face session for training of CFs in clinical issues |  |
| 70.  Maslowsky et al. (2016) Ecuador [102] | **Design:** Quasi-experimental study  **Methods:** Clinical characteristics and demographic data collected at baseline. Follow-up 3 months postpartum (via telephone) for collection of outcome data.  **Setting:** Large public hospital and a community clinic with a birthing center, Southern Quito  **Population:** Inpatient mothers after delivery  **Size:** Total(pre/post)=178/135  Intervention=102/75, Control=76/60 | **Intervention:** As control + educational session by nurse within 24hours after discharge(information) + nurse on-call during the first 30days postpartum  **Control:** Routine care (follow-up 1 and 6 weeks postpartum + initiation of FP) | Bidirectional communication(patient/nurse) | 1 | Educational information applicable to mother or child (Feeding, environment, infections disease symptoms, recommended appointments, infections, contraception and more) | **^ EBF 3 months postpartum:** Intervention group 86.7%, Control group 66.7%, P=0.005  **^ Infant formula use 3 months postpartum:** Intervention group 12%, Control group 30%, P=0.016  **^ Infant attended newborn check-up (1 week postpartum):** Intervention group 72%, Control group 53.3%, P=0.022  **^ Infant attended newborn check-up (6 week postpartum):** Intervention group 98.7%, Control group 98.3%, P>0.999  **^ Use of contraception at 3 months postpartum:** Intervention group 76%, Control group 68.3%, P=0.871 |
| 71.  Masoi et al. (2019) Tanzania  [103] | **Design:** Quasi-experimental study  **Methods:** Quantitative questionnaire  **Setting:** Two health facilities (Health center and regional referral hospital) in urban settings for intervention group. Control group collected from other facilities within the same municipal  **Population**: Pregnant women who began ANC at less than 20 weeks of gestation. Controls were matched by age, education level, and gravidity (number of previous pregnancies)  **Size:** Total=450,  Intervention=150, Control=300 | **Intervention:** Routine care + interactive messaging alert system receiving health education messages regarding pregnancy.  **Control:** routine care | Bidirectional communication (text messages and phone calls) | 1 | Educational information shared with participants. Participants could call or SMS healthcare staff with questions | **^ Knowledge on obstetric and newborn danger signs:** Intervention group pre 46.0%, Control group pre 44.7%, No difference found between groups.  Intervention group post 77.3%, Control group post 48%, difference in mean score 9.53 ± 2.67 vs 6.52 ± 4.30, p<0.001.  **^ Preparedness practices:** Intervention group pre 20%, control group pre 17%, no difference found between groups. Intervention group post 70.7%, Control group post 29.7%, Difference in mean score 4.17 ± 1.37 vs 2.63 ± 1.78, p<0.001. |
| 72-73.  Modi et al. (2015, 2019)  India  [104, 105] | **Design:** Cluster RCT (iMoMCARE)  **Methods:** Quantitative household surveys. Qualitative KII, IDIs, FGDs  **Setting:** 22 PHCs, predominantly tribal/rural area  **Population:** Health care staff (mainly ASHAs) and pregnant women  **Size:** 11 Intervention PHCs and 11 control PHCs serving approx. 22.000 each  Total(ASHAs)=561,  Intervention=280, Control=281  Total(pregnant women)=6493  Intervention=3328, Control=3165 | **Intervention and control:** three-day MNCH refresher training and Routine implementation of ASHA program.  **Intervention:** Four-day training and mentoring of ASHAs and PHC staff for use of ImTeCHO mobile phone and web application respectively. Ongoing technology support and facilitation. SEWA helpline counsellor supporting ASHAs management of complicated cases | Bidirectional communication (phone calls, SEWA rural helpline, data collection) | 1 | Education information. | **^ At least two home visits by ASHA within the first week after delivery – ITT analysis (mean):** Intervention group 32.4 (95% CI 29.7 to 35.1), Control group 22.9 (95% CI 20.2 to 25.6), Adj. effect size 10.2 (95% CI 6.4 to 14.0) P<0.001.  **^ At least two home visits by ASHA within the first week after delivery – PP analysis (mean):** Intervention group 39.7 (95% CI 36.2 to 43.3), Control group 27.6 (95% CI 23.9 to 31.2), Adj. effect size 12.2 (95% CI 7.2 to 17.5) P<0.001.  **^ MACCI – ITT analysis (mean):** Intervention group 43.0 (95% CI 39.7 to 46.3), Control group 38.5 (95% CI 34.5 to 41.6), Adj. effect size 4.9 (95% CI 0.2 to 9.5) P=0.037.  **^ MACCI – PP analysis (mean):** Intervention group 44.2 (95% CI 40.9 to 47.5), Control group 39.1 (95% CI 35.8 to 42.4), Adj. effect size 5.3 (95% CI 0.7 to 10.0) P=0.024. |
|  |  |  |  | 3 | Record viral events |  |
|  |  |  |  | 4 | Patient data collection at site |  |
|  |  |  |  | 5 | Data transfer to PHC |  |
|  |  |  |  | 6 | Decision support feature in app. |  |
|  |  |  |  | 7 | Phone calls between providers |  |
|  |  |  |  | 8 | Daily updated work schedule |  |
|  |  |  |  | 10 | Tracking ASHAs performance |  |
|  |  |  |  | 11 | Manage inventory |  |
| 74.  Mohamadirizi et al. (2014) Iran  [106] | **Design:** Quasi-experimental study  **Methods:** Sociodemographic data and fertility characteristics collected via questionnaire at baseline. Follow-up questionnaire for outcome data.  **Setting:** Navab Safavi Healthcare center  **Population:** 1^st^ time pregnant women, 6-12 weeks of GA  **Size:** Total(pre/post)=100  Intervention=50, Control=50 | **Intervention:** Prenatal electronic education  **Control:** Prenatal educational booklet. Questions were answered via phone calls | Unidirectional communication | 1 | Educational information (mental and physical health, nutrition and medication, place of natural delivery, BF and FP counselling, neonatal care, etc.) | **Prenatal care awareness score before intervention (mean±SD):** Intervention group 29.21±7.4, Control group 30.21±6.4, P=0.234  **Prenatal care awareness score 4 weeks after intervention (mean±SD):** Intervention group 44.74±3.4, Control group 40.74±6.4, P=0.001.  Intervention group pre vs. post: P=0.001  Control group pre vs post: P=0.001 |
| 75-76.  Murthy et al. (2019, 2020) India  [107, 108] | **Design:** Quasi-experimental study (mMitra)  **Methods:** House to house data collection using survey at baseline, after delivery, and 1 year postpartum.  **Setting:** Urban slums of Mumbai  **Population:** Pregnant women  **Size:** Total(pre/post)=2016/1417  Intervention=1516/1038, Control=500/379 | **Intervention:** Routine care + mMitra voice messages (145) delivered twice weekly according to GA during pregnancy, once weekly first week postpartum, then twice weekly until 1 year postpartum + free call-back service within 2 days after original call was received to hear voice message again  **Control:** Routine care | Unidirectional communication (voice messages) | 1 | Educational information on maternal and child health | **^ Full-term infants born >= 2.5kg**  OR 1.334 (95% CI 0.983 to 1.839) P=0.064.  **Malnourishment at 1 years of age (weight for age):**  OR 0.823 (95% CI 0.590 to 1.147) P=0.249.  **Full immunisation (1 dose of BCG, 3 doses of Pentavalent, 3 doses of polio, 1 dose of measles):**  OR 1.485 (95% CI 1.112 to 1.984) P=0.007.  **Early initiation of BF:** Adj. OR 0.86 (95% CI 0.67 to 1.1) P=0.23. **Colostrum feeding:** Adj. OR 1.29 (95% CI 0.86-1.94) P= 0.20.  **>=3 ANC visits:** Adj. OR 1.508 (95% CI 0.797 to 2.853) P=0.207. **Maternal TT injection:** Adj. OR 1.596 (95% CI 1.05 to 2.85) P=0.028. **Calcium suppl.:** Adj. OR 1.218 (95% CI 0.943 to 1.574) P=0.131. **Iron/folic acid suppl.:** Adj. OR 0.905 (95% CI 0.710 to 1.153) P=0.419.  **Facility delivery:** Adj. OR 2.543 (95% CI 1.488 to 4.348) |
| 77.  Nagar et al. (2018) India  [109] | **Design:** Cluster RCT  **Methods:** Data collection at immunisations camps, exit survey after completion for DTP series  **Setting:** Rural regions of Udaipur, Rajasthan  **Population:** Mothers and infants less than 6 months old  **Size:** Total(village)=96, 32 in each group  Total(post)=198  Arm1=61, Arm2=75, Control=62  5 lost to follow-up. | **Control:** Near field communication sticker placed on existing immunisation card (serves as digital record)  **Arm 1:** Child wearing pendent (necklace) that can be scanned and give access to health records where immunisation records are digitally stored.  **Arm 2:** Arm1 + voice call reminder the day before and on the day of the vaccination camp + messages if camp was missed. | Unidirectional communication (text messages and automated voice calls) | 1 | Automated voice call reminders for scheduled vaccinations | **^ Received 3 DTP shorts within 2 consecutive immunisation camps:** Arm 1 67.2%, Arm 2 69.3%, Control 74.2%, P=0.684.  **^ Received 3 DTP shorts within 2 months from the time of registration:** Arm 1 37.7%, Arm 2 38.7%, Control 27.4%, P=0.332.  **^ Received 3 DTP shorts before 180 days after birth:**  Arm 1 57.4%, Arm 2 58.7%, Control 69.4%, P=0.315. |
|  |  |  |  | 4 | Data collection on site uploaded and synced with database |  |
|  |  |  |  | 5 | Health records (immunisation history) are retrievable for viewing in the field |  |
| 78.  Nemerimana et al. (2021) Rwanda  [110] | **Design:** Quasi-experimental study  **Methods:** Data extracted from medical records  **Setting:** Paediatric development clinics (PDCs) at two rural district hospitals, Rwinkwavu and Kirehe, and 7 HC in same catchment areas  **Population:** Infants enrolled in PDCs  **Size:** Total(pre/post)=1917/1612  Intervention=1051/912, Control=866/700 | **Intervention:** mHealth tool with decision support and algorithms for growth monitoring by plotting child’s anthropometric z-scores  **Control:** Paper-based chart for child growth monitoring and for nutritional status assessment and age 2 and 6 months | Unidirectional communication | 4 | Data collection | **^ Length for age z-score (stunting):** Intervention group pre 79%, post 82%, Control group pre 77%, post 57%. P<0.001.  **^ Weight for Length z-score (wasting):** Intervention group pre 78%, post 90%, Control group pre 76%, post 59%. P<0.001.  **^ Weight for age z-score (underweight):** Intervention group pre 79%, post 93%, Control group pre 77%, post 67%. P<0.001. |
| 79.  Ngoc et al. (2014) Vietnam  [111] | **Design:** RCT  **Methods:** Interviews, bimanual examination and transvaginal ultrasonography, semiquantitative pregnancy test and questionnaire  **Setting:** 4 hospitals in Vietnam  **Population:** Women seeking early medical abortion  **Size:** Total(pre/post)=1433/1433  Intervention=713/709, Control=720/662 | **Intervention:** Phone follow-up + semiquantitative pregnancy test and self-administered questionnaire to assess abortion status.  **Control:** clinic follow-up (standard care) including ultrasonographic examination to confirm abortion status | Bidirectional communication (phone call from nurse to participants 2 weeks after mifepristone administration) | 2 | Self-administered semiquantitative pregnancy kit for home testing | **^ Rate of complete abortion:** Intervention 94.8%, Control 94.6%, RR 1.002 (95% CI 0.977 to 1.028)  **^ Rate of ongoing pregnancy:** Intervention 2.5%, Control 2.7%, RR 0.934 (95% CI 0.985 to 1.019). 84.7% of intervention group were not referred to clinic - only one pregnancy missed in this group. Of the 106 (about 15%) women referred to clinic, 13 were experiencing ongoing an pregnancy. |
| 80.  Nguyet et al. (2021) Vietnam  [112] | **Design:** Quasi-experimental study  **Methods:** Baseline Questionnaire (24-72h postpartum), post-test data collected 4 weeks postpartum during home visit  **Setting:** Hue University of Medicine and Pharmacy’s Hospital,  **Population:** First time mothers, inpatients for 3 days after vaginal delivery  **Size:** Total(pre/post)=70/52  Intervention=35/25, Control=35/27 | **Intervention:** Routine care + bedside education on BF, sleep and activities, environment and safety, hygiene, and managing newborn health via tablet. Same contents were downloaded to mothers smartphone and given as PDF for home study. Questions and self-study were encouraged.  **Control:** Routine care | Unidirectional communication (multimedia content on tablet/smartphone) | 1 | Educational material on newborn care | **^ EBF at the pre-test 24-72h postpartum:** Intervention group 14.8%, Control group 12%, P=0.477.  **^ EBF 4 weeks postpartum:** Intervention group 74.1%, Control group 44%, P=0.027.  **Maternal confidence at the pre-test 24-72h postpartum and 4 weeks postpartum (mean±SD):** Intervention group pre 106.00 ± 24.07, Intervention group post 138.37 ± 18.36, Difference -32.37 (95% CI -41.76 to -22.98) P<0.001.  Control group pre 103.04 ± 20.64, Control group post 111.60 ± 22.74, Difference -8.56 (95% CI -15.27 to -2.85) P=0.015. |
| 81.  Nordberg et al. (2021) Kenya  [113] | **Design:** RCT (WelTel PMTCT)  **Methods:** Questionnaire (baseline, 6 months and 24 months), outcome data collection from medical records and logs by healthcare workers.  **Setting:** Six antenatal care clinics across western Kenya serving both urban and rural communities  **Population:** HIV-positive pregnant women living with the HIV-exposed infant enrolled at their first ANC visit  **Size:** Total=600,  Intervention=299, Control=301 | **Intervention:** Routine PMTCT care + weekly "How are you?" text messages send to participants until 24 months postpartum. If no response or problems was reported, healthcare worker contacted participant by phone.  **Control:** Routine PMTCT care | Bidirectional communication (text messages or phone calls) | 1 | Supportive text messages and follow-up | **Early infant diagnosis (EID) HIV testing by 8 weeks postpartum:** Intervention 71.2%, Control 71.8%, Adj. RR 1.00 (95% CI 0.93 to 1.09) P=0.95. |
| 82.  Odeny et al. (2014) Kenya  [114] | **Design:** RCT  **Methods:** Socio-demographic data collected during recruitment and outcome data from clinic records after intervention  **Setting:** Mix of rural and urban antenatal care or HIV clinics in five health facilities  **Population**: Pregnant HIV positive women at least 18 years of age, between 28 weeks of gestation and delivery  **Size**: Total(pre/post)=388/381(women),  Intervention=195/194, Control=193/187  Intervention(post)=187(infant) Control(post)=181(infant) | **Intervention group:** Routine care + up to 14 text messages from week 28 to delivery and weekly for 6 weeks postpartum  **Control group:** Routine care | Bidirectional communication. Both intervention and control group were allowed to call study nurse | 1 | Congratulations, advise and encouragements to contact healthcare staff if any questions regarding pregnancy, delivery or postnatal care arose. | **^ Postpartum retention in PMTCT:** Intervention group 19.6%, Control group 11.8%, RR 1.66 (95% CI 1.02 to 2.70) P=0.04. Women in SMS arm had significantly higher probability of attending clinics within 8 weeks, compared to those in control arm.  **^ Infant HIV testing**: Intervention group 92.0%, Control group 85.1%, RR 1.08 (95% CI 1.00 to 1.16) P=0.04.  The probability of infant HIV testing within 8 weeks was significantly higher in the SMS group than in the control group. |
| 83.  Odeny et al. (2019) Kenya  [115]  *Different period of data collection, but same intervention used as for Odeny et al. 2014* | **Design:** Cluster RCT, stepped-wedge trial  **Methods:** Data abstracted from routine clinic records, HEI follow-up register, HEI cards, and laboratory registers.  **Setting:** Public health facilities supported by the Family AIDS Care and Educational Services program to provide PMTCT services in Kisumu, Migori, and Homa Bay  **Population:** All HIV-positive pregnant women enrolled in the PMTCT program  **Size:** Total(infants)=2326  Intervention=1613, Control=713  Total(women)=2472  Intervention=1725, Control=747 | **Intervention:** Routine care + up to 14 text messages during pregnancy and postpartum + ability to call or text a nurse to address any questions/concerns. A study nurse would call participant to ascertain delivery status for women not giving birth in the facility.  **Control:** Routine care | Bidirectional communication | 1 | Educational information, advice and encouragement | **^ HIV virologic testing (within 8weeks):** Intervention group 90.9%, Control group 85.4%, Adj. RR 1.03 (95% CI 0.97 to 1.10) P=0.3  **^ Proportion of women retained in postpartum care:** Intervention group 90%, Control group 76%, Adj. RR 1.12 (95% CI 0.97 to 1.30) P=0.1 |
| 84.  Oladepo et al. (2021) Nigeria  [116] | **Design:** Quasi-experimental study  **Methods:** Semi-structured questionnaire administered at baseline and endline + observational checklist to document child immunisation cards  **Setting:** 6 states representing geo-political zones, 14 LGAs  **Population:** Mothers and infants aged 0-2 months attending immunisation clinics in the Primary HCs  **Size:** Total(pre)=3500  Total(post)=3440 | **Intervention:** As control + messages sent 3 times a week for 10 months focusing on next routine immunisation appointment, benefits of keeping appointments, benefits of timely and full immunisation, consequences of refusal/non-compliance  **Control:** Flyers on the importance of adequate child nutrition and growth monitoring + clinic health talks about vaccinations | Unidirectional communication (text messages) | 1 | Vaccination appointment reminders and education information | **^ BCG:** Intervention group 59.6%, Control group 41.1%, P<0.000  **^ Pentavalent 1:** Intervention group 86.9%, Control group 80.7%, P<0.000. **^ Pentavalent 2:** Intervention group 87.5%, Control group 74.8%, P<0.000. **^ Pentavalent 3:** Intervention group 85.0%, Control group 70.6%, P<0.000.  **Oral polio 0:** Intervention group 78.2%, Control group 71.2%, P<0.000. **Oral polio 1:** Intervention group 87.5%, Control group 81.2%, P<0.000. **Oral polio 2:** Intervention group 87.2%, Control group 77.2%, P<0.000. **Oral polio 3:** Intervention group 85.4%, Control group 75.7%, P<0.000.  **HBV 0:** Intervention group 44.0%, Control group 33.1%, P<0.000.  **IPV:** Intervention group 86.8%, Control group 76.4%, P<0.000.  **Measles:** Intervention group 55.3%, Control group 26.8%, P<0.000.  **Yellow fewer:** Intervention group 75.9%, Control group 23.9%, P<0.000. |
| 85.  Olajubu et al. (2020) Nigeria  [117] | **Design:** Quasi-experimental study  **Methods:** Socio-demographic data collected at enrolment using structured questionnaire. Outcome data collected during 4 PNC visits.  **Setting:** Primary healthcare facilities from 6 LGAs in Osun State, Southwest Nigeria  **Population:** Women registered for ANC at selected PHCs (9 for intervention, 8 for control) with GA between 28and34 weeks  **Size:** Total(pre/post)=380/339  Intervention=190/175, Control=190/164 | **Intervention:** Routine care + baseline education on WHO recommended number of visits and timing + 2-3 automated text messages per week from week 35 GA until 6 weeks postpartum. Reminders were sent 1 day prior to the 4 scheduled appointments  **Control:** Routine care + baseline education on WHO recommended number of visits and timing | Unidirectional communication (text messages) | 1 | Postnatal care appointment reminders and educational information (healthy prenatal lifestyle practices, medical signs for seeking clinical care, recommended number and timing of PNC visits etc.) | **^ Four PNC visits:** Intervention group 30.9%, Control group 3.7%, Adj. OR 10.869 (95% CI 4.479 to 26.374) P<0.001.  **PNC 1 (0-1 day postpartum):** Intervention group 76.6%, Control group 39.6%, Adj. OR 5.122 (95% CI 3.142 to 8.347) P<0.001.  **PNC 2 (3 days postpartum):** Intervention group 44.6%, Control group 7.9%, Adj. OR 9.261 (95% CI 4.794 to 17.888) P<0.001.  **PNC 3 (7-14 days postpartum):** Intervention group 38.3%, Control group 7.9%, Adj. OR 6.215 (95% CI 3.226 to 11.974) P<0.001.  **PNC 4 (6^th^ week postpartum):** Intervention group 85.1%, Control group 65.9%, Adj. OR 2.749 (95% CI 1.595 to 4.738) P<0.001. |
| 86.  Oliveira-Ciabati et al. (2017) Brazil  [118] | **Design:** Cluster RCT  **Methods:** Data collected from ANC card, medical records and interviews using structured questionnaires  **Setting:** 20 Primary health care units and 4 maternity hospitals  **Population:** Pregnant women with GA <=20 weeks  **Size:** Total=  Intervention(all)=770, Intervention(PRENACEL)=116, Control=440 | **Intervention:** Routine ANC + weekly set of short text messages according to GA + ability to send questions and comments related to ANC to healthcare workers (HCW) in the research team  **Control:** Routine ANC | Unidirectional communication (automated text messages)  Bidirectional communication (texting between pregnant woman and HCW) | 1 | Education information (physiology of pregnancy and childbirth, elements of ANC, postpartum care and contraception, psychosocial aspects of pregnancy and the postpartum period) | **^ Proportion of women with high ANC score (>=42 points):**  Intervention(All) 88.2%, Control 83.0%, Adj. RR 1.05 (95 % CI 1.00 to 1.09)  **>=6 ANC(ITT):** Intervention(All) 89.1%, Control 84.8%, P= 0.06.  **>=6 ANC(PP):** Intervention(PRENACEL) 96.6%, Control 84.8%, P= 0.01.  *List of recommended ANC practices assessed included various immunisations, micronutrient supplementation, HIV testing, etc.* |
| 87.  Omole et al. (2016) Nigeria [119] | **Design:** Cluster RCT  **Methods:** Baseline questionnaire for socio-demographic data + reproductive and ANC attendance history. Follow-up data collection on place of delivery.  **Setting:** 4 secondary health facilities in Ife-Ijesa are, Osun State  **Population:** Pregnant women in first trimester  **Size:** Total=508,  Intervention=260, Control=248 | **Intervention:** Weekly pregnancy-related health text messages according to GA and reminder for ANC appointments + option to ask questions via SMS  **Control:** General health text messages | Unidirectional communication (text messages)  Bidirectional communication (chat via text messages) | 1 | Appointment reminders and educational information (birth preparedness, complications, nutrition, HIV testing, BF, postnatal care, baby care, FP, immunisation) | **Delivery in Health facility:** Intervention group pre 55%, post 0.84%, increase 29%.  Control group pre 81%, post 94%, increase 13% |
| 88.  Onono et al. (2019) Kenya [120] | **Design:** Quasi-experimental study  **Methods:** Data was derived from CMNH registers compiled by CHWs during the routine service delivery.  **Setting:** East Rachuonyo, Homa Bay County, Rural Western Kenya  **Population:** Pregnant women =< 28week of GA  **Size:** Total=1176  Intervention=350, Control=826 | **Intervention:** Routine care + mAccess intervention where pregnant women receive weekly text messages until 6 weeks postpartum. Women are prompt to ask questions from a live nurse (m-convo). Women using m-convo were eligible for National Health Insurance Fund. Women could text “mHelp” if she had questions, felt ill, or in labour. Transport would be arranged to take her to facility where she would be expected.  **Control:** Routine care | Bidirectional communication (SMS between nurse and pregnant women) | 1 | Educational information | **^ Number of ANC visits (>=4):** Intervention group 91.14%, Control group 67.68%, Adj. OR 4.8 (95% CI 3.20 to 7.09)  **^ Number of PNC visits (>=4):**  Intervention group 64.60%, Control group 30.60%, Adj. OR 4.10 (95% CI 3.11 to 5.36)  **^ Pregnancy outcomes (Miscarriage/Stillbirth):** Intervention group 2.57%, Control group 2.0%, Adj. OR 1.97 (95% CI 0.81 to 4.81)  **^ Status of mother after birth (Dead):** Intervention group 0.29%, Control group 0.50%, Adj. OR 0.61 (95% CI 0.07 to 5.45)  **^ Time taken to reach facility:** <30min: Intervention group 36.86%, Control group 64.79%. 30-60min: Intervention group 51.14%, Control group 28.51%. >1h: Intervention group 12.00%, Control group 6.69%. |
| 89.  Pai et al. (2013) India  [121] | **Design:** RCT  **Methods:** Socio-demographic data collected at enrolment and blood sample analysis and structured interview at follow-up  **Setting:** Municipal General Hospital in low income urban Mumbai  **Population:** Low-income, anaemic pregnant women between 13 and 28 weeks of gestation  **Size:** Total(pre/post)=130/79  Intervention=65/39, Control=65/40 | **Intervention:** Short audio messages (phone) 3t/week for 3 months focusing on iron supplementation + counselling sessions and a free supply of medication  **Control**: Counselling session and a free supply of medication. | Unidirectional communication (audio messages) | 1 | Educational information | **^ Adherence to treatment (mean±SD):** Intervention group before 9.53 ± 0.99, Control group before 9.69 ± 0.98, Intervention group after 9.86 ± 1.06, Control group after 9.59 ± 1.06. No difference pre- and post-Hb levels within groups and no difference between groups post intervention. |
| 90.  Paratmanitya et al. (2021) Indonesia  [122] | **Design:** Cluster RCT  **Methods:** Demographic and anthropometric data collected at first home visit using a standardised. protocol. Outcome data collected throughout the preconception period (using structured questionnaire) and from medical records during pregnancy.  **Setting:** Three sub-districts of Bantul district, Yogyakarta  **Population:** Women planning for pregnancy  **Size:** 322 pre-conceptional women at baseline, of which 205 were later confirmed pregnant  Intervention=159, Control=163 | **Intervention:** Routine care + maternal mentoring from pre-conception until 12 weeks pregnant. Once a month women would be asked about signs of pregnancy. If there was any indication of pregnancy, a reminder for scheduling first ANC was sent to women.  **Control:** Routine care | Bidirectional communication (text messages between women and mentor) | 1 | Educational information, support, and reminders to be timely with HC visits and comply with iron supplementation recommendations. | **^ Timing of first ANC visit (mean±SD):** Intervention group 39.75 ± 7.71, Control group 41.96 ± 9.72, Adj. OR 2.58 (95% CI 1.30 to 5.11), P=0.02 (individual level). Intervention group 39.85 ± 7.55, Control group 41.77 ± 7.35, Adj. OR 3.00 (95% CI 1.17 to 7.72, P=0.073 (cluster level).  **^ Change in the level of preconception health knowledge:** Intervention group score 6.60 ± 0.93, Control group score 3.81 ± 1.15, P<0.05.  **^ Change in anthropometric measure (MUAC):** Intervention group 0.21 ± 0.09cm, Control group 0.07 ± 0.14cm, P>0.05.  **^ Change in anthropometric measure (Weight): I**ntervention group 1.33 ± 0.17kg, Control group 0.77 ± 0.14kg, P>.05. |
| 91.  Parsa et al. (2019) Iran  [123] | **Design:** Quasi-experimental study  **Methods:** Questionnaire pre and post intervention regarding pre-eclampsia knowledge  **Setting:** Obstetrician clinics in Kerman, Iran  **Population:** Pregnant women  **Size:** Total=108  Intervention=54, Control=54 | **Intervention:** access to pre-eclampsia knowledge app  **Control:** - | Unidirectional communication | 1 | Educational information (pre-eclampsia, risk factors, complications, and signs and symptoms) | **Pre-eclampsia knowledge score(mean±SD)**: Intervention group pre 14.84±17.55, Control group pre 14.56±17.55, P=0.94  Intervention group post 78.08±14.19, Control group post 15.75±19.49, P=0.00  Intervention group pre 14.84±17.55, Intervention group post 78.08±14.19, P=0.00  Control group pre 14.56±17.55, Control group post 15.75±19.49, P=0.04 |
| 92.  Prieto et al. (2017) Guatemala  [124] | **Design:** Quasi-experimental study  **Methods:** Interviews questions based on a 2008 national survey to assess knowledge and practices of breastfeeding  **Setting:** Health center in a rural area of San Juan Sacatepéquez, Guatemala  **Population:** Pregnant women 8 months pregnant or more or new mothers with a child less then 4 months old  **Size:** Total(pre/post)=100/78 Intervention1=24/20, Intervention2=32/24, Intervention3=30/22, Intervention4=14/12 | **Intervention1:** Text message twice a week related to newborn nutrition designed to promote recommended breastfeeding attitudes. **Intervention2:** participants assigned to peer to peer group chat of 10 individuals each to address issues/doubts.  **Intervention3:** intervention 1 and 2 + ability to communicate with health professionals via group chat, where healthcare professional would bring up topics and correct misinformation. **Intervention4(control):** encouraged to use phone as for matters related to their babies | Unidirectional communication (text messages).  Bidirectional communication (phone calls and group chat) | 1 | Educational information, addressing misinformation and answering questions | **^ Exclusive breastfeeding recommendations awareness:** Intervention group1 pre 40%, post 100%.  Intervention group2 pre 75%, post 50%.  Intervention group3 pre 45%, post 95%.  Intervention group4 (Control) pre 75%, post 67%.  Overall awareness increased from 58% to 93% (P<0.001) |
| 93.  Prinja et al. (2017) India  [125] | **Design:** Quasi-experimental study  **Methods:** Baseline data was collected from the annual health survey in 2011. Post-intervention data was collected from household data collected in 2015  **Setting:** Four community development blocks of Kaushambi district, a rural area of Uttar Pradesh. Two intervention blocks and two control blocks were matched on two health service utilisation indicators.  **Population:** Mothers with children <2 years of age  **Size:** Total(pre)=450 with children 29 days to 6 months of age. Intervention=225, Control=225. Total(pre)=310 mothers with children aged 12-23 months. Intervention=124, Control=186.  Total(post)=534 with children 29 days to 6 months of age. Total(post)=1019 mothers with children aged 12-23 months.  Total(ASHAs)=259 | **Intervention**: ASHAs used mHealth application as job aid to support client assessment, client counselling, early identification, treatment and/or rapid referral of pregnancy, postpartum and newborn complications  **Control:** - | Unidirectional communication (text messages reminders)  Bidirectional communication (between ASHAs and other health care providers + referrals system) | 1 | Audio and visual prompt to aid client counselling | Attributable to the intervention (Difference-in-difference parameters)  **^ Coverage of >=3 ANC visits:** Increased by 10.3%  **^ Coverage of <=2 tetanus toxoid vaccine**: Increased by 4.28%  **^ Full antenatal care: Increased by 1.05%.** The above changes were statistically insignificant.  **^ Iron-Folic acid supplementation:** Significantly increased by 12.58%.  There was an increase in **blood pressure** (0.7%) and **urine testing** (2.4%).  **Institutional deliveries** increased by 4.02%  **Ambulance usage** increased by 2.06%  **Weight measurements** and **blood test** significantly increased in both intervention and control group, however the increase was greater in the Control group leading to a (difference-in-difference parameter) decrease in weight taking (-10.7%) and blood testing (-5.7%).  Similarly, **Full immunisation** decreased (-6.4%) resulting from a greater increase in control group compared to intervention group.  Self-reporting of **illnesses/complication during pregnancy** increased by 13.11% and by 19.6% **after delivery.** |
|  |  |  |  | 5 | Antenatal/postnatal care documentation by ASHAs on site transferred to common server |  |
|  |  |  |  | 6 | Multi-media job aids to support ASHAs in their assessment and counselling of clients |  |
|  |  |  |  | 8 | Reminders to ASHA regarding upcoming appointments |  |
| 94.  Qureshi et al. (2020) Pakistan  [126]  *Data overlap with Von Dadelszen et al. (2020)* | **Design:** Cluster RCT (CLIP intervention)  **Methods:** data collection surveys carried out quarterly  **Setting:** Matiari (rural) and Hyderabad (semi-urban) districts, Sindh Province  **Population:** Married pregnant women  **Size:**  Total health workers=223  Total pregnancies(pre)=39424  Intervention=20238, Control=19186  Total pregnancies(post)=35791  Intervention=18441, Control=17350 | **Intervention:** Community engagement with leaders, Pregnant women, mothers, and other stakeholders to promote content regarding hypertension/ (pre-)eclampsia, and more + CLIP visits using the PIERS On the Move tool (POM) +  CHW-led CLIP antenatal contacts every 4 weeks (<28wks) and every 2 weeks (28-35wks) and once weekly (<35wks) + 4 times postpartum. Visits are guided by CLIP POM mHealth app  **Control:** Routine ANC | Unidirectional communication (data collection) | 3 | Maternal and Newborn Health Registry | **^ All-cause maternal mortality:** Intervention group 0.3%, Control group 0.3%, Adj. OR 1.08 (95% CI 0.69 to 1.71) P=0.74  **^ All-cause maternal morbidity:** Intervention group 10.9%, Control group 8.9%, Adj. OR 1.12 (95% CI 0.57 to 2.16) P=0.77  **^ All-cause perinatal mortality and late neonatal mortality:**  Intervention group 9.6%, Control group 9.9%, Adj. OR 0.95 (95% CI 0.86 to 1.03) P=0.22  **^ All-cause neonatal morbidity:**  Intervention group 11%, Control group 7.2%, Adj. OR 1.22 (95% CI 0.77 to 1.96) P=0.40  **Birth preparedness and complication readiness:** Intervention group 42.2%, Control group 29.1%, Adj. OR 2.41 (95% CI 0.67 to 8.67) P=0.077  **Delivery in facility with emergency care:** Intervention group 65.5%, Control group 68.3%, Adj. OR 0.9 (95% CI 0.60 to 1.34) P=0.482  **Proportion of facility births:** Intervention group 66.8%, Control group 66.3%, Adj. OR 0.96 (95% CI 0.71 to 1.29) P=0.695 |
|  |  |  |  | 4 | Blood pressure measurements and test for proteinuria |  |
|  |  |  |  | 5 | Data transfer from POM mobiles to central REDCap server |  |
|  |  |  |  | 6 | Electronic decision support with pictograms and visual prompts directing CHWs to recommend appropriate care |  |
| 95.  Rani et al. (2022) India  [127] | **Design:** RCT  **Methods:** Pregnancy physical activity Questionnaire (PPAQ) used at each visit  **Setting:** Maternity hospital OPD Haryana, India  **Population:** Pregnant women, 11-14 weeks of GA  **Size:** Total=60  12 women in each group (A-E) | **Group A:** Supervised exercise once weekly from 15^th^ week of GA -> delivery  **Group B:** Advised and encourages to improve level of activity  **Group C:** Group B + pedometer + 2 educational and motivational text messages weekly  **Group D:** Group C – Group B  **Group E:** Routine care | Unidirectional communication (text messages) | 1 | Educational and motivational information (general health and well-being, healthy nutrition, healthy behaviour, pregnancy related myths) | **^ Gestational weight gain – kg (mean±SD):** group A 11.71±1.97, group B 12.14±3.15, Group C 11.96±2.72, Group D 12.99±2.77, Group E 13.25±2.80, P=0.932  **^ 2 months postpartum weight – kg (mean±SD):** group A 61.92±6.30, group B 67.92±6.73, Group C 64.17±9.82, Group D 68.50±10.89, Group E 65.00±10.09, P=0.029  **^ 2 months postpartum weight retention – kg (mean±SD):** group A 5.54±2.17, group B 6.18±1.42, Group C 5.94±2.42, Group D 7.41±1.94, Group E 8.33±3.34, P=0.005 |
| 96.  Reiss et al. (2019) Bangladesh  [128] | **Design:** RCT  **Methods:** Baseline face-to-face interviews + follow-up calls 2 weeks and 4 months post MR  **Setting:** 41 rural, peri-urban and urban health facilities in Chittagong, Dhaka and Sylhet division  **Population:** Women who had had an MR procedure  **Size:** Total(pre/post)=969/772  Intervention=485/389, Control=484/383 | **Intervention:** As control + interactive voice messages for 4 months post MR  **Control:** FP counselling, offer of available contraception methods, access to paramedic-led reproductive health call center | Unidirectional communication (voice messages) | 1 | Educational information | **Self-reported LARC at 4 months post MR:** Intervention group 12%, Control group 15%, Adj. OR 1.06 (95% CI 0.53 to 2.13)  *List of secondary outcomes related to contraception use, subsequent MR or abortion, Intimate partner violence, etc.* |
| 97.  Ross et al. (2013) Thailand  [129] | **Design:** RCT  **Methods:** Quantitative questionnaire and depression score; qualitative IDIs  **Setting:** Prenatal clinic  **Population:** HIV positive pregnant women with GA<=28 weeks  **Size:** Total=40,  Intervention=20, Control=20 | **Intervention:** Routine care + weekly Informational and emotional support; possibility of calling a registered nurse/midwife  **Control:** Routine care | Bidirectional communication (Phone call) | 1 | Educational information and support | **^ Depression rates** of 72.5% at baseline (55% (minor depression) + 17.5% (clinical depression)) among HIV-infected pregnant women in the control and intervention groups.  **^ Minor Depression score** among intervention group decrease over time. (T1: 13 to T3: 8), but not for control group (T1: 9 to T3: 11) |
| 98.  Ruton et al. (2018) Rwanda  [130] | **Design:** Implementation evaluation study /Quasi-experimental study (RapidSMS)  **Methods:** Data collected from database on messages sent by CHWs and the Rwanda Health Management Information System  **Setting:** 10 out of 30 districts in Rwanda with poor maternal and child health indicators, rural districts furthest from capital, Kigali.  **Population:** CHWs and Pregnant women/mothers  **Size:** Total(CHW)= >45.000  Total(mothers)=>2.5 million | **Intervention:** RapidSMS system for collection of routine health information by CHWs regarding maternal and child health. UNICEF provided more comprehensive support in selected districts.  **Control:** RapidSMS system for collection of routine health information by CHWs regarding maternal and child health. | Bidirectional communication (RapidSMS between healthcare workers) | 4 | Data collection on site | **^ ANC visits:** Rates of attendance increased across the study period, but no change in either the level or trend in both supported and non-supported districts  **^ Health facility deliveries:** Declining rate of health facility deliveries consistent with decline in fertility. RapidSMS did not change the level of facility delivery, but an increase in trend was observed in supported districts. No change observed in non-supported districts.  **^ PNC visits:** In supported district RapidSMS was associated with 0.11 visits/1000 catchment population increase in the level of PNC visits, P=0.007. No change observed in non-supported districts.  **^ Malnutrition screening:** small decrease in trend in supported districts, P<0.001. Otherwise, no change observed. |
|  |  |  |  | 5 | Data transfer to server |  |
|  |  |  |  | 7 | Communication between CHW and healthcare workers |  |
|  |  |  |  | 8 | Appointment reminders sent to CHWs who would then follow-up with pregnant women/mothers |  |
| 99.  Sabin et al. (2020) Uganda  [131] | **Design:** RCT  **Methods:** data wireless pill monitor (WPM) recorded data and time of each opening  **Setting:** 2 Government operated ANC hospitals, Entebbe Grade B Hospital in Wakiso district (urban) and Mityana District Hospital (rural)  **Population:** HIV positive pregnant women, 12-26 weeks of GA  **Size:** Total(pre/post)=165/133  Intervention=84/66, Control=81/67 | **Intervention:** As control + text message reminder (chosen by participant) triggered after 120minutes after scheduled dose time if WPM had not been opened (until 3 months postpartum) + monthly support and counsel at clinic visits  **Control:** Standard care + provided with a WPM for HIV medication and instructed on its use. | Unidirectional communication (text messages) | 1 | Medication reminders | **^ Full retention (attended all scheduled visits ANC/PNC, collected ART medication at each visit, delivered at study hospital):** Intervention group 49.3%, Control group 53.1%, P(ITT)=0.66. Intervention group 66.7%, Control group 69%, P(PP)=0.86.  **Retention in care pre-delivery:** Intervention group 80.6%, Control group 85.9%, P(ITT)=0.31. Intervention group 95.8%, Control group 96.6%, P(PP)=0.89  **Retention in care post-delivery:** Intervention group 59.7%, Control group 67.2%, P(ITT)=0.38. Intervention group 75%, Control group 82.8%, P(PP)=0.50  **Delivery at study hospital:** Intervention group 89.6%, Control group 81.3%, P(ITT)=0.18. Intervention group 87.5%, Control group 82.8%, P(PP)=0.78 |
| 100.  Sarmiento et al. (2019) The Philippines  [132] | **Design:** RCT  **Methods:** data collected from hospital records and via interviews  **Setting:** The Philippine General Hospital, Manila  **Population:** Women admitted for delivery who were referred to co-management of GDM  **Size:** Total(pre/post)=308/61  Intervention=154/31, Control=154/30 | **Intervention:** As control + SMS reminders twice/week  **Control:** Routine care consisting of 10-minute lecture on postpartum GDM care and a 75g OGTT request form + instructions on timing of testing 6-12weeks postpartum. | Unidirectional communication (text messages) | 1 | Reminders and educational information (GDM postpartum care) | **^ GDM follow up:** Intervention group 19.5%, Control group 20.1%, Adj. OR 0.98 (95% CI 0.63 to 1.52) P=0.932  Postpartum glucose status assessment provided with proportion of women with 1) normal blood glucose, 2) Impaired fasting glucose, 3) impaired glucose tolerance, 4) Diabetes Mellitus |
| 101.  Schwartz et al. (2015) South Africa  [133] | **Design:** Quasi-experimental study  **Methods:** Baseline and postpartum (6-8 weeks) data collection semi-structured interviews. Other outcome data retrieved from medical records  **Setting:** Witkoppen Health and Welfare Center, non-governmental primary health clinic, Johannesburg  **Population:** HIV positive pregnant women <=36 weeks of GA on highly-active antiretroviral therapy (HAART)  **Size:** Total(pre/post)=100/98  Intervention=50/48, Control=50 | **Intervention:** Case-manager sent weekly pre-scripted text messages to participants until 6-8 weeks postpartum + one pre-delivery and two post-delivery phone calls to discuss delivery plans and postpartum care. Participants could request additional calls from CM as needed  **Control:** Routine care | Unidirectional communication (text messages)  Bidirectional communication (calls) | 1 | Appointment reminders and educational information (motivational support, pregnancy and infant-related health information) | **^ At least 1 ARV pick-up after delivery:** Intervention group 92%, Control group 90%, P=0.73  **^ Actively engaged in HIV care at 10 weeks:** Intervention group 94%, Control group 96%, P=0.65  **^ ART retention at 12-months post-delivery:** Intervention group 78%, Control group 76%, P=0.71  **^ Infant received PCR at 6 weeks:** Intervention group 76%, Control group 44.9%, P<0.01  **^ Infant received PCR at 10 weeks:** Intervention group 90%, Control group 63.3%, P<0.01 |
| 102.  Seth et al. (2018) India  [134] | **Design:** RCT  **Methods:** demographic information collected at enrolment  **Setting:** rural communities in Mewat region, state of Haryana  **Population:** Children 24 months or younger  **Size:** Total(pre/post)=608/204 Intervention1=201/188, Intervention2=203/179,  Control=204/182 | **Intervention 1:** Automated SMS reminders  **Intervention 2:** Automated SMS reminders with compliance linked incentives (mobile phone talk time = USD $0.50 per immunisation)  **Control:** Self-return based on written immunisation records and verbal notification at follow-up visits | Unidirectional communication (text messages) | 1 | Vaccination appointment reminders | **^ Immunisation coverage**:  Intervention1 pre 40% (95% CI 37 to 43), post 40.1% (95% CI 30.8 to 69.2). Intervention2 pre 40% (95% CI 37 to 43), post 50.0% (95% CI 30.8 to 76.9). Control pre 39% (95% CI 36 to 42), post 41.7% (95% CI 23.1 to 69.2)  Intervention 1 vs Control: Adj. RR 1.02 (95% CI 0.94 to 1.11) P=0.64  Intervention 2 vs Control: Adj. RR 1.09 (95% CI 1.002 to 1.18) P=0.04  **Timeliness of vaccinations(within 14 days after scheduled date):** Intervention1 24%, Intervention2 40.8%, Control 31.3%, P<0.03 |
| 103.  Sevene et al. (2020) Mozambique [135]  *Data overlap with Von Dadelszen et al. (2020)* | **Design:** Cluster RCT (CLIP intervention)  **Methods:** data collection surveys carried out quarterly  **Setting:** 4 Primary health centers in Maputo province and 8 from Gaza province, Southern Mozambique  **Population:** Married pregnant women  **Size:**  Total health workers=50  Total pregnancies(pre)=15123/13180  Intervention=7931/6941, Control=7192/6239 | **Intervention:** Community engagement with leaders, Pregnant women, mothers, and other stakeholders to promote content regarding hypertension/(pre-)eclampsia, and more + CLIP visits using the PIERS On the Move tool (POM) +  CHW-led CLIP antenatal contacts every 4 weeks (<28wks) and every 2 weeks (28-35wks) and once weekly (<35wks) + 4 times postpartum. Visits are guided by CLIP POM mhealth app  **Control:** Routine ANC | Unidirectional communication (data collection) | 3 | Maternal and Newborn Health Registry | **^ All-cause maternal mortality:** Intervention group 0.2%, Control group 0.1%, Adj. OR 1.98 (95% CI 0.60 to 6.56) P=0.26  **^ All-cause maternal morbidity:** Intervention group 9.2%, Control group 9.6%, Adj. OR 1.35 (95% CI 0.59 to 3.10) P=0.48  **^ All-cause perinatal mortality and late neonatal mortality:**  Intervention group 5.2%, Control group 4.6%, Adj. OR 1.23 (95% CI 0.99 to 1.54) P=0.06  **^ All-cause neonatal morbidity:**  Intervention group 3.1%, Control group 4.5%, Adj. OR 0.99 (95% CI 0.32 to 3.07) P=0.98  **Birth preparedness and complication readiness:** Intervention group 43.7%, Control group 49.6%, Adj. OR 0.91 (95% CI 0.47 to 1.77) P=0.72  **Delivery in facility with emergency care:** Intervention group 11.3%, Control group 13.0%, Adj. OR 0.85 (95% CI 0.27 to 2.62) P=0.70  **Proportion of facility births:** Intervention group 67.3%, Control group 74.2%, Adj. OR 0.85 (95% CI 0.28 to 2.61) P=0.71 |
|  |  |  |  | 4 | Blood pressure measurements and test for proteinuria |  |
|  |  |  |  | 5 | Data transfer from POM mobiles to central REDCap server |  |
|  |  |  |  | 6 | Electronic decision support with pictograms and visual prompts directing CHWs to recommend appropriate care |  |
| 104.  Seyyedi et al. (2020) Iran  [136] | **Design:** RCT  **Methods:** Socio-demographic and outcome data collected at baseline and post 6 months intervention period  **Setting:** well-child clinic in Urmia  **Population:** Mother-Children pair (child < 3 years of age  **Size:** Total(pre/post)=110/100 Intervention=55/50, Control=55/55 | **Intervention:** Routine care + app with educational programme + weekly reminders to use the app  **Control:** Routine care, including monthly check-ups of child development | Unidirectional communication (educational information and reminders) | 1 | Educational information (age-related nutritional principles, behavioural methods for child feeding, child weaning time, introduction of complementary feeding and mothers´ health) | **^ Change in wasting (WHZ):** Intervention group 0.65±0.16, Control group 0.31±0.21, P=0.011.  **Change in maternal nutritional literacy (DID):**  Critical knowledge: Intervention group 5.56±0.78, Control group -0.94±0.59, P<0.001. Feeding attitudes: Intervention group 7.52±1.36, Control group -1.86±1.02, P<0.001. Nutrition practice: Intervention group 3.16±1.02, Control group -1.04±0.75, P=0.001. Overall: Intervention group 16.24±1.79, Control group -1.76±1.58, P<0.001  **Change in stunting (HAZ):** Intervention group 0.26±0.15, Control group -0.09±0.14, P=0.002  **Change in underweight (WAZ):** Intervention group 0.50±0.15, Control group 0.15±0.13, P=0.001 |
| 105.  Seyyedi et al. (2021) Iran  [137] | **Design:** RCT  **Methods:** Baseline questionnaire to collected demographic details and self-efficacy assessment (BSES-SF and KAP). Outcome data and participant characteristics collected during follow-up interviews  **Setting:** Well-child clinic in Urmia  **Population:** Mother-infant pairs, mother intended to breastfeed, infant less than 3 months old  **Size:** Total=80  Intervention=40, Control=40 | **Intervention:** Routine care + app with educational programme  **Control:** Routine care | Unidirectional communication (educational information)  Bidirectional communication (phone calls) | 1 | Educational information (importance of BF, behavioural methods for mothers, complementary feeding and EBF, pumping and manual expression, managing common breast-related and BF problems, and BF tips in special situations | **^ KAP score -knowledge (DID):** Intervention group 7.07 (5.37 to 8.77), Control group 1.40 (0.64 to 2.16) P<0.001  **^ KAP score – Attitudes (DID):** Intervention group 14.80 (11.42 to 18.48), Control group 6.05 (4.04 to 8.06) P<0.001  **^ KAP score – Practice (DID):** Intervention group 1.70 (0.85 to 2.55), Control group 0.90 (0.54 to 1.26) P=0.063  **^ KAP score – overall (DID):** Intervention group 23.57 (18.48 to 28.66), Control group 8.35 (5.6 to 11.1) P<0.001  **^ Self-efficacy (DID):** Intervention group 26.85 (19.72 to 33.98), Control group 0.40 (-4.77 to 5.57), P<0.001 |
| 106.  Shaaban et al. (2020) Egypt  [138] | **Design:** RCT  **Methods:** Baseline and follow-up interviews at 6 weeks and 6 months postpartum  **Setting:** Postpartum ward and family planning clinic of a tertiary care university hospital in Upper Egypt.  **Population:** Women who delivered in the hospital  **Size:** Total(pre/post)=1000/864  Intervention=500/432, Control=500/432 | **Intervention:** as control + phone call reminders 1 week before 6 weeks appointment, 2 days before appointment, and 2 follow-up after appointment (6 months)  **Control:** Postpartum contraception counselling + informational booklet + referral card to outpatient family planning clinic | Bidirectional communication (phone call reminders) | 1 | Reminders of future follow-ups and methods of contraception | **^ Initiation of Long-acting reversible contraception (LARC) method within the first 6 months postpartum:** Intervention group 30.2%, Control group 8.4%, P<0.001  **Initiation of Long-acting reversible contraception (LARC) method within the first 6 weeks postpartum:** Intervention group 26.6%, Control group 4.2%, P<0.001 |
| 107.  Shiferaw et al. (2016) Ethiopia  [139] | **Design:** Quasi-experimental study  **Methods:** Socio-demographic data collected at baseline. Cross-sectional surveys conducted at baseline and post-intervention. Outcome data was collected from obstetric records.  **Setting:** 10 Health facilities (5 intervention, 5 control) serving around 250.000 people in Semen Shewa Zone, Amhara region, central Ethiopia. Mixed rural and urban settings.  **Population:** Women visiting for ANC  **Size:** Total(pre)=933  Intervention=477, Control=456. Total(post)=1037  Intervention=514, Control=523.  Total HW(Health worker)=15 | **Intervention:** Health workers received a phone with an application to register new clients coming for ANC, delivery care or PNC. Reminders were sent automatically to health worker 7 days and 3 days before scheduled visits during ANC, delivery, and PNC. Health care workers contacted client to inform about scheduled visit. Health workers also received educational messages on danger signs and common complaints during pregnancy. **Control: -** | Unidirectional communication (text messages)  Bidirectional communication (Phone call to client) | 4 | Data collection on site | **^ At least 4 ANC visits:** Intervention group 27.0%, Control 23.4%, adj. OR 1.31 (95% CI 1.00 to1.72). Not statistically significant. Women in Urban areas were significantly more likely to have at least 4 ANC visits compared to women living in rural areas.  **^ Institutional delivery:** Intervention group 43.1%, Control group 28.4%, adj. OR 1.98 (95% CI 1.53 to 2.55).  **^ PNC in health centres:** Intervention group 41.2%, Control group 21.1%, adj. OR 2.77 (95% CI 2.12 to 3.61)  Women who visited the health centre were significantly more likely to delivery there and come back for PNC. |
|  |  |  |  | 5 | Longitudinal data collection and follow-up pregnant women |  |
|  |  |  |  | 8 | Reminders send to health worker to schedule follow-up |  |
|  |  |  |  | 9 | Educational information sent to health workers |  |
| 108.  Short et al. (2020) India  [140] | **Design:** Quasi-experimental study  **Methods:** Socio-demographic data collected at enrolment. Outcome data collected during home visits and  **Setting:** 6 PHC sites located in rural communities of the Belagavi district of Karnataka  **Population:** Pregnant women between 18-32 weeks of gestation intending to initiate BF and remain in the area until 6 months postpartum  **Size:** Total=222  Intervention=110, Control=112  Total(ASHAs)=25 | **Intervention:** Routine care + Breastfeeding education and support during home visits (ANC and PNC) by ASHA workers (28-32w of GA; 32-36w of GA; 1-3, 7, and 15 days postpartum; 1, 2, 4, and 6 months postpartum)  **Control:** Routine care | Unidirectional (multimedia content delivered via tablet) | 1 | Educational of and support to breastfeeding women to increase their commitment to 6 months EBF | **^ EBF until 6 months of life:** Intervention group 64%, Control group 34%, Adj. OR 3.57 (95% CI 1.80 to 7.07) P<0.0001  **^ Early initiation of BF:** Intervention group 82%, Control group 56%, Adj. OR 4.82 (95% CI 2.13 to 10.90) P<0.0001  **^ Colostrum given**: Intervention group 100%, Control group 94%, P=0.014  **^ No pre-lacteal feeding:** Intervention group 97%, Control group 84%, Adj. OR 10.2 (95% CI 2.54 to 40.71) P=0.0009  **^ No top-feeding**: Intervention group 92%, Control group 75%, Adj. OR 4.53 (95% CI 1.80 to 11.37)  **^ No supplementary feeding**: Intervention group 74%, Control group 54%, Adj. OR 2.24 (95% CI 1.12 to 4.47) P=0.001  **Total duration of EBF at month 1:** Intervention group 3.3%, Control group 4.5%. **at month 2:** Intervention group 4.4%, Control group 9.0%. **at month 3:** Intervention group 4.4%, Control group 6.7%. **at month 4:** Intervention group 6.6%, Control group 20.2%. **at month 5:** Intervention group 17.6%, Control group 23.6%. **at month 6:** Intervention group 63.7%, Control group 36.0% |
|  |  |  |  | 9 | 3-day BF counselling training course of ASHAs |  |
| 109.  Simonyan et al. (2013) Mali  [141] | **Design:** Quasi experimental study  **Methods:** Eight structured questionnaire completed every two weeks for each child  **Setting:** General population from three neighbouring districts  **Population:** 0-72 months old children with no diagnosed chronic disease who were enrolled in a telehealth programme  **Size:** Total(pre/post)=188/180  Intervention=99/91, Control=89/89 | **Intervention:** Routine care + weekly home (twice weekly for <1 years old) visits by WA for 16 weeks to assess general health of child (Anthropometry, feeding, sickness, symptoms, temperature, etc.)  **Control:** Routine care | Bidirectional communication (data collection + feedback from GP in case of an emergency) | 4 | Data collection on site | **^ Healthcare utilisation**: Intervention group 93.4%, Control group 31.5%, OR 2.2 (95% CI 1.26 to 3.85).  **Total number of disease episodes:** Intervention group 236, Control group 168. Episodes for specific disease are given in the paper. These were not statistically significantly different from the two groups. |
|  |  |  |  | 5 | Health data transferred to database |  |
| 110.  Singh et al. (2020) Nepal  [142] | **Design:** Cluster RCT  **Methods:** Socio-demographic data collected via interviews. Outcome data extracted from medical records.  **Setting:** 52 health posts from Two primary health care facilities catchment area in Dhanusha district in Southern Terai region, Nepal  **Population:** Pregnant women, 13-28 weeks of GA  **Size:** Total(pre/post)=426/413  Intervention=219/214, Control=207/199 | **Intervention:** Female community health volunteers (FCHV) capacity building by regular supervision and monitoring + text messages sent to pregnant women once every 2 weeks (4-6months of GA) and once weekly until delivery  **Control:** - | Unidirectional communication (text messages) | 1 | Educational information (MCH service utilisation and dietary intake during pregnancy ad postpartum) | **^ Weight at 13-28 weeks of GA (mean±SD):** Intervention group 45.93±5.90, Control group 45.66±5.52, P=0.624  **^ Haemoglobin at 13-28 weeks of GA (mean±SD):** Intervention group 11.20±1.08, Control group 11.0±1.15, P=0.214  **^ Weight at 38-39 weeks of GA (mean±SD):** Intervention group 52.36±6.28, Control group 50.94±5.90, P=0.018  **^ Haemoglobin at 38-39 weeks of GA (mean±SD):** Intervention group 11.43±1.00, Control group 11.11±1.10, P=0.024  **^ GA weight gain:** Intervention group 6.9 (95% CI 6.2 to 7.7), Control group 5.8 (5.2 to 5.9) P<0.0001  **^ Haemoglobin change:** Intervention group .21 (95% CI 0.13 to 0.22), Control group 0.10 (0.07 to 0.13) P<0.0001 |
| 111.  Smith et al. (2015) Cambodia  [143] | **Design:** RCT (MOTIF)  **Methods:** Baseline interview data collection  **Setting:** Two Peri-urban Marie Stopes clinics near Phnom Penh (Chbar Ambov and Takmao) and two rural (Battambang and Siem Reap)  **Population:** Women seeking an induced abortion  **Size:** Total(pre/4m/12m)=500/431/328  Intervention=249/211/169, Control=251/220/159 | **Intervention:** As control + 6 voice messages over a 3 months period + access to phone support from counsellor as needed + opt for text reminders according to method of contraception chosen  **Control:** Post-abortion FP counselling + follow-up appointment + access to hotline. | Unidirectional communication (voice messages)  Bidirectional communication (phone counselling) | 1 | Educational information and guidance | **Self-reported use of an effective contraception method 4 months after abortion:** Intervention group 64%, Control group 46%, RR 1.39 (95% CI 1.17 to 1.66)  **Self-reported use of an effective contraception method 12 months after abortion:** Intervention group 50%, Control group 43%, RR 1.16 (95% CI 0.92 to 1.47)  *List of secondary outcomes related to contraception methods, pregnancy, abortion, domestic abuse, etc.* |
| 112.  Souza et al. (2021) Brazil  [144] | **Design:** RCT  **Methods:** Socio-demographic data collected during structured interviews, follow-up data collected from medical records and Healthy Gestation app.  **Setting:** 2 Family Heath Units located in two municipalities in Northern Brazil  **Population:** Pregnant women  **Size:** Total(pre/post)=88/75  Intervention=44/36, Control=44/39 | **Intervention:** Routine prenatal care + healthy gestation app + monthly meetings  **Control:** Routine prenatal care + group meetings and prenatal consultations of usual risk | Unidirectional communication (app)  Bidirectional communication (app – contact function) | 1 | Educational information (prenatal care, childbirth, the puerperium, BF, the virtual pregnant woman’s booklets, alarm clock as reminders for prenatal consultations) | **Prenatal adherence:**  =<5 consultations: Intervention group 0%, Control group 51.3%  6/7 consultations: Intervention group 16.7%, Control group 41.0%  >=8 consultations: Intervention group 83.3%, Control group 7.7%  P=0.018 |
| 113.  Sun et al. (2021) China  [145] | **Design:** RCT  **Methods:** Edinburgh Postnatal depression score (EPDS) or Patient Health questionnaire-9 (PHQ9), socio-demographic, pregnant-related, and mental health indicators collected at baseline. Follow-up data collected via self-administered surveys at 5 time-points.  **Setting:** Obstetrics clinic of a tertiary hospital in Jinan, Shandong, East of China  **Population:** Pregnant women who scored above the threshold or positive depression symptoms, 12 to 20 weeks of GA  **Size:** Total(pre/post)=168/92  Intervention=84/52, Control=84/40 | **Intervention**: 8 weeks smartphone-based mindfulness training including mindfulness journal, body scan, mindful breathing, mindful stretching, and mindful medication, mindful eating, and mindful walking  **Control:** 8 weeks regular WeChat health consultations and weekly contact with trained nursing assistant | Unidirectional communication (training program)  Bidirectional communication (WeChat) | 1 | Educational information via text, audio, and visual material (understand mindfulness, be in the present, negative emotions, accept difficulties, thoughts are just thoughts, enjoy daily happiness, mindful pregnancy and childbirth continued mindfulness practice) | **EPDS (ITT mean±SD):**  **T1)** Intervention group 7.99±3.873, Control group 8.55±4.593.  **T2 - 4 weeks after enrolment, intervention midway)** Intervention group 6.78±4.816, Control group 7.63±5.217, P=0.11  **T3 – 8 weeks after enrolment, intervention endpoint)** Intervention group 6.49±4.497, Control group 9.09±6.241, P=0.03  **T4 – before childbirth)** Intervention group 6.14±4.552, Control group 8.02±6.002. P=0.01  **T5 – 6 weeks postpartum)** Intervention group 6.77±4.693, Control group 6.25±5.098, P=0.12 |
| 114.  Tahir and Al-Sadat (2013) Malaysia  [146] | **Design:** RCT  **Methods:** Self-administered questionnaire (first day postpartum) and telephone interview (1, 4, and 6 months postpartum)  **Setting:** Public maternity hospital in urban settings  **Population:** Pregnant women <= 18 years of age, singleton pregnant, 37+ weeks of GA at delivery  **Size:** Total(pre/post)=357/318  Intervention=179/160, Control=178/158 | **Intervention:** Routine postnatal BF support + lactation counselling via telephone twice monthly by certified lactation counsellors until breastfeeding came to full stop  **Control:** Routine postnatal BF support | Bidirectional communication (Phone counselling) | 1 | Postnatal support and counselling | **^ Exclusive breastfeeding at 1 month postpartum:** Intervention group 84.3%, Control group 74.7%, Unadj. OR 1.83 (95% CI 1.05 to 3.16) P=0.042, adj. OR 1.63 (95% CI 0.82 to 3.22).  **^ Exclusive breastfeeding at 4 months postpartum:** Intervention group 42.0%, Control group 39.0%, Unadj. OR 1.13 (95% CI 0.83 to 1.40) P=0.67, adj. OR 1.173 (95% CI 0.71 to 1.94).  **^ Exclusive breastfeeding at 6 months postpartum:** Intervention group 12.5%, Control group 12.0%, Unadj. OR 1.05 (95% CI 0.54 to 0.04) P=0.90. |
| 115.  Talebi et al. (2022) Iran  [147] | **Design:** RCT  **Methods:** Pregnancy Physical Activity Questionnaire were used to collect outcome data. Socio-demographic data collected at baseline.  **Setting:** Urmia health centers  **Population:** Pregnant healthy women  **Size:** Total(pre(post)=90/71  Intervention=45/35, Control=45/36 | **Intervention:** As control + receiving educational content as written, audio or video to improve physical activity during 16 sessions in 8 weeks, delivered twice weekly. Reminder messages sent twice weekly to be physically active and adhere to a good diet.  **Control:** Quiet match receiving routine pregnancy care + joined a virtual group on WhatsApp + individual diet and material about weight gain during pregnancy | Bidirectional communication (Virtual chat) | 1 | Educational information regarding diet and physical activity | **^ Change in mean daily total physical activity level (MET)**  Intervention group pre 830.25±630.99, post 175±61.54, P<0.001  Control group pre 654.80±564.97, post 75.75±34.41, P<0.001  Intervention group pre 830.25±630.99, Control group pre 654.80±564.97, P=0.22  Intervention group post 175±61.54, Control group post 75.75±34.41, P<0.001  **Weight during pregnancy**  Intervention group pre 72.41±13.25, post 74.57±13.43, P<0.001  Control group pre 72.17±13.63, Post 79.00±15.31, P<0.001  Intervention group pre 72.41±13.25, Control group pre 72.17±13.63, P=0.94  Intervention group post 74.57±13.43, Control group post 79.00±15.31, P=0.20 |
| 116-117.  Tian et al. (2021) and Huang et al. (2021) China  [76, 148] | **Design:**  RCT  **Methods:** Socio-demographic questionnaire completed at baseline. Outcome data recorded in lifestyle diaries  **Setting:** 8 Prenatal care institutions  **Population:** Pregnant women <31 week of GA diagnosed with GDM (OGTT)  **Size:** Total(pre/post)=309/269  Intervention=147/133, Control=162/136 | **Intervention:** Routine prenatal care + WeChat group management on a weekly basis + individualised guidance to self-management + lessons and articles + WeChat to have questions answered + lifestyle diaries to record blood sugars, diet, PA, etc.  **Control**: Routine prenatal care (every 2 week after GDM diagnosis) + GDM management lesson + lifestyle diaries to record blood sugars, diet, PA, etc | Bidirectional communication (WeChat) | 1 | Educational informational and guidance to self-manage GDM | **^ Glycaemic qualification rate:**  At T1 (when receiving health management) rates were similar between intervention and control group. From T2 onward the rates were higher in the intervention group compared to the control group at nearly all time points.  **Postpartum diabetes:**  **Fasting OGTT mmol/L(mean±SD):** Intervention group 4.86±0.64, Control group 4.79±0.45, P=0.8000  **1-h** **OGTT mmol/L(mean±SD):** Intervention group 9.18±2.13, Control group 8.64±1.36, P=0.1975  **1-h** **OGTT mmol/L(mean±SD):** Intervention group 6.90±1.77, Control group 6.64±1.17, P=0.7891  **T2DM:** Intervention group 12.36%, Control group 3.88, P=0.0291 |
| 118.  Uddin et al. (2016) Bangladesh  [149] | **Design:** Quasi-experimental study  **Methods:** EPI cards when available, maternal recall with structured questionnaires when cards not available.  **Setting:** Two rural hard-to-reach sub-districts (one intervention, one control) and two Dhaka city zones with most street dwellers (one intervention, one control). Intervention and control were matched on four parameters  **Population:** Pregnant women and mothers with children age 0-11 months above the age of 18.  **Size:** Total rural(pre/post)=1040/1040 Intervention=520, Control=520  Total Urban(pre/post)=1038/1042 Intervention=518/520, Control=520/522 | **Intervention:** Mothers were sent automatic SMS reminders one day before upcoming EPI sessions as well as at opening time at the day of EPI session and two hours before closing time.  **Control: -** | Unidirectional communication (text messages) | 1 | Vaccination appointment reminders | **^ Full vaccination:** Intervention group(rural area) pre 58.9%, post 76.8%, Control group(rural area) pre 65.9%, post 55.2%, Diff-in-Diff OR 3.6 (95% CI 1.5 to 8.9) P<0.001.  Intervention group(urban area) pre 40.7%, post 57.1%. Control group(urban area) pre 44.5%, post 33.9%, Diff-in-Diff OR 2.3 (95% CI 1.1 to 5.5) P<0.05.  Intervention effect on age-appropriate vaccination was positive for all age groups, with Diff-in-Diffs ranging from +13.1% to 29.5% and ORs ranging from 2.5 to 4.6 (p<0.001 across all rural vs. urban comparisons per age groups).  The largest intervention effect was on age-appropriate vaccination for children over 70 days, OR 4.6 in urban intervention area (95% CI2.1 to 7.8) P<0.001  *Attributable to the intervention (Difference-in-difference parameters = DID)* |
|  |  |  |  | 3 | Birth registration: Mothers sent SMS birth notification(home birth), or at delivery(health facility) or at EPI centers. |  |
| 119.  Ugwa et al. (2020) Nigeria [150] | **Design:** Cluster RCT  **Methods:** Pre-training assessment to assess baseline knowledge and skills among health workers, which was assessed again after training, after 3 and 12 months.  **Setting:** 30 Health facilities in Ebonyi state (South-East zone) and 30 health facilities in Kogi State (North-Central zone)  **Population:**  **Size:** Total(pre/post)=323/299  Intervention=184/172, Control=139/127 | **Intervention:** LDHF/m-mentoring on BEmONC (7 functions) using in-service learning with 4 days + 4 days training session 1 month apart + mobile phone mentorship to resolve emergency issues at the workplace + weekly text reminders and questions to HCW on topics reviewed + monthly support calls from master mentor  **Control:** 8 straight days of lectures with practice session on simulators, outside the participants workplace (normal delivery, AMTSL, neonatal resuscitation, case management of PEE and PPH) | Unidirectional communication (text messages reminders to trainees to reinforce gains + text messages reminders and questions)  Bidirectional communication (calls to resolve emergency issues + mentoring calls from trainer to provide support) | 9 | Training of health workers | **^ Increase in knowledge, clinical skills and retention of clinical competency at 3 and 12 months post-training (infection prevention, normal birth, AMTSL, management of eclampsia, essential newborn care, neonatal resuscitation):**  Baseline: Control group had better knowledge test score for neonatal resuscitation (P<0.001)  Immediately after training: Control group had better knowledge test score for neonatal resuscitation (P<0.001), AMTSL (P<0.002), and Essential newborn care (P<0.001).  3 months post training: No difference  12months post training: No difference  Health workers in both arms showed improvement in overall pass rates in clinical skills competency improving from around 30% at baseline to 75% and above at endline. |
| 120.  Unger et al. (2018) Kenya [151] | **Design:** RCT  **Methods:** Questionnaires administered at baseline and recurring study visits (approximately 2 weeks, 10 weeks, and 24 weeks postpartum)  **Setting:** One government health center MCH clinic in Nairobi county  **Population:** Pregnant women seeking ANC, 14 years of age or older, less than 36 weeks of gestation  **Size:** Total(pre)=298,  Intervention1=99, Intervention2=99, Control=100  Total(24 weeks post-partum)=259,  Intervention1=86, Intervention2=82, Control=91 | **Intervention arm 1:** Routine care + weekly educational or motivational text message from enrolment until 12 weeks postpartum  **Intervention arm 2:** Routine care + weekly educational or motivational text message including a question related to content. Women were free to respond and/or send messages regarding concerns and questions from enrolment until 12 weeks postpartum  **Control:** Routine care | Unidirectional communication for Intervention arm 1 (text messages)  Two- way communication for Intervention arm 2 (chat) | 1 | Educational and motivational information | **^ Facility delivery**: Overall 98% facility delivery. Intervention arm1 vs control, RR 1.00 (95% CI 0.97 to 1.03) P=0.99. Intervention arm2 vs control, RR 0.99 (95% CI 0.95 to 1.03) P=0.54.  **^ Probability of exclusive breastfeeding (10 weeks):** Intervention arm1 0.93 (95% CI 0.86 to 0.97), Control 0.79 (95% CI 0.69 to 0.86) P=0.003. Intervention arm2 0.96 (95% CI 0.89 to 0.98), Control 0.79 (95% CI 0.69 to 0.86) P=0.004.  **^ Exclusive breastfeeding (16 weeks):** Intervention arm1 0.82 (95% CI 0.72 to 0.89), Control 0.62 (95% CI 0.52 to 0.71) P=0.002. Intervention arm2 0.93 (95% CI 0.85 to 0.97), Control 0.62 (95% CI 0.52 to 0.71) P<0.0001.  **^ Exclusive breastfeeding (24 weeks):** Intervention arm1 0.49 (95% CI 0.38 to 0.59), Control 0.41 (95% CI 0.31 to 0.51) P=0.3. Intervention arm2 0.62 (95% CI 0.51 to 0.72), Control 0.41 (95% CI 0.31 to 0.51) P=0.005**.**  **^ Probability of contraceptive use (10 weeks):** Intervention arm1 0.42 (95% CI 0.32 to 0.53), Control 0.33 (95% CI 0.24 to 0.44) P=0.24. Intervention arm2 0.31 (95% CI 0.23 to 0.42), Control 0.33 (95% CI 0.24 to 0.44) P=0.74.  **^ Probability of contraceptive use (16 weeks):** Intervention arm1 0.72 (95% CI 0.62 to 0.81), Control 0.57 (95% CI 0.47 to 0.67) P=0.03. Intervention arm2 0.73 (95% CI 0.63 to 0.82), Control 0.57 (95% CI 0.47 to 0.67) P=0.02.  **^ Probability of contraceptive use (24 weeks):** Intervention arm1 0.83 (95% CI 0.74 to 0.90), Control 0.77 (95% CI 0.69 to 0.85) P=0.39. Intervention arm2 0.83 (95% CI 0.75 to 0.90), Control 0.77 (95% CI 0.69 to 0.85) P=0.33. |
| 121.  Vanhuyse et al. (2022) Kenya [152] | **Design:** Cluster RCT  **Methods:** Data collected from an electronic card reading system + pre and post surveys  **Setting:** 48 health facilities located in Siaya county, Kenya  **Population:** Pregnant women recruited during first ANC visit  **Size:** Total(pre)=5471  Intervention=2522, Control=2949  Total(post all/any primary outcomes)= 2262/5388  Intervention=1235/2490, Control=1027/2898 | **Intervention:** Routine care + Conditional cash transfer (US$4.5) were made to pregnant women who attended they ANC appointments, delivery, PNC visits, and childhood immunisation  **Control:** Routine care | Unidirectional communication | 12 | Conditional cash transfer | **^ ANC attendance:** Intervention group 67%, Control group 60%, Adj. OR 1.90 (95% CI 1.36 to 2.66) P<0.001  **^ Delivery in a health facility:** Intervention group 90%, Control group 92%, Adj. OR 0.58 (95% CI 0.25 to 1.35) P=0.20  **^ Attendance for at least one PNC between 4 and 12 months:** Intervention group 82%, Control group 81%, Adj. OR 1.25 (95% CI 0.74 to 2.10) P=0.40  **^ Child immunisation attendance:** Intervention group 88%, Control group 85%, Adj. OR 1.74 (95% CI 1.10 to 2.77) P=0.02 |
| 122.  Von Dadelszen et al. (2020) Mozambique, Pakistan, India [153] | **Design:** Cluster RCT (CLIP intervention)  **Methods:** data collection surveys carried out quarterly  **Setting:** 4 Primary health centers in Maputo province and 8 from Gaza province, Southern Mozambique  **Population:** Married pregnant women  **Size:**  Total pregnancies(pre/post) =69330/61988,  Intervention=36088/32290, Control=33322/29698 | **Intervention:** Community engagement + CHW-led CLIP antenatal visits guided by CLIP POM mHealth app to address the delays in triage, transport, and treatment    **Control:** Routine ANC | Unidirectional communication (data collection) | 3 | Maternal and Newborn Health Registry | **^ All-cause maternal mortality:** Intervention group 0.2%, Control group 0.2%, Adj. OR 1.05 (95% CI 0.67 to 1.64) P=0.84  **^ All-cause maternal morbidity:** Intervention group 10.3%, Control group 9.2%, Adj. OR 1.20 (95% CI 0.83 to 1.74) P=0.32  **^ All-cause perinatal mortality, late neonatal mortality, neonatal morbidity:** Intervention group 17.4%, Control group 16.0%, Adj. OR 1.10 (95% CI 0.89 to 1.37) P=0.38  **Birth preparedness and complication readiness:** Intervention group 53.4%, Control group 45.5%, Adj. OR 0.91 (95% CI 0.41 to 2.02) P=0.82  **Delivery in facility with emergency care:** Intervention group 49.3%, Control group 48.5%, Adj. OR 0.84 (95% CI 0.59 to 1.19) P=0.32  **Proportion of facility births:** Intervention group 85.5%, Control group 78.7%, Adj. OR 1.06 (95% CI 0.82 to 1.36) P=0.66 |
|  |  |  |  | 4 | Blood pressure measurements and test for proteinuria |  |
|  |  |  |  | 5 | Data transfer from POM mobiles to central REDCap server |  |
|  |  |  |  | 6 | Electronic decision support with pictograms and visual prompts directing CHWs to recommend appropriate care |  |
| 123.  Watterson et al. (2020) Samoa [22] | **Design:** Quasi-experimental study  **Methods:** Demographic data and outcome data was collected from medical records and ANC registration books at ANC clinics  **Setting:** Island of Upolu  **Population:** Pregnant women visiting coming for ANC  **Size:** Total(pre/post)=979/803  Intervention=728/552, Control 251 | **Intervention:** Routine care + 2 educational messages weekly adapted to AG + reminders 1 day prior to scheduled appointment and a final reminder 4 weeks after overdue appointment  **Control:** Routine care | Unidirectional communication (text messages) | 1 | Antenatal care appointment reminders and educational information | **^ ANC visits attended (ITT mean±SD)**: Intervention group 2.2 ± 1.9, Control group 2.6 ± 1.7, P=0.01 |
| 124.  Wu et al. (2020) China  [154] | **Design:** RCT  **Methods:** Face-to-face data collection at baseline and follow-up via phone interviews.  **Setting:** 13 townships in Huzhu County, Qinghai Province, Northwest China  **Population:** Pregnant women 11 to 37 week of GA  **Size:** Total(pre/post)=344/319  Intervention=170/108, Control=174/109 | **Intervention:** As control + access to “Ke Xue Wei Yang”, a module in optimal feeding + additional messages delivered during late pregnancy, first month postpartum, and 4 months postpartum (3 times weekly)  **Control:** Routine care + follow WeChat called Huzhu County Centre | Unidirectional communication (text messages) | 1 | Educational information (BF and complementary feeding messages, feeding knowledge competition, a baby growth chart, and an online forum) | **^ EBF at 0-1 month:** Intervention group 81.1%, Control group 63.3%, Adj. OR 2.75 (95% CI 1.58 to 4.78) P<0.001  **^ PBF at 0-1 month:** Intervention group 83.8%, Control group 67.6%, Adj. OR 2.77 (95% CI 1.55 to 4.96) P<0.001  **^ EBF at 2-3 month:** Intervention group 73%, Control group 63.2%, Adj. OR 1.53 (95% CI 0.94 to 2.49) P=0.09  **^ PBF at 2-3 month:** Intervention group 74.3%, Control group 65.1%, Adj. OR 1.60 (95% CI 0.96 to 2.64) P=0.07  **^ EBF at 4-5 month:** Intervention group 46.3%, Control group 42.2%, Adj. OR 1.37 (95% CI 0.78 to 2.39) P=0.27  **^ PBF at 4-5 month:** Intervention group 53.7%, Control group 48.6%, Adj. OR 1.46 (95% CI 0.82 to 2.51) P=0.20  **Proportion of early initiation:** Intervention group 62.8%, Control group 72.7%, Adj. OR 0.66 (95% CI 0.40 to 1.09) P=0.11  **Pre-lacteal feeding rate:** Intervention group 39.9%, Control group 45.3%, Adj. OR 0.77 (95% CI 0.48 to 1.24) P=0.28  **Knowledge of BF practices was assessed at baseline and all follow ups. Knowledge significantly increased in both groups compared to baseline.** |
| 125.  Xie et al. (2018) China  [155] | **Design:** Cluster RCT  **Methods:** Socio-demographic and pregnancy-related data collected at the time of pregnancy confirmation and 42 days postpartum.  **Setting:** Northwestern region of Hunan,  **Population:** Pregnant women  **Size:** Total(pre)=25,236/13,937,  Intervention=13,332/6771, Control=11,904/6966 | **Intervention:** Text messages sent to village health workers and pregnant women according to periods (1^st^ trimester, 2^nd^ trimester, 3^rd^ trimester, and postpartum)  **Control:** | Unidirectional communication (text messages) | 1 | Educational information | **Early pregnancy visit:** Intervention group 93.16%, Control group 95.38%, Adj. OR 0.99 (95% CI 0.99 to 1.00)  **Prenatal screening:** Intervention group 48.6%, Control group 34.18%, Adj. OR 0.25 (95% CI 1.21 to 1.31)  **Folic acid supplementation:** Intervention group 69.9%, Control group 77.96%, Adj. OR 0.92 (95% CI 0.90 to 0.93)  **Caesarean delivery:** Intervention group 36.75%, Control group 42.02%, Adj. OR 0.95 (95% CI 0.91 to 0.98)  **Maternal death, Perinatal death, Obstetric haemorrhage, Birth weight < 2.5kg, Birth weight > 4.0kg, Thyroid test, Hearing test Phenylketonuria test, Syphilis test,** **Hepatitis B test** |
| 126.  Xuto et al. (2022) Thailand  [156] | **Design:** RCT  **Methods:** Demographic characteristics Questionnaire, Pregnancy Outcomes Record, Health Care Behaviour during pregnancy questionnaire, State-Trait Anxiety Inventory  **Setting:** Maharaj Nakorn government hospital in Chiang Mai and Sunpasitthiprasong government hospital, Ubon Ratchathani province  **Population:** Pregnant women 12+1 week of GA  **Size:** Total(pre/post)=66/59  Intervention=33/30, Control=33/29 | **Intervention:** Routine care + Received automatic 2 weekly text messages for 28 weeks  **Control:** Routine care | Unidirectional communication (text messages) | 1 | Educational information (Nutrition, coping with discomfort during pregnancy, warning signs of pregnancy complications, mental health care, preparation for experiencing labour, and signs and symptoms that lead to the delivery stage) | **Gestational weight gain -kg (mean±SD):** Intervention group 12.77±4.94, Control group 11.98±6.42, P=0.60  **Haemoglobin -g/dl (mean±SD):** Intervention group 12.44±1.25, Control group 11.75±1.37, P=0.33  **Glucose challenge test -mg/dl (mean±SD):** Intervention group 130.27±28.31, Control group 141.90±13.11, P=0.12  **Baby weight -kg (mean±SD):** Intervention group 2.93±3.65, Control group 2.96±4.46, P=0.73  **State-anxiety (mean±SD):** Intervention group 35.23±8.50, Control group 40.79±9.28, P=0.02 |
| 127.  Zhang et al. (2019) China  [157] | **Design:** RCT  **Methods:** Demographic and clinical data collected at baseline. Outcome data collected via 24h diet records and nutrition assessment at 3 visits, blood tests and other clinical outcomes  **Setting:** Kunshan Maternity and Child Care Center and the International Peace Maternity and Child Health Hospital of China Welfare Institute  **Population:** Overweight or obese pregnant women with ANC =<16 weeks of GA  **Size:** Total(pre/post)=400/369  Intervention=200/186, Control=200/183 | **Intervention:** As control + additional diet Glycaemic Index (GI) and glycaemic load (GL) calculations on the diet assessment and a diet plan made to achieve low GI goal using mobile app (DietGI)  **Control:** Standard nutrition and physical activity consultation, advice on gestational weight gain, individualised diet plan. | Unidirectional communication    Bidirectional communication | 1 | Educational information | **^ GDM:** Intervention group 22.5%, Control group 21.5%, P=0.33  **^ Maternal insulin levels before delivery (median (IQR)):** Intervention group 12.8 (9.3-13.2), Control group 12.8 (10.5-12.8), P=0.18  **^ Cord blood C-peptide levels -ng/mL (mean±SD):** Intervention group 0.86±0.67, Control group 0.85±0.61, P=0.84  **Gestational weight gain -kg (mean±SD):** Intervention group 9.6±7.4, Control group 11.2±6.3, P=0.02  **Gestational hypertension:** Intervention group 22.8%, Control group 16.2%, P=0.11  **Caesarean section:** Intervention group 124±67.0, Control group 107±58.5, P=0.09  **Birth weight -g (mean±SD):** Intervention group 3513.86±522.4, Control group 3452.5±527.1, P=0.26.  **Preterm birth:** Intervention group 8.7%, Control group 11.7%, P=0.11. **Macrosomia:** Intervention group 23%, Control group 19%, P=0.15 |
| 128.  Zhou et al. (2016) China  [158] | **Design:** Cluster RCT  **Methods:** Baseline and follow-up survey. Finger blood analysis of haemoglobin concentration (anaemia)  **Setting:** 351 rural villages as clusters from 11 poverty counties in Southern Shaanxi  **Population:** infants aged 6-12 months  **Size:** Total(pre/post)=1818/1393  Free delivery group(FDG)=600/438,  Text message group(TMG)=599/448 Control=619/507 | **FDG:** One-on-one health education training on nutrition and feeding practices + 6 months free supply of micronutrient supplement packets for home fortification  **TMG:** FDG + daily text messages reminders for six months  **Control: -** | Unidirectional communication (text messages) | 1 | Reminders to use micronutrient powders on a daily basis | **^ Compliance (total number of opened/unopened packets):**  FDG 40.4%, TMG 44.9%, P=0.09  **Anaemia (baseline):** FDG 46.1%, TMG 49.8%, Control group 47.9%, P=0.63  **Anaemia (endline):** TMG reduced anaemia by 7 percentage point [marginal effect -0.07 (95% CI -0.12 to -0.01) P=0.02]. No difference between FDG and control [-0.03 (95% CI -0.09 to 0.03) P=0.38] |
| 129.  Zhou et al. (2020) China  [159] | **Design:** Quasi-experimental study  **Methods:** Sociodemographic, health, and pregnancy related items collected via baseline survey, Follow-up data collected at first postnatal visit at home  **Setting:** Gaoling Maternal and Child Health Center, Gaoling County, Shaanxi Province  **Population:** Pregnant women  **Size:** Total(pre/post)=4624/2115  Group1=1110/526, Group2=1173/518, Group3=1097/497, Group4=1244/572 | **Group1:** Basic text messages about pregnancy (Control) – Gestational stages, reminders for prenatal visits, promotion of SBA  **Group2:** Group1 + Care-Seeking text messages (CS) - government. subsidized programs, warning signs, importance of case-seeking  **Group3:** Group1 + Good Household Prenatal Practices (GHPP) – nutrition, exercise, depression awareness, BF  **Group4:** Group1, Group2, Group3 | Unidirectional communication (text messages) | 1 | Educational information and appointment reminders | **Inappropriateness of weight for gestational age (IWGA):**  **Short for GA (below 10^th^ percentile):** 243(11.6%) in total  **Macrosomia(>=4000):** 164 (7.8%) in total  IWGA in total=407(19.5%), group1=23%, Group2=19.6%, Group3=18.9%, Group4=16.5%  **IWGA (group1 vs group2):** Adj. OR 0.79 (95% CI 0.58 to 1.07),  **IWGA (group1 vs group3)**: Adj. OR 0.77 (95% CI 0.57 to 1.05),  **IWGA (group1 vs group4)**: Adj. OR 0.65 (95% CI 0.48 to 0.89) |
| 130.  Zhuo et al. (2022) China  [160] | **Design:** RCT  **Methods:** 5-item Medication Adherence Report Scale (MARS-5) at 1 and 4 weeks after inclusion  **Setting:** First Affiliated Hospital of Shantou University Medical College  **Population:** Pregnant women diagnosed with GDM who received initial insulin treatment within 24-35 weeks of GA  **Size:** Total(pre/post)=124/119  Intervention=62/58, Control=62/61 | **Intervention:** As control + diabetes self-management app (upload blood glucose values, insulin doses, diet, physical exercise) with educational information provided by pharmacists + ability to interact with pharmacists  **Control:** Routine GDM care during pregnancy (self-monitoring) | Bidirectional communication (data reporting + interaction with health professional) | 1 | Educational information (diabetes management) | **^ Medication adherence (score=25):** Intervention group 69%, Control group 34.4%, Adj. OR 4.256, (95% CI 1.864 to 9.719) P=0.001 |
| 131.  Zurovac et al. (2011) Kenya  [161] | **Design:** Cluster RCT  **Methods:** Quantitative health facility surveys conducted at baseline(T0), at 6 months(T1), and 6 months post intervention(T2). Qualitative interviews with health workers. Data collection regarding drug availability, malaria diagnostics, retrospective malaria morbidity  **Setting:** 107 rural government health facilities in 11 districts in two malaria endemic areas  **Population**: Children under 5 needing AL (artemether-lumefantrine) management.  **Size:** Total(T0/T1/T3)=861/749/659  Intervention=422/358/332, Control=439/391/327 | **Intervention:** All healthcare workers doing outpatient consultations received text messages about malaria case-management every week for 6 months (2 messages daily during weekdays).  **Control: -** | Unidirectional communication (reminders) | 6 | Educational information decision support | **^ Composite performance:**  Correctly managed at T0: Intervention group 20.5%, Control group 11.1%.  Correctly managed at T1: Intervention group 49.6%, Control group 16.5%.  Correctly managed at T2: Intervention group 51.4%, Control group 17.5%.  Percent change between T0 to T1 in the difference between intervention and control group = 23.7% (95% CI 7.6 to 40.0). Percent change between T0 to T2 in the difference between intervention and control group = 24.5% (95% CI 8.1 to 41.0).  *All children were between 21-26months* |
|  |  |  |  | 9 | Educational information on malaria case management (reminder/advise) |  |

^*^mHealth form: 1=unidirectional communication, 2=Bidirectional communication, 3=multi-directional communication

^**^mHealth Function is the categorisation of mHealth applications as described by Labrique et al. (See information box 1).

Abbreviations: AF, ASHA facilitator (monitoring, facilitating and supervising 10-20 ASHAs); AMTSL, active management of the third stage of labour; ANM, auxillary nurse midwife (qualified MNCH provider); ASHA, accredited social health activist (a female frontline worker native to village, employed by the Ministry of Health and Family Welfare, MoHFW); AWW, Anganwadi worker (employed by the Ministry of Women and Child Development, MWCD); BCG, Bacillus Calmette-Guérin; BEmONC, Basic Emergency Obstetric and Newborn Care; BF, Breastfeeding; BS, blood sugar; CCPF, Chipatala cha pa Foni; CHW, community health worker; CWS, Cambridge Worry Scale; DASS, Depression Anxiety Stress Scale; DPT, Diphtheria, Pertussis, Tetanus; EBF, exclusive breastfeeding; EID, early infant diagnosis; EmONC, Emergency Obstetric and Neonatal Care; EPDS, Edinburgh Postnatal Depression Scale; EPI, Expanded Programme on Immunisation (to protect against Polio, diphtheria, tuberculosis, pertussis, measles and tetanus. 1 dose of BCG, 3 doses of DTP, 3 doses of OPV, and 1 dose of Measles (MCV)); FBS, Fasting Blood Sugar; FFQ, Food Frequency Questionnaire; FGD, focus group discussion; FP, family planning; HAZ, Height-for-Age Z-score; HF, health facility; GA, gestational age; GDM, gestational diabetes mellitus; icddr,b, International Centre for Diarrhoeal Disease Research, Bangladesh; IDI, In-depth interview; IPAQ-SF, International Physical Activity Questionnaire; ITT, intention to treat; IYCF, infant and young child feeding; KEMRI/CDC, Kenya Medical Research Institute/Centers for Disease Control and Prevention’s Health; LGA, local government areas (whose administration manage primary healthcare facilities); MCV, measles-containing vaccines; MACCI, maternal care services (>=3 ANC home visits by ASHA, full ANC service, facility delivery) + neonatal care services (>=5 PNC home visits within first month postpartum) + young infant care services (DPT3, EBF) + care seeking during complications (Neonatal complications, Diarrhoea, Fever); MD, medical doctor (in overall charge of the PHC); MET, metabolic equivalent of task; MMAS-8 questionnaire, Morisky Medication Adherence Scale questionnaire; MUAC, mid-upper arm circumference; NVP, nevirapine; OGTT, oral glucose tolerance test; OPV, oral polio vaccine; PA, physical activity; PBF, predominant breastfeeding; PHC, primary healthcare center; PEE, pre-eclampsia and eclampsia; Pentavalent, (DPT, Hepatitis B and Hib (Haemophilus influenzae type b)); PP, per protocol; PPH, Postpartum haemorrhage; SF-12, short form heath survey; SSI, semi structured interview; STAI, Spielberger State-Trait Anxiety Inventory; T2DM, type 2 diabetes mellitus; LDHF/m-mentoring, low dose, high frequency plus mobile mentoring training; WAZ, Weight-for-Age Z-score; WHZ, Weight-for-Height Z-score;
