# Supplementary material for: Impact of mHealth interventions on maternal, newborn, and child health from conception to 24 months postpartum in low- and middle-income countries: a systematic review
Source: BMC Med. 2024 May 15;22:196. doi: 10.1186/s12916-024-03417-9 (PMC11095039; doi:10.1186/s12916-024-03417-9)
Supplement: Supplementary file 3 — Additional file 3: Table A3 Outcomes by health domains [file 12916_2024_3417_MOESM3_ESM.docx]

**Table A3** Outcomes under each health domain

|  | **Health Domain: Antenatal care (ANC)** | | |
| --- | --- | --- | --- |
| **Study** | **Number of ANC visits** | **Supplementation, injections, and test** | **ANC Others** |
| Abbaspoor et al. (2020) |  |  | ^ Physical activity level |
| Aksoy Derya et al. (2020) |  |  | ^ Anxiety score |
| Anitasari and Andrajiti (2017) |  | ^ Adherence to treatment, Haemoglobin levels |  |
| Atnafu et al. (2014) | ^ > 4 |  | ^ Service utilisation,  ^ ANC delivered by HEW or health professional |
| Ayiasi et al. (2016) | ^ >=3 |  | Adequate birth preparation |
| Bangal et al. (2017) | ^ > 4 | >=3 months’ supply, anaemia,  Tetanus toxoid injection, ultrasound examination | Maternal weight gain >10kg |
| Bogale et al. (2021) |  |  | ^ Worry and satisfaction with ANC |
| Carmichael et al. (2019) | ^ >=3 ANC at home |  |  |
| Chan et al. (2019) |  |  | ^ EPDS score, DASS score for anxiety and stress, SF-12 Physical and mental component |
| Chowdhury et al. (2019) |  |  | ^ Maternal healthcare knowledge and practice |
| Coleman et al. (2017) | >=4 |  |  |
| Coleman et al. (2020) | ^ >=4, >=2, >=3 |  |  |
| Eslami et al. (2018) |  | Fasting blood glucose, OGTT, GDM |  |
| Fedha et al. (2014) | ^ >= 4 | ^ Vitamin supplementation, ^ iron supplementation,  ^ first tetanus toxoid infection | ^ Diet and ^ HIV counselling,  ^ malaria prophylaxis and ^ deworming |
| Flueckiger et al. (2019) | ^ Proportion of 1, 2, 3, 4 visits |  | ^ Malaria prevention |
| Foster et al. (2017) | Full dose of ANC visits |  |  |
| Gerdts et al. (2019) |  |  | ^ Medical abortion preparedness |
| Gong et al. (2020) |  |  | ^ Prenatal depression |
| Guo et al. (2019) |  | ^ Glycaemic control compliance | Frequency of outpatient service |
| Ilozumba et al. (2018) | >=4 |  | Maternal knowledge (ANC and PNC) |
| Karamolahi et al. (2021) |  |  | Health literacy score |
| Kawakatsu et al. (2020) | ^ Attendance and timeliness |  |  |
| Khorshid et al. (2014) |  | ^ Iron supplementation compliance, anaemia |  |
| Kiani et al. (2021) |  |  | ^ Physical activity level, ^ perceived benefits |
| Lau et al. (2014) |  |  | ^ ANC knowledge |
| Li et al. (2020) | Rates of early pregnancy care |  |  |
| Lund et al. (2012, 2014a, 2014b) | ^ >=4 | Tetanus toxoid injection at two time-points | Malaria prevention |
| Mohamadirizi et al. (2014) |  |  | ^ Prenatal care awareness |
| Murthy et al. (2019, 2020) | >=3 | Calcium and iron/folic acid supplementation,  Tetanus toxoid injection |  |
| Ngoc et al. (2014) |  |  | ^ Rate of complete abortion,  ^ Rate of ongoing pregnancy |
| Oliveira et al. (2017) | ^ High ANC score, >= 6 ANC | Tetanus toxoid vaccine, HIV test, Syphilis test, Hepatitis B test, | Micronutrient supplementation, various immunisation and blood sample results |
| Onono et al. (2019) | ^ >=4 |  |  |
| Pai et al. (2013) |  | ^ Treatment adherence |  |
| Paratmanitya et al. (2021) | ^Timing of first ANC |  | ^ Change in pre-conceptional knowledge,  ^ Change in anthropometric measures (MUAC),  ^ Change in anthropometric measures (Weight) |
| Parsa et al. (2019) |  |  | Pre-eclampsia knowledge score |
| Prinja et al. (2017) | ^ >=3, ^Full ANC care | ^ Iron-Folic acid, ^ tetanus toxoid vaccine,  blood pressure, urine testing |  |
| Rani et al. (2022) |  |  | ^ Gestational weight gain, ^ weight retention |
| Ross et al. (2013) |  |  | ^ Depression rates, ^ minor depression score |
| Ruton et al. (2018) | ^ Rates |  |  |
| Sabin et al. (2020) | Attendance of all ANC |  |  |
| Shiferaw et al. (2016) | ^ >=4 |  |  |
| Singh et al. (2020) |  | ^ Change in Haemoglobin | ^ Weight gain |
| Souza et al. (2021) | ^ Prenatal adherence |  |  |
| Talebi et al. (2022) |  |  | ^ Physical activity level and weight during pregnancy |
| Tian et al. (2021) |  | ^ Glycaemic qualification rate |  |
| Vanhuyse et al. (2022) | ^ Attendance |  |  |
| Watterson et al. (2020) | ^ Attendance |  |  |
| Xie et al. (2018) | Early pregnancy visit | Folic acid supplementation, prenatal screening, Syphilis test, Hepatitis B test |  |
| Xuto et al. (2021) |  | Haemoglobin | Gestational weight gain, OGTT, State-anxiety |
| Zhang et al. (2019) |  | ^ GDM and hypertension | Gestational weight gain |

^ Primary outcome

|  | **Health Domain: Delivery Care** | | |
| --- | --- | --- | --- |
| **Study** | **Place of birth** | **EmOC** | **Delivery Others** |
| Akbarian et al. (2017) |  |  | ^ Hospital readmission |
| Amoakoh et al. (2019) |  |  | ^ Institutional neonatal mortality |
| Atnafu et al. (2014) | Proportion of home delivery |  |  |
| Ayiasi et al. (2016) | ^ Facility delivery |  | Birth preparation |
| Bangal et al. (2017) | ^ Facility delivery | Medical obstetric care | Low birth weight babies |
| Bellad et al. (2020) | ^ Delivery in facility with emergency care  ^ Facility births |  | Birth preparedness and complication readiness |
| Carmichael et al. (2019) | Facility delivery |  |  |
| Coleman et al. (2017) |  | Vaginal delivery | Low birth weight babies |
| Fedha et al. (2014) | ^ Place of birth discussed |  | ^ Intrauterine foetal death, ^ Immediate crying |
| Foster et al. (2017) | Facility delivery |  |  |
| Guo et al. (2019) |  | Vaginal delivery | Macrosomia, HbA1c before delivery |
| Hackett et al. (2018) | ^ Facility delivery |  |  |
| Ilozumba et al. (2018) | Facility delivery |  |  |
| Kassaye et al. (2016) | Facility delivery |  |  |
| Klokkenga et al. (2019) |  | ^ Postpartum haemorrhage and blood loss |  |
| Lund et al. (2012, 2014a, 2014b) | ^ SBA |  | Stillbirth |
| Lund et al. (2016) |  |  | Neonatal resuscitation knowledge and skills |
| Murthy et al. (2019, 2020) | Facility delivery |  | Birth weight >= 2.5kg |
| Omole et al. (2016) | ^ Facility delivery |  |  |
| Onono et al. (2019) |  |  | ^ Pregnancy outcomes (miscarriage/stillbirth)  ^ Status of mother after birth  ^ Time taken to facility |
| Prinja et al. (2017) | Institutional deliveries | Complication during and after delivery | Ambulance usage |
| Qureshi et al. (2020) | Delivery in facility with emergency care  Facility births |  | Birth preparedness and complication readiness |
| Ruton et al. (2018) | ^ Facility delivery |  | Malnutrition screening of child |
| Sabin et al. (2020) | Delivery in study hospital |  |  |
| Sevene et al. (2020) | Delivery in facility with emergency care  Facility births |  | Birth preparedness and complication readiness |
| Shiferaw et al. (2016) | ^ Institutional delivery |  |  |
| Unger et al. (2018) | ^ Facility delivery |  |  |
| Sumanova et al. (2021) |  | Oxytocin within 1 minute of delivery | Immediate newborn care, neonatal asphyxia,  stillbirth |
| Vanhuyse et al. (2022) | ^ Facility delivery |  |  |
| Von Dadelszen et al. (2020) | Delivery in facility with emergency care  Facility births |  | Birth preparedness and complication readiness |
| Xie et al. (2018) |  | Obstetric haemorrhage, caesarean delivery | Birth weight |
| Xuto et al. (2021) |  |  | Birth weight |
| Zhang et al. (2019) |  | Caesarean section | Maternal insulin level, cord blood C-peptide levels, birth weight, preterm birth, macrosomia |

^ Primary outcome

|  | **Health Domain: Postnatal Care (PNC)** | | | | |
| --- | --- | --- | --- | --- | --- |
| **Study** | **Number of PNC visits** | **Childhood immunisation** | **Breastfeeding practices** | **HIV / PMTCT** | **PNC Others** |
| Abuogi et al. (2022) |  |  |  | ^ Retention in HIV care 12 months postpartum |  |
| Adam et al. (2021) |  |  | ^ Short and long term EBF, early initiating of BF, bottle feeding, complementary food |  | Maternal knowledge |
| Araban et al. (2018) |  |  | ^ BF efficacy,  EBF 8 weeks postpartum |  |  |
| Atnafu et al. (2014) |  | Coverage |  |  |  |
| Atukunda et al. (2021) |  |  |  |  | ^ Use of contraception at 12 months postpartum |
| Ayiasi et al. (2016) |  |  | Appropriate practices |  | Cord and Thermal care, Newborn care |
| Bangal et al. (2017) | >=1 |  |  |  | Perinatal mortality |
| Bangure et al. (2015) |  | ^ coverage,  Delay in immunisation |  |  |  |
| Bellad et al. (2020) |  |  |  |  | ^ All-cause maternal mortality and morbidity,  ^ All-cause perinatal mortality and late neonatal mortality and morbidity |
| Bigna et al. (2014) |  |  |  | ^ HIV care attendance |  |
| Billah et al. (2022a, 2022b) |  |  | ^ Duration of EBF, early initiation of BF, colostrum feeding,  pre-lacteal feeding |  | Dietary diversity score for children 6-23 months of age |
| Brown et al. (2016) |  | ^ Completion rate |  |  |  |
| Carmichael et al. (2019) | ^ Home visit within 24h and 1 week | Full immunisation | Early initiation of EBF,  EBF 6 months postpartum |  |  |
| Chowdhury et al. (2019) |  |  |  |  | Newborn healthcare knowledge and practice |
| Coleman et al. (2017) |  |  |  | ^ Early infant diagnosis |  |
| Coleman et al. (2020) |  | ^ Fully immunised |  |  | *Complete continuum of care (ANC + immunisation)* |
| Dissieka et al. (2019) |  | ^ 4 vaccinations + Vit. A  Timeliness |  |  |  |
| Domek et al. (2019) |  | ^ Immunisation completion rates and timeliness |  |  |  |
| Dryden-Peterson et al. (2015) |  |  |  | ^ Complete phlebotomy,  ^ Initiation of ART |  |
| Ekhaguere et al. (2019) |  | ^ 3x pentavalent + 1x MCV  Timeliness |  |  |  |
| Eze and Adeleye. (2015) |  | Early receipt of DPT3 |  |  |  |
| Fahami et al. (2014) |  |  | ^BF awareness |  |  |
| Fedha et al. (2014) |  |  |  |  | ^ Neonatal death |
| Fikawati et al. (2019) |  |  | ^ EBF at 6 months |  |  |
| Flax et al. (2014) |  |  | ^ EBF for 1, 3, 6 months, early initiation, gave only colostrum |  |  |
| Foster et al. (2017) |  | DTP/MCV |  | ^ Retention in HIV care 12 months postpartum for infants and mothers, rapid HIV testing | Use of FP 1 year postpartum |
| Fotso et al. (2015a, 2015b) |  | Full vaccination by 12 months of age | ^ EBP at 6 months of age |  | Home-based and facility-based care for maternal health (ANC, delivery, PNC) |
| Garcia-Dia et al. (2016) |  | ^ Immunisation rates,  ^ Timeliness |  |  |  |
| Gibson et al. (2017) |  | ^ Full immunisation 12 months postpartum,  ^ Part immunisation,  ^ Measles |  |  |  |
| Harrington et al. (2019) |  |  |  |  | ^ Use of contraceptive 6w, 14w and 6months postpartum |
| Jerin et al. (2020) |  |  | ^ EBF rates, 1-5 months |  |  |
| Jiang et al. (2014, 2019) |  |  | ^ Duration of EBF |  | ^ BMI and WLZ at 12 months |
| Johri et al. (2020) |  | ^ Immunisation schedule knowledge |  |  |  |
| Kassaye et al. (2016) |  |  |  | ^ ART uptake of mother and child, infant HIV testing and transmission |  |
| Kawakatsu et al. (2020) |  | ^ Vaccination attendance and Timeliness |  |  | ^ Family planning attendance and timeliness |
| Kazi et al. (2018) |  | ^Immunisation at 18 weeks  Pentavalent 1, 2, & 3 |  |  |  |
| Kebaya et al. (2021) |  |  | EBF at 10 weeks | ^ Retention in care  Adherence to prophylaxis  Early infant diagnosis |  |
| Kebede et al. (2019) | ^ Attendance |  |  |  |  |
| Khodabandah et al. (2017) |  |  |  |  | ^Physical activity, ^ diet |
| Kinuthia et al. (2021) |  |  | EBF duration | ^ Maternal non-suppression,  ^ infant HIV infection/death, Maternal HIV adherence | Postpartum contraception |
| Levine et al. (2021) |  | ^ OPV and BCG |  |  |  |
| Li et al. (2020) | 1 postpartum visit |  |  |  | Satisfaction rate of postpartum service |
| Lund et al. (2012, 2014a, 2014b) |  |  |  |  | Perinatal mortality.  Mortality (<42days) |
| Lund et al. (2016) |  |  |  |  | ^ Perinatal death |
| Martinez-Fernandez et al. (2015) |  |  |  |  | ^ Maternal mortality ratio,  ^ Infant mortality ratio |
| Maslowsky et al. (2016) | ^ 1 and 6 weeks postpartum |  | ^ EBF 3 months postpartum,  ^ Infant formula use |  | ^ Use of contraception 3 months postpartum |
| Masoi et al. (2019) |  |  |  |  | ^ Knowledge on obstetric and newborn danger signs |
| Modi et al. (2017, 2019) | >=2 (at home) within 1 week |  |  |  | ^ MACCI |
| Murthy et al. (2019, 2020) |  | Full immunisation | Early initiation of BF,  colostrum feeding |  | Malnourishment at 1 year |
| Nagar et al. (2017) |  | ^ 3 DTP |  |  |  |
| Nemerimana et al. (2021) |  |  |  |  | ^ LAZ, ^ WLZ, ^ WAZ |
| Nguyet et al. (2021) |  |  | ^ EBF 1-4 days postpartum,  ^ EBF 4 weeks postpartum |  | Maternal confidence |
| Nordberg et al. (2021) |  |  |  | ^ Early infant diagnosis |  |
| Odeny et al. (2014) |  |  |  | ^ Postpartum retention in PMTCT, ^ Infant HIV testing |  |
| Odeny et al. (2019) |  |  |  | ^ HIV testing  ^ Postpartum retention |  |
| Oladepo et al. (2020) |  | ^ BCG, ^ DTP1, ^ DTP2, ^ DTP3, OPV1, 2, 3, HBV, IPV, MCV, Yellow fever |  |  |  |
| Olajubu et al. (2020) | ^ >4 (1, 2, 3, 4) |  |  |  |  |
| Onono et al. (2019) | ^ >=4 |  |  |  |  |
| Prieto et al. (2017) |  |  | ^ EBF recommendation awareness |  |  |
| Prinja et al. (2017) |  | Full immunisation |  |  |  |
| Qureshi et al. (2020) |  |  |  |  | ^ All-cause mortality and ^ morbidity,  ^ All-cause neonatal mortality and morbidity |
| Reiss et al. (2019) |  |  |  |  | LARC post MR |
| Ruton et al. (2018) | ^ Rates |  |  |  |  |
| Sabin et al. (2020) | Attendance of all PNC |  |  |  | ^ Full retention (ANC/PNC, ART medication, delivery in study hospital) |
| Sarmiento et al. (2019) |  |  |  |  | ^ GDM follow up |
| Schwartz et al. (2015) |  |  |  | ART retention at 12 months  Infant PCR HIV testing |  |
| Seth et al. (2018) |  | ^ Immunisation coverage Timeliness |  |  |  |
| Sevene et al. (2020) |  |  |  |  | ^ All-cause mortality and ^ morbidity,  ^ All-cause neonatal mortality and morbidity |
| Seyyedi et al. (2020) |  |  |  |  | ^ Change in WHZ,  Maternal nutritional literacy  HAZ and WAZ |
| Seyyedi et al. (2021) |  |  | ^ Self-efficacy |  | ^ KAP score knowledge, attitudes, practice, overall |
| Shaaban et al. (2020) |  |  |  |  | ^ LARC initiation |
| Shiferaw et al. (2016) | ^ Any PNC in health centres |  |  |  |  |
| Short et al. (2020) |  |  | ^ EBF at 6 months (1 to 6),  ^ Early initiation,  ^ Colostrum given,  ^ Pre-lacteal and top-feeding,  ^ Supplementary feeding |  |  |
| Simonyan et al. (2013) |  |  |  |  | ^ Healthcare utilisation,  Number of disease episodes |
| Smith et al. (2015) |  |  |  |  | Effective contraception 4 and 12 months after abortion |
| Sun et al. (2021) |  |  |  |  | ^ Postnatal depression |
| Tahir and Al-Sadat (2013) |  |  | ^ EBF 1, 4 and 6 months postpartum |  |  |
| Tian et al. (2021) |  |  |  |  | OGTT, Postpartum diabetes |
| Uddin et al. (2016) |  | ^ Full vaccination coverage,  ^ Age-appropriate coverage |  |  |  |
| Ugwa et al. (2020) |  |  |  |  | ^ Maternal and child health knowledge |
| Unger et al. (2018) |  |  | ^ EBF at 10, 16, 24 weeks |  | ^ Contraceptive use at 10, 16, 24 weeks |
| Vanhuyse et al. (2022) | ^ >=1 between 4 and 12 months | ^ Attendance |  |  |  |
| Von Dadelszen et al. (2020) |  |  |  |  | ^ All-cause mortality and ^ morbidity  ^ All-cause neonatal mortality and morbidity |
| Wu et al. (2020) |  |  | ^ EBF at 0, 1, 2, 3, 4, 5 months,  Early initiation,  Pre-lacteal feeding rate |  | Knowledge of BF |
| Xie et al. (2018) |  |  |  |  | Maternal and perinatal death  Thyroid test, hearing test, Phenylketonuria test, |
| Zhou et al. (2016) |  |  |  |  | ^ Micronutrient powder supplementation compliance,  Anaemia (6-12 months old) |
| Zhou et al. (2020) |  |  |  |  | SGA or Macrosomia |
| Zhuo et al. (2022) |  |  |  |  | ^ Medication adherence (diabetes management) |
| Zurovac et al. (2011) |  |  |  |  | ^ Malaria case management in (21-26 months old) |

^ Primary outcome
