# Supplementary material for: Impact of mHealth interventions on maternal, newborn, and child health from conception to 24 months postpartum in low- and middle-income countries: a systematic review
Source: BMC Med. 2024 May 15;22:196. doi: 10.1186/s12916-024-03417-9 (PMC11095039; doi:10.1186/s12916-024-03417-9)
Supplement: Supplementary file 4 — Additional file 4: Tables A4a and A4b Descriptive review and results [file 12916_2024_3417_MOESM4_ESM.docx]

**Table S3**  Characteristics of the reviewed studies

| Author | Year | Country | Study Type^*^ | mHealth function^**^ | mHealth form^***^ | ANC related outcomes | | | Birth related outcomes | | | PNC related outcomes | | | | | QA |
| --- | --- | --- | --- | --- | --- | --- | --- | --- | --- | --- | --- | --- | --- | --- | --- | --- | --- |
|  |  |  |  |  |  | **Visit** | **Tests**^†^ | **Other** | **Place** | **EmOC** | **Other** | **Visit** | **IMM** | **Feeding** | **HIV** | **Other** | |
| Abbaspoor et al. | 2020 | Iran | RCT | 1 | 1 |  |  | X |  |  |  |  |  |  |  |  | M |
| Abuogi et al. | 2022 | Kenya | C-RCT | 1 | 1, 2 |  |  |  |  |  |  |  |  |  | X |  | M |
| Adam et al. | 2021 | South Africa | C-RCT | 1, 4 | 1 |  |  |  |  |  |  |  |  | X |  | X | H |
| Akbarian et al. | 2017 | Iran | RCT | 2 | 1 |  |  |  |  |  | X |  |  |  |  |  | SC |
| Aksoy Derya et al. | 2020 | Turkey | QES | 1 | 1, 2 |  |  | X |  |  |  |  |  |  |  |  | 9/9 |
| Amoakoh et al. | 2019 | Ghana | C-RCT | 1, 6 | 1, 2 |  |  |  |  |  | X |  |  |  |  |  | L |
| Anitasari and Andrajiti | 2017 | Indonesia | QES | 1 | 1 |  | X |  |  |  |  |  |  |  |  |  | 7/9 |
| Araban et al. | 2018 | Iran | RCT | 1 | 1 |  |  |  |  |  |  |  |  | X |  |  | L |
| Atnafu et al. | 2017 | Ethiopia | RCT | 3, 4, 7, 8 | 1, 2 | X |  | X | X |  |  |  | X |  |  |  | L |
| Atukunda et al. | 2021 | Uganda | RCT | 1 | 1 |  |  |  |  |  |  |  |  |  |  | X | L |
| Ayiasi et al. | 2016 | Uganda | RCT | 7 | 2 | X |  | X | X |  |  |  |  | X |  | X | L |
| Bangal et al. | 2017 | India | RCT | 1 | 1, 2 | X | X | X | X |  |  | X |  |  |  | X | H |
| Bangure et al. | 2015 | Zimbabwe | RCT | 1 | 1 |  |  |  |  |  |  |  | X |  |  |  | L |
| Bellad et al. | 2020 | India | C-RCT | 3, 4, 5, 6 | 1 |  |  |  | X |  | X |  |  |  |  | X | L |
| Bigna et al. | 2014 | Cameroon | RCT | 1 | 1, 2 |  |  |  |  |  |  |  |  |  | X |  | L |
| Billah et al. | 2022a,b | Bangladesh | C-RCT | 1, 3, 4, 8 | 1 |  |  |  |  |  |  |  |  | X |  | X | M |
| Bogale et al. | 2021 | Palestine | C-RCT | 1 | 1 |  |  | X |  |  |  |  |  |  |  |  | L |
| Brown et al. | 2016 | Nigeria | RCT | 1, 9 | 2 |  |  |  |  |  |  |  | X |  |  |  | M |
| Carmichael et al. | 2019 | India | C-RCT | 1, 4, 6, 8 | 1 | X |  |  | X |  |  | X | X | X |  |  | L |
| Chan et al. | 2019 | Hong Kong | RCT | 1 | 1, 2 |  |  | X |  |  |  |  |  |  |  |  | M |
| Chowdhury et al. | 2019 | India | QES | 1 | 1 |  |  | X |  |  |  |  |  |  |  | X | 6/9 |
| Coleman et al. | 2017 | South Africa | QES | 1 | 1 | X |  |  |  | X | X |  |  |  | X |  | 7/9 |
| Coleman et al. | 2020 | South Africa | QES | 1 | 1 | X |  |  |  |  |  |  | X |  |  | X | 8/9 |
| Dissieka et al. | 2019 | Côte d’Ivoire | RCT | 1 | 1 |  |  |  |  |  |  |  | X |  |  |  | L |
| Domek et al. | 2019 | Guatemala | RCT | 1, 5 | 1 |  |  |  |  |  |  |  | X |  |  |  | L |
| Dryden-Peterson et al. | 2015 | Botswana | C-RCT | 7 | 1 |  |  |  |  |  |  |  |  |  | X |  | M |
| Ekhaguere et al. | 2019 | Nigeria | RCT | 1 | 1 |  |  |  |  |  |  |  | X |  |  |  | L |
| Eslami et al. | 2018 | Iran | RCT | 1 | 1 |  | X |  |  |  |  |  |  |  |  |  | L |
| Eze and Adeleye | 2015 | Nigeria | RCT | 1 | 1 |  |  |  |  |  |  |  | X |  |  |  | H |
| Fahami et al. | 2014 | Iran | QES | 1 | 1 |  |  |  |  |  |  |  |  | X |  |  | 7/9 |
| Fedha et al. | 2014 | Kenya | RCT | 1 | 1, 2 | X | X | X | X |  | X |  |  |  |  | X | M |
| Fikawati et al. | 2019 | Pakistan | QES | 1 | 1, 2 |  |  |  |  |  |  |  |  | X |  |  | 7/9 |
| Flax et al. | 2014 | Nigeria | C-RCT | 1 | 1 |  |  |  |  |  |  |  |  | X |  |  | M |
| Flueckiger et al. | 2019 | Guinea | RCT | 1 | 1 | X |  | X |  |  |  |  |  |  |  |  | H |
| Foster et al. | 2017 | Zimbabwe | C-RCT | 1 | 1 | X |  |  | X |  |  |  | X | X |  | X | M |
| Fotso et al. | 2015ab | Malawi | QES | 1 | 1, 2 |  |  |  |  |  |  |  | X | X |  | X | 6/9 |
| Garcia-Dia et al. | 2016 | Philippines | RCT | 1 | 1 |  |  |  |  |  |  |  | X |  |  |  | M |
| Gerdts et al. | 2019 | Indonesia | RCT | 1 | 1 |  |  | X |  |  |  |  |  |  |  |  | M |
| Gibson et al. | 2017 | Kenya | C-RCT | 1 | 1 |  |  |  |  |  |  |  | X |  |  |  | M |
| Gong et al. | 2020 | China | QES | 1 | 1 |  |  | X |  |  |  |  |  |  |  |  | 7/9 |
| Guo et al. | 2019 | China | RCT | 1 | 2 |  | X | X |  | X | X |  |  |  |  |  | L |
| Hackett et al. | 2018 | Tanzania | C-RCT | 1, 4, 6, 8 | 1 |  |  |  | X |  |  |  |  |  |  |  | M |
| Harrington et al. | 2019 | Kenya | RCT | 1 | 2 |  |  |  |  |  |  |  |  |  |  | X | L |
| Ilozumba et al. | 2018 | India | QES | 1, 4, 6, 8 | 1 | X |  | X | X |  |  |  |  |  |  |  | 7/9 |
| Jerin et al. | 2020 | Bangladesh | QES | 1 | 2 |  |  |  |  |  |  |  |  | X |  |  | 9/9 |
| Jiang et al. | 2014/19 | China | QES | 1 | 1, 2 |  |  |  |  |  |  |  |  | X |  | X | 6/9 |
| Johri et al. | 2020 | India | C-RCT | 1 | 1, 2, 3 |  |  |  |  |  |  |  | X |  |  |  | L |
| Karamolahi et al. | 2021 | Iran | RCT | 1 | 1 |  |  | X |  |  |  |  |  |  |  |  | M |
| Kassaye et al. | 2016 | Kenya | C-RCT | 1, 9 | 1, 2 |  |  |  | X |  |  |  |  |  | X |  | M |
| Kawakatsu et al. | 2020 | Nigeria | RCT | 1 | 1, 2 | X |  |  |  |  |  |  | X |  |  | X | M |
| Kazi et al. | 2018 | Pakistan | RCT | 1 | 1 |  |  |  |  |  |  |  | X |  |  |  | L |
| Kebaya et al. | 2021 | Kenya | RCT | 1 | 2 |  |  |  |  |  |  |  |  | X | X |  | M |
| Kebede et al. | 2019 | Ethiopia | C-RCT | 1 | 1 | X |  |  |  |  |  | X |  |  |  |  | L |
| Khodabandeh et al. | 2017 | Iran | RCT | 1 | 1, 2 |  |  |  |  |  |  |  |  |  |  | X | L |
| Khorshid et al. | 2014 | Iran | RCT | 1 | 1 |  | X |  |  |  |  |  |  |  |  |  | M |
| Kiani et al. | 2021 | Iran | QES | 1 | 1 |  |  | X |  |  |  |  |  |  |  |  | 9/9 |
| Kinuthia et al. | 2021 | Kenya | RCT | 1 | 1, 2 |  |  |  |  |  |  |  |  | X | X | X | L |
| Klokkenga et al. | 2019 | Ghana | C-RCT | 6, 9 | 1 |  |  |  |  | X |  |  |  |  |  |  | M |
| Lau et al. | 2014 | South Africa | RCT | 1 | 1 |  |  | X |  |  |  |  |  |  |  |  | H |
| Levine et al. | 2021 | Ghana | C-RCT | 1, 3, 7 | 2 |  |  |  |  |  |  |  | X |  |  |  | H |
| Li et al. | 2020 | China | RCT | 1 | 1, 2 | X |  |  |  |  |  | X |  |  |  | X | L |
| Lund et al. | 2012 2014a,b | Tanzania | C-RCT | 1 | 1, 2 | X | X | X | X |  | X |  |  |  |  | X | L |
| Lund et al. | 2016 | Ethiopia | C-RCT | 6, 9 | 1 |  |  |  |  |  | X |  |  |  |  | X | M |
| Martinez-Fernandez et al. 2015 | | Guatemala | QES | 3, 4, 7, 9 | 2 |  |  |  |  |  |  |  |  |  |  | X | 7/9 |
| Maslowsky et al. | 2016 | Ecuador | QES | 1 | 2 |  |  |  |  |  |  | X |  | X |  | X | 7/9 |
| Masoi et al. | 2019 | Tanzania | QES | 1 | 2 |  |  |  |  |  |  |  |  |  |  | X | 9/9 |
| Modi et al. | 2017/19 | India | C-RCT | 1,3,4,5,6, 7,8,10,11 | 2 |  |  |  |  |  |  | X |  |  |  | X | M |
| Mohamadirizi et al. | 2014 | Iran | QES | 1 | 1 |  |  | X |  |  |  |  |  |  |  |  | 9/9 |
| Murthy et al. | 2019/20 | India | QES | 1 | 1 | X | X |  | X |  | X |  | X | X |  | X | 7/9 |
| Nagar et al. | 2018 | India | C-RCT | 1, 4, 5 | 1 |  |  |  |  |  |  |  |  |  |  |  | M |
| Nemerimana et al. | 2021 | Rwanda | QES | 4 | 1 |  |  |  |  |  |  |  |  |  |  | X | 9/9 |
| Ngoc et al. | 2014 | Vietnam | RCT | 2 | 2 |  |  | X |  |  |  |  |  |  |  |  | H |
| Nguyet et al. | 2021 | Vietnam | QES | 1 | 1 |  |  |  |  |  |  |  |  | X |  | X | 8/9 |
| Nordberg et al. | 2021 | Kenya | RCT | 1 | 2 |  |  |  |  |  |  |  |  |  | X |  | L |
| Odeny et al. | 2014 | Kenya | RCT | 1 | 2 |  |  |  |  |  |  |  |  |  | X |  | M |
| Odeny et al. | 2019 | Kenya | C-RCT | 1 | 2 |  |  |  |  |  |  |  |  |  | X |  | M |
| Oladepo et al. | 2020 | Nigeria | QES | 1 | 1 |  |  |  |  |  |  |  | X |  |  |  | 6/9 |
| Olajubu et al. | 2020 | Nigeria | QES | 1 | 1 |  |  |  |  |  |  | X |  |  |  |  | 6/9 |
| Oliveira et al. | 2017 | Brazil | C-RCT | 1 | 1, 2 | X | X | X |  |  |  |  |  |  |  |  | L |
| Omole et al. | 2016 | Nigeria | C-RCT | 1 | 1, 2 |  |  |  | X |  |  |  |  |  |  |  | M |
| Onono et al. | 2019 | Kenya | QES | 1 | 2 | X |  |  |  |  | X | X |  |  |  |  | 6/9 |
| Pai et al. | 2013 | India | RCT | 1 | 1 |  | X |  |  |  |  |  |  |  |  |  | H |
| Paratmanitya et al. | 2021 | Indonesia | C-RCT | 1 | 2 | X |  | X |  |  |  |  |  |  |  |  | M |
| Parsa et al. | 2019 | Iran | QES | 1 | 1 |  |  | X |  |  |  |  |  |  |  |  | 9/9 |
| Prieto et al. | 2017 | Guatemala | QES | 1 | 1, 2 |  |  |  |  |  |  |  |  | X |  |  | 7/9 |
| Prinja et al. | 2017 | India | QES | 1, 5, 6, 8 | 1, 2 | X | X |  | X | X | X |  |  |  |  |  | 9/9 |
| Qureshi et al. | 2020 | Pakistan | C-RCT | 3, 4, 5, 6 | 1 |  |  |  | X |  | X |  |  |  |  | X | L |
| Rani et al. | 2022 | India | RCT | 1 | 1 |  |  | X |  |  |  |  |  |  |  |  | L |
| Reiss et al. | 2019 | Bangladesh | RCT | 1 | 1 |  |  |  |  |  |  |  |  |  |  | X | M |
| Ross et al. | 2013 | Thailand | RCT | 1 | 2 |  |  | X |  |  |  |  |  |  |  |  | H |
| Ruton et al. | 2018 | Rwanda | QES | 4, 5, 7, 8 | 2 | X |  |  | X |  | X | X |  |  |  |  | 7/9 |
| Sabin et al. | 2020 | Uganda | RCT | 1 | 1 | X |  |  | X |  |  | X |  |  |  | X | L |
| Sarmiento et al. | 2019 | Philippines | RCT | 1 | 1 |  |  |  |  |  |  |  |  |  |  | X | H |
| Schwartz et al. | 2015 | South Africa | QES | 1 | 1, 2 |  |  |  |  |  |  |  |  |  | X |  | 7/9 |
| Seth et al. | 2018 | India | RCT | 1 | 1 |  |  |  |  |  |  |  | X |  |  |  | M |
| Sevene et al. | 2020 | Mozambique | C-RCT | 3, 4, 5, 6 | 1 |  |  |  | X |  | X |  |  |  |  | X | L |
| Seyyedi et al. | 2020 | Iran | RCT | 1 | 1 |  |  |  |  |  |  |  |  |  |  | X | L |
| Seyyedi et al. | 2021 | Iran | RCT | 1 | 1, 2 |  |  |  |  |  |  |  |  | X |  | X | L |
| Shaaban et al. | 2020 | Egypt | RCT | 1 | 2 |  |  |  |  |  |  |  |  |  |  | X | L |
| Shiferaw et al. | 2016 | Ethiopia | QES | 4, 5, 8, 9 | 1, 2 | X |  |  | X |  |  | X |  |  |  |  | 7/9 |
| Short et al. | 2020 | India | QES | 1, 9 | 1 |  |  |  |  |  |  |  |  | X |  |  | 9/9 |
| Simonyan et al. | 2013 | Mali | QES | 4, 5 | 2 |  |  |  |  |  |  |  |  |  |  | X | 7/9 |
| Singh et al. | 2020 | Nepal | C-RCT | 1 | 1 |  | X | X |  |  |  |  |  |  |  |  | L |
| Smith et al. | 2015 | Cambodia | RCT | 1 | 1, 2 |  |  |  |  |  |  |  |  |  |  | X | M |
| Souza et al. | 2021 | Brazil | RCT | 1 | 1, 2 | X |  |  |  |  |  |  |  |  |  |  | L |
| Sun et al. | 2021 | China | RCT | 1 | 1, 2 |  |  |  |  |  |  |  |  |  |  | X | L |
| Tahir and Al-Sadat | 2013 | Malaysia | RCT | 1 | 2 |  |  |  |  |  |  |  |  | X |  |  | L |
| Talebi et al. | 2020 | Iran | RCT | 1 | 2 |  |  | X |  |  |  |  |  |  |  |  | M |
| Tian et al, Huang et al. | 2021/21 | China | RCT | 1 | 2 |  | X |  |  |  |  |  |  |  |  | X | M |
| Uddin et al. | 2016 | Bangladesh | QES | 1, 3 | 1 |  |  |  |  |  |  |  | X |  |  |  | 7/9 |
| Ugwa et al. | 2020 | Nigeria | C-RCT | 9 | 1, 2 |  |  |  |  |  |  |  |  |  |  | X | L |
| Unger et al. | 2018 | Kenya | RCT | 1 | 1, 2 |  |  |  | X |  |  |  |  | X |  | X | M |
| Vanhuyse et al. | 2022 | Kenya | C-RCT | 12 | 1 | X |  |  | X |  |  | X | X |  |  |  | L |
| Von Dadelszen et al. | 2020 | Mozambique, Pakistan, India | C-RCT | 3, 4, 5, 6 | 1 |  |  |  | X |  | X |  |  |  |  | X | L |
| Watterson et al. | 2020 | Samoa | QES | 1 | 1 | X |  |  |  |  |  |  |  |  |  |  | 4/9 |
| Wu et al. | 2020 | China | RCT | 1 | 1 |  |  |  |  |  |  |  |  | X |  |  | M |
| Xie et al. | 2018 | China | C-RCT | 1 | 1 | X | X |  |  | X | X |  |  |  |  | X | H |
| Xuto et al. | 2021 | Thailand | RCT | 1 | 1 |  | X | X |  |  | X |  |  |  |  |  | M |
| Zhang et al. | 2019 | China | RCT | 1 | 1, 2 |  | X | X |  | X | X |  |  |  |  |  | L |
| Zhou et al. | 2016 | China | C-RCT | 1 | 1 |  |  |  |  |  |  |  |  |  |  | X | M |
| Zhou et al. | 2020 | China | QES | 1 | 1 |  |  |  |  |  |  |  |  |  |  | X | 7/9 |
| Zhuo et al. | 2022 | China | RCT | 1 | 2 |  |  |  |  |  |  |  |  |  |  | X | M |
| Zurovac et al. | 2011 | Kenya | C-RCT | 6, 9 | 1 |  |  |  |  |  |  |  |  |  |  | X | L |

^*^Study type: RCT, randomised controlled trial; C-RCT, cluster randomised controlled trial; QES, Quasi-experimental study

^**^mHealth Function is the categorisation of mHealth applications as described by Labrique et al.

^***^ mHealth form: 1=unidirectional communication, 2=two-way communication, 3=multi-directional communication

^†^ Test, injections

Abbreviations: EmOC, emergency obstetric care; QA, quality assessment; H, high risk; IMM, immunisation; L, low risk; M, Moderate risk; QA, quality assessment
