# Supplementary material for: Impact of mHealth interventions on maternal, newborn, and child health from conception to 24 months postpartum in low- and middle-income countries: a systematic review
Source: BMC Med. 2024 May 15;22:196. doi: 10.1186/s12916-024-03417-9 (PMC11095039; doi:10.1186/s12916-024-03417-9)
Supplement: Supplementary file 5 — Additional file 5: Table A5a and A5b Quality assessment results [file 12916_2024_3417_MOESM5_ESM.docx]

**Table A5a** Risk of bias assessment of included studies based on the criteria of the Cochrane Handbook (RCTs and Cluster RCTs)

|  | Randomised controlled trials | Domain 1 | | Domain 2 | Domain 3 | Domain 4 | Domain 5 | Overall grade |
| --- | --- | --- | --- | --- | --- | --- | --- | --- |
| 1 | Abbaspoor et al. (2020) | Low risk | | Low risk | Some concerns | Some concerns | Low risk | Some concerns |
| 2 | Akbarian et al. (2017) | Some concerns | | Low risk | Low risk | Low risk | Low risk | Some concerns |
| 3 | Araban et al. (2018) | Low risk | | Low risk | Low risk | Low risk | Low risk | Low risk |
| 4 | Atnafu et al. (2017) | Low risk | | Low risk | Low risk | Low risk | Low risk | Low risk |
| 5 | Atukunda et al. (2021) | Low risk | | Low risk | Low risk | Low risk | Low risk | Low risk |
| 6 | Ayiasi et al. (2016) | Low risk | | Low risk | Low risk | Low risk | Low risk | Low risk |
| 7 | Bangal et al. (2017) | High risk | | Some concerns | Low risk | Low risk | Some concerns | High risk |
| 8 | Bangure et al. (2015) | Low risk | | Low risk | Low risk | Low risk | Low risk | Low risk |
| 9 | Bigna et al. (2014) | Low risk | | Low risk | Low risk | Low risk | Low risk | Low risk |
| 10 | Chan et al. (2019) | Low risk | | Low risk | Some concerns | Low risk | Low risk | Some concerns |
| 11 | Dissieka et al. (2019) | Low risk | | Low risk | Low risk | Low risk | Low risk | Low risk |
| 12 | Domek et al. (2019) | Low risk | | Low risk | Low risk | Low risk | Low risk | Low risk |
| 13 | Ekhaguere et al. (2019) | Low risk | | Low risk | Low risk | Low risk | Low risk | Low risk |
| 14 | Eslami et al. (2018) | Low risk | | Low risk | Low risk | Low risk | Low risk | Low risk |
| 15 | Eze and Adeleye (2020) | Some concerns | | Some concerns | Some concerns | Low risk | Low risk | High risk |
| 16 | Fedha et al. (2014) | Some concerns | | Some concerns | Low risk | Low risk | Low risk | Some concerns |
| 17 | Flueckiger et al. (2019) | High risk | | Low risk | Low risk | Low risk | Low risk | High risk |
| 18 | Garcia-Dia et al. (2016) | Some concerns | | Some concerns | Low risk | Low risk | Low risk | Some concerns |
| 19 | Gerdts et al. (2019) | Low risk | | Low risk | Some concerns | Low risk | Low risk | Some concerns |
| 20 | Guo et al. (2019) | Low risk | | Low risk | Low risk | Low risk | Low risk | Low risk |
| 21 | Harrington et al. (2019) | Low risk | | Low risk | Low risk | Low risk | Low risk | Low risk |
| 22 | Karamolahi et al. (2021) | Some concerns | | Low risk | Low risk | Some concerns | Low risk | Some concerns |
| 23 | Kawakatsu et al. (2020) | Some concerns | | Low risk | Low risk | Low risk | Low risk | Some concerns |
| 24 | Kazi et al. (2018) | Low risk | | Low risk | Low risk | Low risk | Low risk | Low risk |
| 25 | Kebaya et al. (2021) | Low risk | | Low risk | Low risk | Some concerns | Low risk | Some concerns |
| 26 | Khodabandeh et al. (2017) | Low risk | | Low risk | Low risk | Low risk | Low risk | Low risk |
| 27 | Khorshid et al. (2014) | Low risk | | Some concerns | Some concerns | Low risk | Low risk | Some concerns |
| 28 | Kinuthia et al. (2021) | Low risk | | Low risk | Low risk | Low risk | Low risk | Low risk |
| 29 | Lau et al. (2014) | Some concerns | | High risk | High risk | Some concerns | Some concerns | High risk |
| 30 | Li et al. (2020) | Low risk | | Low risk | Low risk | Low risk | Low risk | Low risk |
| 31 | Ngoc et al. (2014) | Low risk | | Some concerns | High risk | Some concerns | Some concerns | High risk |
| 32 | Nordberg et al. (2021) | Low risk | | Low risk | Low risk | Low risk | Low risk | Low risk |
| 33 | Odeny et al. (2014) | Low risk | | Some concerns | Low risk | Low risk | Low risk | Some concerns |
| 34 | Pai et al. (2013) | Low risk | | Low risk | High risk | Low risk | Low risk | High risk |
| 35 | Rani et al. (2022) | Low risk | | Low risk | Low risk | Low risk | Low risk | Low risk |
| 36 | Reiss et al. (2019) | Low risk | | Low risk | Some concerns | Low risk | Low risk | Some concerns |
| 37 | Ross et al. (2013) | High risk | | Low risk | Low risk | Low risk | Low risk | High risk |
| 38 | Sabin et al. (2020) | Low risk | | Low risk | Low risk | Low risk | Low risk | Low risk |
| 39 | Sarmiento et al. (2019) | Low risk | | Low risk | High risk | Low risk | Low risk | High risk |
| 40 | Seth et al. (2018) | Low risk | | Low risk | Some concerns | Low risk | Low risk | Some concerns |
| 41 | Seyyedi et al. (2020) | Low risk | | Low risk | Low risk | Low risk | Low risk | Low risk |
| 42 | Seyyedi et al. (2021) | Low risk | | Low risk | Low risk | Low risk | Low risk | Low risk |
| 43 | Shaaban et al. (2020) | Low risk | | Low risk | Low risk | Low risk | Low risk | Low risk |
| 44 | Smith et al. (2015) | Low risk | | Low risk | Some concerns | Low risk | Low risk | Some concerns |
| 45 | Souza et al. (2021) | Low risk | | Low risk | Low risk | Low risk | Low risk | Low risk |
| 46 | Sun et al. (2021) | Low risk | | Low risk | Low risk | Low risk | Low risk | Low risk |
| 47 | Tahir and Al-Sadat (2013) | Low risk | | Low risk | Low risk | Low risk | Low risk | Low risk |
| 48 | Talebi et al. (2022) | Low risk | | Low risk | Some concerns | Some concerns | Low risk | Some concerns |
| 49-50 | Tian et al. (2021) and Huang et al. (2021) | Low risk | | Low risk | Some concerns | Low risk | Low risk | Some concerns |
| 51 | Unger et al. (2018) | Low risk | | Low risk | Some concerns | Low risk | Low risk | Some concerns |
| 52 | Wu et al. (2020) | Low risk | | Low risk | Some concerns | Low risk | Low risk | Some concerns |
| 53 | Xuto et al. (2022) | Low risk | | Low risk | Some concerns | Low risk | Low risk | Some concerns |
| 54 | Zhang et al. (2019) | Low risk | | Low risk | Low risk | Low risk | Low risk | Low risk |
| 55 | Zhuo et al. (2022) | Some concerns | | Low risk | Low risk | Some concerns | Low risk | Some concerns |
|  | **Cluster randomised controlled trials** | **Domain 1a** | **Domain 1b** | **Domain 2** | **Domain 3** | **Domain 4** | **Domain 5** | **Overall grade** |
| 1 | Abuogi et al. (2022) | Low risk | Low risk | Low risk | Some concerns | Some concerns | Low risk | Some concerns |
| 2 | Adam et al. (2021) | Low risk | Low risk | Low risk | High risk | Low risk | Low risk | High risk |
| 3 | Amoakoh et al. (2019) | Low risk | Low risk | Low risk | Low risk | Low risk | Low risk | Low risk |
| 4 | Bellad et al. (2020) | Low risk | Low risk | Low risk | Low risk | Low risk | Low risk | Low risk |
| 5-6 | Billah et al. (2022a and 2022b) | Low risk | Low risk | Low risk | Low risk | Some concerns | Low risk | Some concerns |
| 7 | Bogale et al. (2021) | Low risk | Low risk | Low risk | Low risk | Low risk | Low risk | Low risk |
| 8 | Brown et al. (2016) | Low risk | Low risk | Some concerns | Low risk | Some concerns | Low risk | Some concerns |
| 9 | Carmichael et al. (2019) | Low risk | Low risk | Low risk | Low risk | Low risk | Low risk | Low risk |
| 10 | Dryden-Peterson et al. (2015) | Low risk | Low risk | Low risk | Some concerns | Low risk | Low risk | Some concerns |
| 11 | Flax et al. (2014) | Some concerns | Low risk | Some concerns | Low risk | Low risk | Low risk | Some concerns |
| 12 | Foster et al. (2017) | Low risk | Some concerns | Low risk | Some concerns | Low risk | Low risk | Some concerns |
| 13 | Gibson et al. (2017) | Low risk | Low risk | Some concerns | Low risk | Low risk | Low risk | Some concerns |
| 14 | Hackett et al. (2018) | Low risk | Low risk | Low risk | Some concerns | Low risk | Low risk | Some concerns |
| 15 | Johri et al. (2020) | Low risk | Low risk | Low risk | Low risk | Low risk | Low risk | Low risk |
| 16 | Kassaye et al. (2016) | Some concerns | Low risk | Some concerns | Low risk | Low risk | Some concerns | High risk |
| 17 | Kebede et al. (2019) | Low risk | Low risk | Low risk | Low risk | Low risk | Low risk | Low risk |
| 18 | Klokkenga et al. (2019) | Some concerns | Low risk | Low risk | Low risk | Low risk | Low risk | Some concerns |
| 19 | Levine et al. (2021) | High risk | Some concerns | Some concerns | Low risk | Low risk | Low risk | High risk |
| 20-22 | Lund et al. (2012, 2014a, 2014b) | Low risk | Low risk | Low risk | Low risk | Low risk | Low risk | Low risk |
| 23 | Lund et al. (2016) | Low risk | Low risk | Some concerns | Some concerns | Low risk | Low risk | Some concerns |
| 24-25 | Modi et al. (2017 and 2019) | Low risk | Low risk | Some concerns | Low risk | Low risk | Low risk | Some concerns |
| 26 | Nagar et al. (2017) | Some concerns | Low risk | Some concerns | Low risk | Low risk | Low risk | Some concerns |
| 27 | Odeny et al. (2019) | Low risk | Some concerns | Low risk | Low risk | Some concerns | Low risk | Some concerns |
| 28 | Oliveira et al. (2017) | Low risk | Low risk | Low risk | Low risk | Low risk | Low risk | Low risk |
| 29 | Omole et al. (2016) | Some concerns | Low risk | Low risk | Low risk | Low risk | Low risk | Some concerns |
| 30 | Paratmanitya et al. (2021) | Low risk | Low risk | Low risk | Low risk | Some concerns | Low risk | Some concerns |
| 31 | Qureshi et al. (2020) | Low risk | Low risk | Low risk | Low risk | Low risk | Low risk | Low risk |
| 32 | Sevene et al. (2020) | Low risk | Low risk | Low risk | Low risk | Low risk | Low risk | Low risk |
| 33 | Singh et al. (2020) | Low risk | Low risk | Low risk | Low risk | Low risk | Low risk | Low risk |
| 34 | Ugwa et al. (2020) | Low risk | Low risk | Low risk | Low risk | Low risk | Low risk | Low risk |
| 35 | Vanhuyse et al. (2022) | Low risk | Low risk | Low risk | Low risk | Low risk | Low risk | Low risk |
| 36 | von Dadelszen et al. (2020) | Low risk | Low risk | Low risk | Low risk | Low risk | Low risk | Low risk |
| 37 | Xie et al. (2018) | Low risk | Low risk | Low risk | High risk | Low risk | Low risk | High risk |
| 38 | Zhou et al. (2016) | Low risk | Low risk | Low risk | Low risk | Some concerns | Low risk | Some concerns |
| 39 | Zurovac et al. (2011) | Low risk | Low risk | Low risk | Low risk | Low risk | Low risk | Low risk |

**Table A5b** Risk of bias assessment of included studies based on JBI Critical Appraisal Tools (quasi-experimental, before-and-after studies with control group)

|  | Quasi-experimental Study (JBI) | Cause/ effect clarity | Similar comparison | Exposures other than intervention | Control group | Pre/post measures | Complete follow-up | Identical outcome measure* | Reliable outcome measure | Appropriate statistical analysis | Overall grade |
| --- | --- | --- | --- | --- | --- | --- | --- | --- | --- | --- | --- |
| 1 | Aksoy Derya et al. (2020) | Yes | Yes | No | Yes | Yes | Yes | Yes | Yes | Yes | 9/9 |
| 2 | Anitasari and Andrajiti (2017) | Yes | Yes | No | Yes | Yes | Unclear | Yes | Unclear | Yes | 7/9 |
| 3 | Chowdhury et al. (2019) | Yes | Yes | Unclear | Yes | No | Unclear | Yes | Yes | Yes | 6/9 |
| 4 | Coleman et al. (2017) | Yes | Yes | No | Yes | Yes | Unclear | Yes | Yes | No | 7/9 |
| 5 | Coleman et al. (2020) | Yes | Yes | No | Yes | No | Yes | Yes | Yes | Yes | 8/9 |
| 6 | Fahami et al. (2014) | Yes | Yes | No | Yes* | Yes | Yes | Yes | Yes | No | 7/9 |
| 7 | Fikawati et al. (2019) | Yes | Yes | No | Yes | No | No | Yes | Yes | Yes | 7/9 |
| 8-9 | Fotso et al. (2015a and 2015b) | Yes | No | Yes | Yes | Yes | No | Yes | Yes | Yes | 6/9 |
| 10 | Gong et al. (2020) | Yes | No | No | Yes | Yes | No | Yes | Yes | Yes | 7/9 |
| 11 | Ilozumba et al. (2018) | Unclear | No | No | Yes | Yes | Yes | Yes | Yes | Yes | 7/9 |
| 12 | Jerin et al. (2020) | Yes | Yes | No | Yes | Yes | Yes | Yes | Yes | Yes | 9/9 |
| 13-14 | Jiang et al. (2014) and (2019) | Yes | No | No | Yes | Yes | Unclear | Yes | Yes | Yes | 6/9 |
| 15 | Kiani et al. (2021) | Yes | Yes | No | Yes | Yes | Yes | Yes | Yes | Yes | 9/9 |
| 16 | Martinez-Fernandez et al. (2015) | Yes | Yes | No | Unclear | Yes | Unclear | Yes | Yes | Yes | 7/9 |
| 17 | Maslowsky et al. (2016) | Yes | Yes | No | Yes | Yes | No | Yes | Unclear | Yes | 7/9 |
| 18 | Masoi et al. (2019) | Yes | Yes | No | Yes | Yes | Yes | Yes | Yes | Yes | 9/9 |
| 19 | Mohamadirizi et al. (2014) | Yes | Yes | No | Yes | Yes | Yes | Yes | Yes | Yes | 9/9 |
| 20-21 | Murthy et al. (2019 and 2020) | Yes | No | No | Yes | Yes | No | Yes | Yes | Yes | 7/9 |
| 22 | Nemerimana et al. (2021) | Yes | Yes | No | Yes | Yes | Yes | Yes | Yes | Yes | 9/9 |
| 23 | Nguyet et al. (2021) | Yes | Yes | No | Yes | Yes | No | Yes | Yes | Yes | 8/9 |
| 24 | Oladepo et al. (2020) | Yes | No | Unclear | Yes | Yes | Unclear | Yes | Yes | Yes | 6/9 |
| 25 | Olajubu et al. (2020) | Yes | No | Unclear | Yes | Yes | No | Yes | Yes | Yes | 6/9 |
| 26 | Onono et al. (2019) | Yes | Unclear | No | Yes | Yes | Unclear | Yes | Yes | No | 6/9 |
| 27 | Parsa et al. (2018) | Yes | Yes | No | Yes | Yes | Yes | Yes | Yes | Yes | 9/9 |
| 28 | Prieto et al. (2017) | Yes | Unclear | No | Yes | Yes | No | Yes | Yes | Yes | 7/9 |
| 29 | Prinja et al. (2017) | Yes | Yes | No | Yes | Yes | Yes | Yes | Yes | Yes | 9/9 |
| 30 | Ruton et al. (2018) | Yes | Unclear | No | Yes | Yes | No | Yes | Yes | Yes | 7/9 |
| 31 | Schwartz et al. (2015) | Yes | Yes | No | Yes | No | Yes | Yes | Unclear | Yes | 7/9 |
| 32 | Shiferaw et al (2016) | Yes | Unclear | No | Yes | Yes | Unclear | Yes | Yes | Yes | 7/9 |
| 33 | Short et al. (2020) | Yes | Yes | No | Yes | Yes | Yes | Yes | Yes | Yes | 9/9 |
| 34 | Simonyan et al. (2013) | Yes | No | No | Yes | Yes | Yes | Yes | Unclear | Yes | 7/9 |
| 35 | Uddin et al. (2016) | Yes | Yes | No | Yes | Yes | Yes | Unclear | Unclear | Yes | 7/9 |
| 36 | Watterson et al. (2020) | Yes | No | Unclear | Yes | No | No | Yes | Yes | Unclear | 4/9 |
| 37 | Zhou et al. (2020) | Yes | Yes | No | Yes | Yes | No | Yes | Yes | Yes | 7/9 |

^*^No comparison between intervention and control, only pre-post comparisons within the same group.
